# A Donor–Acceptor 10-Cycloparaphenylene and Its Use as an Emitter in an Organic Light-Emitting Diode

**DOI:** 10.1021/acs.orglett.3c00127

**Published:** 2023-02-06

**Authors:** Dongyang Chen, Yoshimasa Wada, Yu Kusakabe, Liansheng Sun, Eiichi Kayahara, Katsuaki Suzuki, Hiroyuki Tanaka, Shigeru Yamago, Hironori Kaji, Eli Zysman-Colman

**Affiliations:** † Organic Semiconductor Centre, EaStCHEM School of Chemistry, University of St. Andrews, St. Andrews, Fife KY16 9ST, United Kingdom; ‡ Institute for Chemical Research, Kyoto University, Uji 611-0011, Japan

## Abstract

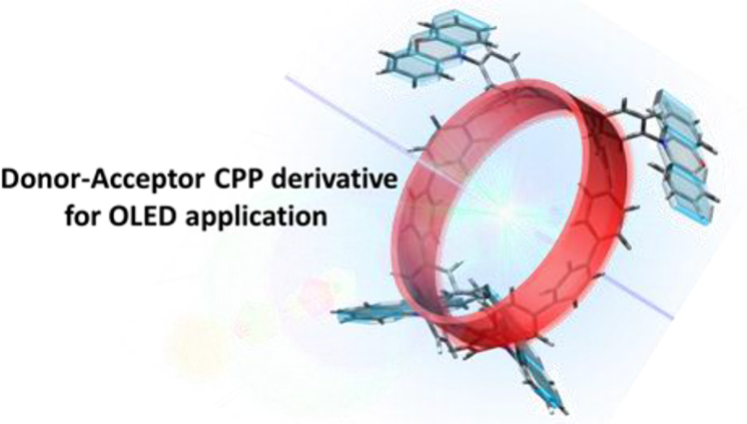

Here, we explored the possibility of using cycloparaphenylenes (CPP) within a donor–acceptor TADF emitter design. **4PXZPh-[10]CPP** contains four electron-donating moieties connected to a **[10]CPP**. In the 15 wt % doped in CzSi film, **4PXZPh-[10]CPP** showed sky-blue emission with λ_PL_ = 475 nm, Φ_PL_ = 29%, and triexponential emission decays with τ_PL_ of 4.4, 46.3, and 907.8 ns. Solution-processed OLEDs using **4PXZPh-[10]CPP** exhibited sky-blue emission with an λ_EL_ of 465 nm and an EQE_max_ of 1.0%.

Cycloparaphenylenes (CPPs) are the smallest building blocks of “armchair” carbon nanotubes and have attracted increasing attention due to their naturally curved nanohoop structure, which make them well suited as templates for host–guest chemistry and the bottom-up synthesis of carbon nanotubes.^2−4^ Moreover, their curved and radially oriented *π-*system has generated increasing interest from the organic semiconductor community.^5−7^ The aesthetically beautiful structure belies the significant embedded strain that results from the distorted benzene rings in the nanohoop, particularly the smaller analogues, which renders their synthesis challenging. The first CPPs (**[9]CPP**, **[12]CPP**, and **[18]CPP**) were synthesized using a strategy where cyclohexadienes were employed to afford intermediates that could more easily accommodate the curved structure prior to aromatization in the final step.^8^ A complementary strategy involves the formation of platinum complexes as precursors that then can be demetalated to form the CPP by reductive elimination. Seminal contributions from Bertozzi and Jasti et al.,^8^ Itami et al.,^9^ and Yamago et al.,^10^ among others, have demonstrated how the synthesis of CPPs can be scaled and how these structures can be elaborated further to generate compounds with desirable optoelectronic properties.^11−17^

The optoelectronic properties of CPPs can be tuned by decoration with electron-donating or -withdrawing groups.^18^ Jasti et al. incorporated the electron acceptor benzothiadiazole (BT) into the ring to construct donor–acceptor macrocycles **BT[10]CPP** (Figure 1).^19^ The DFT calculations revealed that the LUMO of **BT[10]CPP** is localized on the acceptor moiety and the LUMO level is stabilized by 0.8 eV compared to the reference **[10]CPP**, while the HOMOs are located on the remaining benzene rings on the macrocycle and are slightly destabilized compared to that of **[10]CPP**.^19^ The donor–acceptor (D–A) character of **BT[10]CPP** is evident in the absorption spectrum where a new band was observed at 450 nm, which correlates to the HOMO → LUMO transition that is of charge transfer (CT) character as assigned by time-dependent DFT (TD-DFT) calculations. The emission spectrum of **BT[10]CPP** is red-shifted by more than 100 nm (3946 cm^–1^) with a maximum emission wavelength (λ_PL_) of 571 nm and a a photoluminescence quantum yield (Φ_PL_) of 59% in dichloromethane (DCM), compared to λ_PL_ of 466 nm and Φ_PL_ of 65% for **[10]CPP**; moreover, the emission spectrum red-shifts with increasing solvent polarity, which is consistent with emission from a state of CT character.^19^ Computational studies from Sancho-García et al. provided insight that incorporation of an electron-withdrawing group not only can affect the HOMO/LUMO distribution and energies but also can tune the singlet–triplet energy gap (Δ*E*_ST_).^20^ For the proposed molecule *N***-methylaza-[8]CPP**^**+**^, where a methylpyridinium unit is incorporated into the CPP skeleton and used as an electron acceptor, the DFT calculations showed that the LUMO of *N***-methylaza-[8]CPP**^**+**^ is located on the methylpyridinium unit as well as the adjacent benzene rings and the HOMO is located across the remaining benzene rings. As a result, the Δ*E*_ST_ was predicted to decrease from 0.50 eV for **[8]CPP** to 0.23 eV for *N***-methylaza-[8]CPP**^**+**^.^20^ This is the first evidence for the potential of this class of compounds to exhibit thermally activated delayed fluorescence (TADF), where a small Δ*E*_ST_ is required to promote reverse intersystem crossing (RISC) at ambient temperatures.^20^

**Figure 1:**
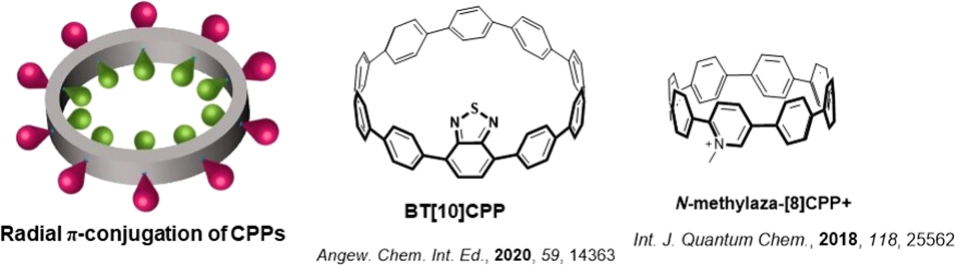
Molecular structures of cycloparaphenylene based D–A emitters. Middle and right images reprinted with permission from refs (19) and (20). Copyrights 2020 and 2017 Wiley.

TADF compounds have generated much interest as these can be used as emitters in electroluminescent devices that are capable of harvesting both singlet and triplet excitons and their conversion to light.^21−23^ When the RISC occurs from the lowest excited triplet (T_1_) to the lowest excited singlet (S_1_) states, the RISC rate constant (*k*_RISC_) is proportional to |*V*_SOC_|^2^ × exp[−(Δ*E*_ST_)^2^], where |*V*_SOC_|^2^ is the spin–orbit coupling matrix element between T_1_ and S_1_ states and Δ*E*_ST_ is the energy gap between them.^24^ A common strategy to enhance *k*_RISC_ and promote TADF is to minimize the Δ*E*_ST_, which can be achieved by reducing the overlap integral between the HOMO and LUMO.^25−27^ Previous work on CPPs has demonstrated that CPP derivatives can exhibit CT character in their excited states and moderate HOMO/LUMO separation by suitable decoration of the CPP core; however, none of these CPP derivatives have been reported to show TADF. To act as potential high-performance emitters for OLEDs, it would be of strong interest to develop TADF CPP derivatives.^20,28^ In this work, we employed DFT and TD-DFT calculations using the Tamm–Dancoff approximation (TDA-DFT) to design the CPP-based TADF emitter **4PXZPh-[10]CPP**, where four electron-donating moieties of 10-phenyl-10*H*-phenoxazine are connected to **[10]CPP**. The gas-phase calculations predict a Δ*E*_ST_ of 0.08 eV. Experimentally, **4PXZPh-[10]CPP** shows weak CT emission in polar solvents. The Φ_PL_ is 29% in the 15 wt % doped thin film in 9-(4-*tert*-butylphenyl)-3,6-bis(triphenylsilyl)-9*H*-carbazole (CzSi). We demonstrate the first CPP-based solution-processed organic light-emitting diode (OLED), which shows sky-blue emission with a maximum external quantum efficiency, EQE_max_, of 1.0%.

The synthesis of **4PXZPh-[10]CPP** started from the tetra-trifluoromethansulfonate-substituted [10]CPP (**4OTf-[10]CPP**), which was synthesized from the 2,5-di(3-butenyloxy)-1,4-benzquinone in 10 steps as previously reported.^29^**4OTf-[10]CPP** reacted with the electron-donor moiety BpinPhPXZ under Suzuki–Miyaura cross-coupling conditions to obtain **4PXZPh-[10]CPP** with a yield of 45% (Scheme 1).

**Scheme 1:**
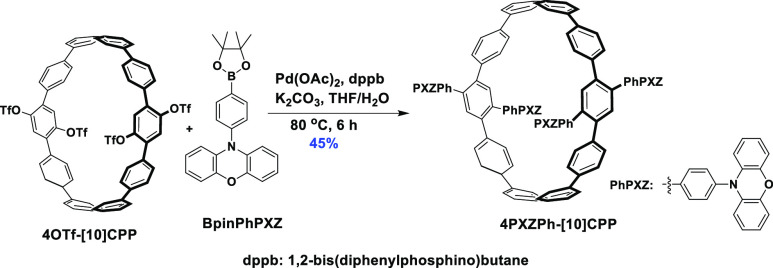
Synthesis of **4PXZPh-[10]CPP**

To gain insight into the electronic structure of **4PXZPh-[10]CPP**, DFT and TDA-DFT calculations were performed on both **4PXZPh-[10]CPP** and the reference compound **[10]CPP**. The ground state (S_0_), S_1_, T_1_, and higher triplet excited state (up to T_5_) energies were calculated in the gas phase at the PBE0/6-31g(d,p) level of theory.^30,31^ As shown in Figure 2a, the HOMO and the pseudodegenerate HOMO–1 of **4PXZPh-[10]CPP** are localized on two of the phenoxazine donors, respectively. The LUMO of **4PXZPh-[10]CPP** is delocalized across the CPP acceptor moiety. For parent **[10]CPP**, the HOMO and LUMO are distributed across the whole molecule (Figure 2b). The HOMO of **4PXZPh-[10]CPP** is destabilized to −5.49 eV compared to −5.66 eV for **[10]CPP**, while the LUMO of **4PXZPh-[10]CPP** is slightly destabilized to −1.88 eV compared to −1.94 eV for **[10]CPP**_,_ reflecting the small electronic communication between the donor moieties and the CPP skeleton. The spatially separated HOMO and LUMO ensures **4PXZPh-[10]CPP** possesses a small Δ*E*_ST_. TDA-DFT calculations indicate that the S_1_ energy of **4PXZPh-[10]CPP** (2.65 eV) is stabilized by 0.47 eV compared to that **[10]CPP** while the T_1_ energy (2.57 eV) is slightly destabilized by 0.01 eV compared to **[10]CPP**, thereby resulting in a Δ*E*_ST_ of 0.08 eV for **4PXZPh-[10]CPP**. The natural transition orbital (NTO) analysis of **4PXZPh-[10]CPP** reveals a CT transition for S_1_ from the PXZ donors to the CPP moiety, while the highest occupied NTO for T_1_ is delocalized over the whole molecule and the lowest unoccupied NTO is located on the CPP moiety, which suggest a hybridized local and charge-transfer (HLCT) excited state (Figure S7).

**Figure 2:**
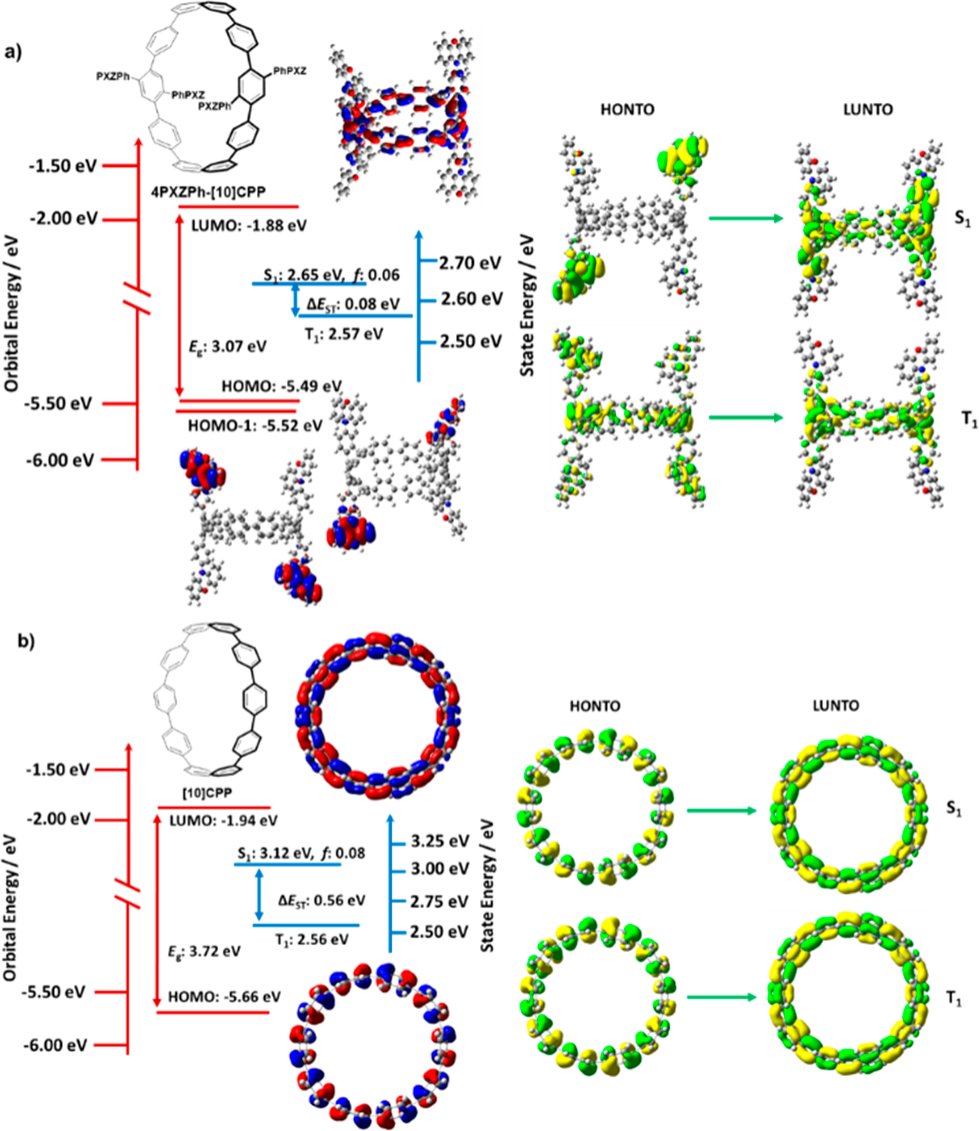
Theoretical modeling of the energies of the HOMO/LUMO and the S_1_ and T_1_ states of (a) **4PXZPh-[10]CPP** and (b) reference compound **[10]CPP** in the gas phase and the electron density distribution of the frontier molecular orbitals (isovalue = 0.02).

Cyclic voltammetry (CV) and differential pulse voltammetry (DPV) were measured in dichloromethane (DCM) with *n*-Bu_4_NPF_6_ as the supporting electrolyte to experimentally ascertain the HOMO and LUMO energy levels. The electrochemical behavior is detailed in the Supporting Information.

The photophysical properties of **4PXZPh-[10]CPP** were investigated in both solution and films. Room-temperature ultraviolet–visible (UV–vis) absorption and PL spectra of **4PXZPh-[10]CPP** and **[10]CPP** in dilute toluene solution are shown in Figure 3a. The absorption profile of **4PXZPh-[10]CPP** is similar to that of **[10]CPP** with a slightly stronger absorption (ε = 1.0 × 10^2^ M^–1^ cm^–1^) at 400 nm, which is ascribed to a mixed LE and weak intramolecular CT (ICT) transition from HOMO to LUMO according to the TDA-DFT calculations. **[10]CPP** exhibits strong absorption at 340 nm, which is ascribed to a LE transition distributed across the CPP skeleton, while for **4PXZPh-[10]CPP** the extra LE transition on the phenyl phenoxazine donor moieties are responsible for the moderately stronger and slightly blue-shifted absorption at 340 nm. The PL spectrum of **4PXZPh-[10]CPP** is broad, unstructured, and red-shifted at λ_PL_ = 508 nm with Φ_PL_ of 34% compared to the vibrationally structured spectrum of **[10]CPP** at λ_PL_ = 470 nm with Φ_PL_ of 77%. The profiles of the two PL spectra suggest that the emissive state of **4PXZPh-[10]CPP** is CT in nature while that of **[10]CPP** is LE in nature. The emission spectrum of **4PXZPh-[10]CPP** is bathochromically shifted and becomes broader with increasing solvent polarity, behavior that is consistent with an emissive state of CT character (Figures 3b).

**Figure 3:**
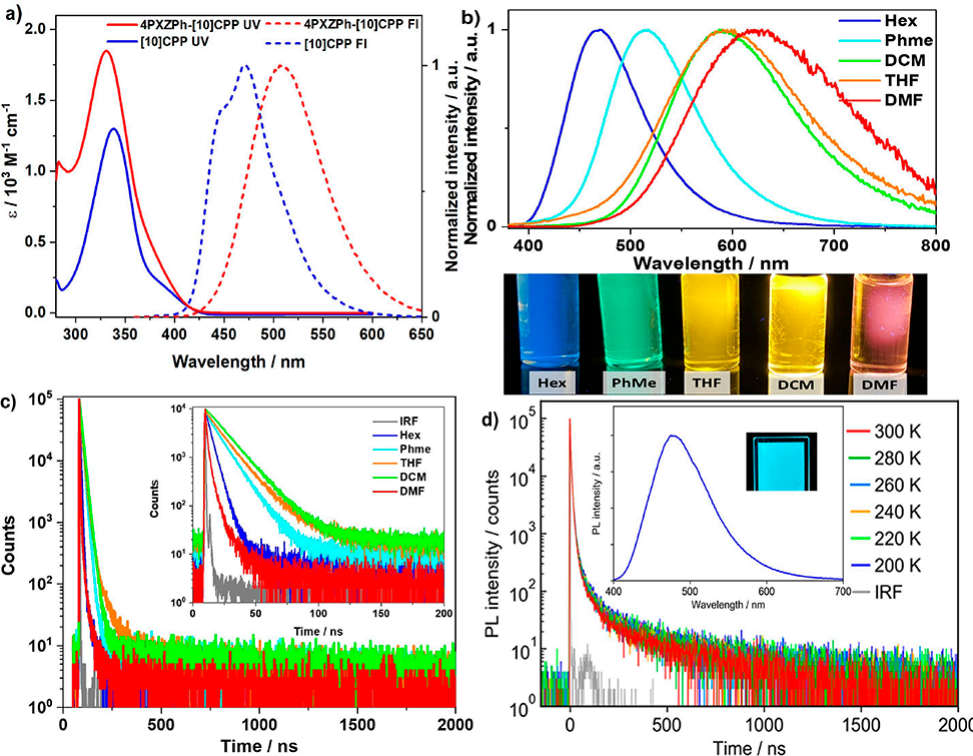
(a) Absorption and normalized emission spectra of **4PXZPh-[10]CPP** and **[10]CPP** in toluene solution (10^–5^ M), the excitation wavelength, λ_exc_ = 340 nm, (b) solvatochromic emission study of **4PXZPh-[10]CPP** (λ_exc_ = 375 nm), (c) transient PL decays of **4PXZPh-[10]CPP** in different solvents (λ_exc_ = 375 nm), and (d) emission spectra (insert) and temperature-dependent transient PL decay spectra of **4PXZPh-[10]CPP/**CzSi film (λ_exc_ = 340 nm).

The time-resolved PL decays of **4PXZPh-[10]CPP** were measured in solvents of different polarity. In nonpolar solvents of hexane (HEX), **4PXZPh-[10]CPP** exhibits a prompt lifetime (τ_p_) of 4.4 ns, while in more polar solvents such as toluene (PhMe), DCM, and tetrahydrofuran (THF), the τ_p_ values increase to 11.0, 14.5, and 13.6 ns, respectively. In the polar solvent dimethylformamide (DMF), the τ_p_ reduces to 3.1 ns. However, no delayed emission is observed in any of the solvents within a time window of 2 μs. The S_1_ and T_1_ energies of **4PXZPh-[10]CPP** were determined in both PhMe and DCM in 77 K. As shown in Figure S11, derived from the onset of the prompt fluorescence, the S_1_ of **4PXZPh-[10]CPP** in DCM is stabilized to 2.75 eV compared to 2.93 eV in PhMe, which led to a smaller Δ*E*_ST_ of 0.50 eV in DCM than 0.68 eV in PhMe as the T_1_ is 2.25 eV in both solvents. However, compared to the TDA-DFT predicted excited-state energies (S_1_, 2.65 eV; T_1_, 2.57 eV), the T_1_ measured in both toluene and DCM are strongly stabilized while the S_1_ measured in toluene and DCM are stabilized by 0.28 and 0.10 eV, respectively, and this leads to a much larger experimentally determined Δ*E*_ST_, which prohibits RISC from readily occurring at room temperature.

We next investigated the photophysics of **4PXZPh-[10]CPP** in doped films. The host 9-(4-(*tert*-butyl)phenyl)-3,6-bis(triphenylsilyl)-9*H*-carbazole (CzSi) was chosen due to its suitable HOMO/LUMO levels and high triplet state energies. In the 15 wt % doped CzSi film, **4PXZPh-[10]CPP** shows blue emission with λ_PL_ = 475 nm, Φ_PL_ = 29%, and τ_1_ = 4.4 ns, τ_2_ = 46.3 ns, and τ_3_ = 907.8 ns (Figure 3d). The temperature dependence of the transient PL decay in CzSi film was performed from 300 to 200 K. However, the delayed emission component in these films exhibited an increase with decreasing temperature, which implies that the emission is not TADF.^32−34^

With a view to exploring the potential of **4PXZPh-[10]CPP** as an emitter in OLEDs, a solution-processed OLED was fabricated using the architecture (Figure 4a) indium tin oxide (ITO) (50 nm)/poly(3,4-ethylenedioxythiophene)polystyrene sulfonate (PEDOT:PSS) (40 nm)/poly(9-vinylcarbazole) (PVK) (15 nm)/10 wt % **4PXZPh-[10]CPP**:CzSi/dibenzo[*b*,*d*]furan-2,8-diylbis(diphenylphosphine oxide) (PPF) (5 nm)/1,3-bis[3,5-di(pyridin-3-yl)phenyl]benzene (BmPyPhB) (45 nm)/lithium quinolin-8-olate (Liq) (1 nm)/Al (80 nm). PEDOT:PSS, PVK, and the emitting layers (EMLs) were deposited by spin-coating, and the other layers were thermally vacuum-deposited. In the device, PEDOT:PSS and PVK were used as a hole injection and transporting layers, respectively, BmPyPhB was used as an electron transporting and injection layer, and PPF was used as an exciton blocking layer. The fabricated OLED exhibits sky-blue emission with λ_EL_ = 465 nm, and the EQE_max_ of 1.0% (Figure 4b). The theoretical EQE_max_ (η_ext_) is deduced from eq 1.
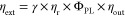
1Here, γ, η_r_, and η_out_ are the charge carrier balance, emissive exciton production efficiency, and light out coupling efficiency, respectively. Assuming that **4PXZPh-[10]CPP** is a conventional fluorescence material, and γ, η_r_, and η_out_ are unity, 0.25, and 0.2, respectively, considering that Φ_PL_ is 0.29, the experimental EQE_MAX_ is close to η_ext_. Although the device performance is below satisfactory, this is the first report of an electroluminescent device employing a donor–acceptor CPP-based emitter material.

**Figure 4:**
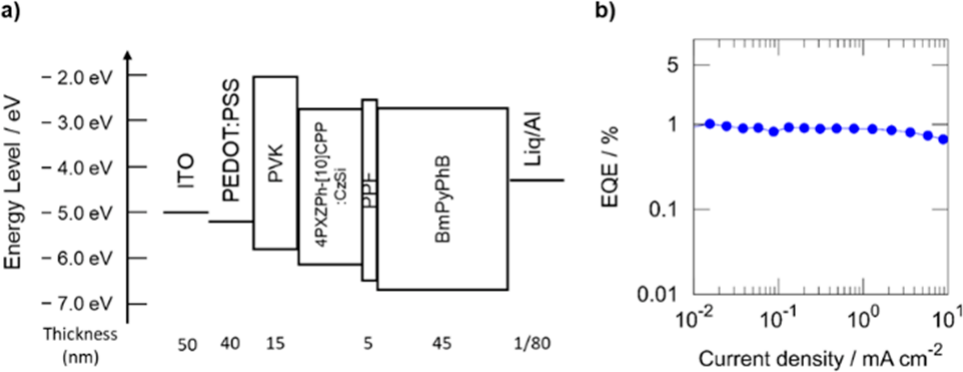
(a) Device stack of **4PXZPh-[10]CPP**:CzSi based OLEDs. (b) EQE–current density characteristics.

In summary, we designed a D–A CPP compound that was expected to show TADF. The DFT calculations revealed that this compound possesses a suitable HOMO/LUMO separation and the TDA-DFT calculation predicted a small Δ*E*_ST_ of 0.08 eV. **4PXZPh-[10]CPP** exhibited green emission in PhMe with λ_PL_ = 508 nm, Φ_PL_ = 34%, and a prompt lifetime of 11.0 ns. In the ambipolar host CzSi, **4PXZPh-[10]CPP** is moderately bright and showed sky-blue emission with λ_PL_ = 475 nm and Φ_PL_ = 29% in the 15 wt % doped CzSi film. The solution-processed OLEDs based on **4PXZPh-[10]CPP** showed low efficiency due to a combination of low Φ_PL_ and inefficient RISC. The device with **4PXZPh-[10]CPP** in CzSi as the **EML** exhibited sky-blue emission, with λ_EL_ of 465 nm and an EQE_max_ of 1.0% at 1 mA cm^–2^.

## Data Availability

The research data supporting this publication can be accessed at https://doi.org/10.17630/6c910af5-0115-4e0f-8de8-faa484e406d1.

## Supporting Information Available

Experimental data, ^1^H NMR, ^13^C NMR, HRMS, HPLC report, and elemental analysis for all new compounds. Computation details, Natural transition orbitals analysis, UV–vis and fluorescence spectra, steady-state PL spectra, emission spectra, chromatogram, photophysical measurements, cyclic voltammetry, CV plot, and device fabrication details (PDF)
Atomic coordinates (TXT)

## Notes

This manuscript has been deposited to preprint repository ChemRxiv.^1^
