# Supplementary material for: A Donor–Acceptor 10-Cycloparaphenylene and Its Use as an Emitter in an Organic Light-Emitting Diode
Source: Org Lett. 2023 Feb 6;25(6):998–1002. doi: 10.1021/acs.orglett.3c00127 (PMC9942195; doi:10.1021/acs.orglett.3c00127)
Supplement: Supplementary file 1 — ol3c00127_si_001.pdf [file ol3c00127_si_001.pdf]

Supplementary Information

A Donor-Acceptor 10-Cycloparaphenylene and Its Use as an Emitter in an Organic Light-Emitting Diode

Dongyang Chen,[a] Yoshimasa Wada,[b] Yu Kusakabe,[b] Liansheng Sun,[b] Eiichi Kayahara,[b] Katsuaki Suzuki,[b] Hiroyuki Tanaka,[b] Shigeru Yamago,*[b] Hironori Kaji,*[b] Eli Zysman-Colman*[a]

[a] Organic Semiconductor Centre, EaStCHEM School of Chemistry, University of St. Andrews, St. Andrews, Fife, KY16 9ST, United Kingdom.

[b] Institute for Chemical Research, Kyoto University, Uji 611-0011, Japan

Table of Contents

Synthesis and Chemical Characterization S3

Theoretical Calculations S22

Electrochemistry measurements S24

Photophysical experimental Details S25

Device Fabrication and Measurement S27

NMR Spectra of Intermediates S30

Ground State Optimization Summary S44

Cartesian Coordinates S45

Synthesis and Chemical Characterization

General Synthetic Procedures

Commercial chemicals were used without further purification. All reactions were performed using standard Schlenk techniques under nitrogen atmosphere with dry solvents. Column chromatography was performed using silica gel (Silia-P from Silicycle, 60 Å, 40 to 63 μm). Preparative GPC was performed using JAIGEL 1H, 2H, and 2.5H polystyrene columns (Japan Analytical Industry Co., Ltd.) with $CHCl_3$ as the eluent. Analytical thin layer chromatography was performed with silica plates with polymer (250 μm with indicator F-254), and compounds were visualized under UV light. ^{1}H and ^{13}C solution-phase NMR spectra were recorded on a Bruker Avance spectrometer operating at 11.7 T (Larmor frequencies of 400 MHz and 100MHz or 500 MHz and 125 MHz, respectively) in chloroform-*d* ($CDCl_3$) or dimethyl sulfoxide-*d*6 (*d*-DMSO) solvent. The following abbreviations have been used for multiplicity assignments: "s" for singlet, "d" for doublet, "t" for triplet, "m" for multiplet, and "br" for broad. Melting points (Mps) were recorded using open-ended capillaries on an electrothermal Mp apparatus and are uncorrected. Electrospray ionization time-of-flight mass (ESI-TOF MS) spectrum was recorded on a spectrometer in the positive or negative mode. A sample was injected as a CH_2Cl_2 solution. Matrix-assisted laser-desorption ionization (MALDI)-TOF MS spectrum was obtained on a spectrometer in the positive reflection mode and at 20 kV acceleration voltage. Samples were prepared from a THF solution by mixing a sample (1 mg/mL) and dithranol (10 mg/mL) in a 1:1 ratio. High-performance liquid chromatography (HPLC) analysis was conducted on a Shimadzu Prominence Modular HPLC system. HPLC traces were measured using an ACE Excel 2 C18 analytical column.

Synthesis of BrPhPXZ (10-(4-bromophenyl)-10H-phenoxazine)

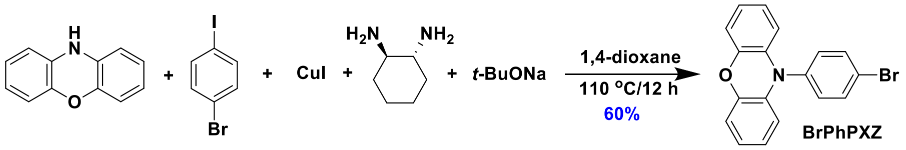

BrPhPXZ was synthesized according to a literature procedure.[1] To a 250 mL three-neck flask were added phenoxazine (1.8 g, 10 mmol, 1 equiv.), 1-bromo-4-iodobenzene (4.2 g, 15 mmol, 1.5 equiv.), copper (I) iodide (190 mg, 1 mmol, 0.1 equiv.), (±)-*trans*-1,2-diaminocyclohexane (230 mg, 2 mmol, 0.2 equiv.), sodium *tert*-butoxide (3.0 g, 30 mmol, 3 equiv.) and 50 mL of 1,4-dioxane. Oxygen was excluded from the reaction by three freeze-pump-throw cycles. After warming to room temperature, the suspension was heated to 110 °C and stirred for 12 hours in an oil bath. After cooling, 100 mL of saturated ammonium chloride solution was poured into the reaction mixture and the organic phase was extracted with DCM (50 mL×3). The combined organic layers were dried with anhydrous magnesium sulfate. The organic solvent was removed under reduced pressure and the crude product was purified by silica gel column chromatography. 9DCM/Hexane=1/10) to afford **BrPhPXZ** as a white solid (1.3 g).

Yield: 60%. **R_f**: 0.67 (10% DCM/hexanes). **Mp**: 190-192 °C. **^{1}H NMR (400 MHz, $CDCl_3$) δ (ppm)**: 7.76 (d, J = 8.6 Hz, 2H), 7.27 (d, J = 8.5 Hz, 2H), 6.88 – 6.66 (m, 4H), 6.63 (td, J = 7.6, 1.9 Hz, 2H), 5.94 (dd, J = 7.8, 1.5 Hz, 2H). **^{13}C NMR (100 MHz, $CDCl_3$) δ (ppm)**: 143.9, 138.1, 134.4, 134.0, 132.8, 123.3, 122.4, 121.6, 115.6, 113.2. **HRMS** (ESI) m/z: **$[M+H]^+$** Calcd for $C_{18}H_{13}NOBr$ 338.0102; Found 338.0110. The characterization matches that previously reported.[1]

Synthesis of BpinPhPXZ (10-(4-(4,4,5,5-tetramethyl-1,3,2-dioxaborolan-2-yl) phenyl)-10*H*-phenoxazine)

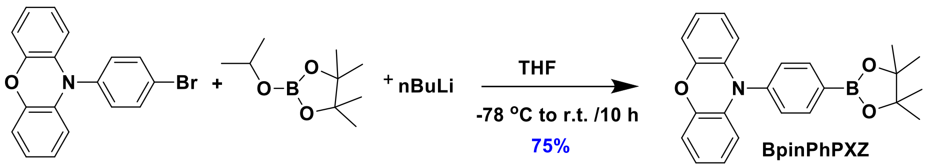

BpinPhPXZ was synthesized as literature.[2] To a 100 mL two-neck flask, 10-(4-bromophenyl)-10H-phenoxazine (1.7 g, 5 mmol, 1 equiv.) was added and degassed by three cycles of vacuum-nitrogen purging. 30 mL of dry THF was then injected into the flask. The solution was cooled to -78 °C using a dry ice/acetone bath and *n*-butyllithium (1.6 M) (3.4 mL, 5.5 mmol, 1.1 equiv.) was added dropwise into the solution. The mixture was stirred at -78 °C for 1 hour and 2-isopropoxy-4,4,5,5-tetramethyl-1,3,2-dioxaborolane (1.1 g, 6 mmol, 1.2 equiv.) was injected into the solution. The mixture was stirred at -78 °C for 30 min before warming to room temperature. The reaction mixture was stirred overnight. The reaction was quenched with MeOH (1 mL) and the reaction was poured into icy water followed by extraction with DCM (50 mL×3). The combined organic layers were dried with anhydrous magnesium sulfate. The organic solvent was removed under reduced pressure and the crude product was purified by silica gel column chromatography (DCM/Hexane=2/8) to afford **BpinPhPXZ** as a white solid (1.4 g).

Yield: 75%. $\mathbf{R_f}$: 0.62 (20% DCM/hexanes). **Mp**: 155-157 °C. **^{1}H NMR (500 MHz, $CDCl_3$) δ (ppm)**: 8.05 (d, J = 8.2 Hz, 2H), 7.38 (d, J = 8.2 Hz, 2H), 6.70 (dd, J = 7.8, 1.6 Hz, 2H), 6.65 (td, J = 7.6, 1.5 Hz, 2H), 6.59 (td, J = 7.7, 1.7 Hz, 2H), 5.93 (dd, J = 7.9, 1.5 Hz, 2H), 1.41 (s, 12H). **^{13}C NMR (125 MHz, $CDCl_3$) δ (ppm)**: 143.9, 141.7, 137.5, 134.2, 130.1, 123.2, 121.3, 115.4, 113.3, 84.2, 24.9. **HRMS** (ESI) m/z: **$[M+H]^+$** Calcd for $C_{24}H_{25}NO_3B$ 385.1849; Found

385.1852. The characterization matches that previously reported.[2]

Synthesis of BuBQ (2,5-bis(but-3-en-1-yloxy) cyclohexa-2,5-diene-1,4-dione)

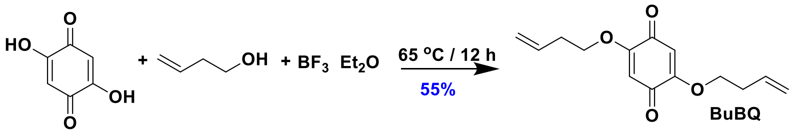

The synthesis of **BuBQ** follows the literature procedure.[3] To a 500 mL three-neck flask was added 2,5-dihydroxy-1,4-benzoquinone (14.0 g, 100 mmol, 1 equiv.) and the flask degassed by three cycles of vacuum-nitrogen purging. 3-buten-1-ol (37.1 g, 2 mol, 20 equiv.) and $BF_3{\cdot}OEt_2$ (30.0 mL, 220 mmol, 2.2 equiv.) were added and the mixture was stirred at 65 °C for 12 h in an oil bath. After cooling, the resulting mixture was filtered and washed with methanol to afford a brown crude product. The crude product was purified by silica gel column chromatograph (DCM/Hexane=1/1) to afford **BuBQ** as a yellow solid (8.5 g).

Yield: 55%. **R_f**: 0.54 (50% DCM/hexanes). **Mp**: 181-183 °C. **^{1}H NMR ($CDCl_3$, 400 MHz) δ (ppm)**: δ 5.85 (s, 4H), 5.21 (q, J = 1.5 Hz, 1H), 5.16 (p, J = 1.5 Hz, 2H), 5.13 (q, J = 1.3 Hz, 1H), 3.98 (t, J = 6.9 Hz, 4H), 2.62 (qt, J = 6.9, 1.4 Hz, 4H); **^{13}C NMR ($CDCl_3$, 100 MHz) δ (ppm)**: 181.7, 158.6, 132.8, 118.2, 106.0, 68.8, 32.4. **HRMS** (EI) m/z: **$[M+H]^+$** Calcd for $C_{14}H_{17}O_4$ 249.1127; Found 249.1121. The characterization matches that previously reported.[3]

Synthesis of DPBuBQ ((1''R,4''R)-4,4''''-dibromo-2'',5''-bis(but-3-en-1-yloxy)-[1,1':4',1'':4'',1''':4''',1''''-quinquephenyl]-1'',4''-diol)

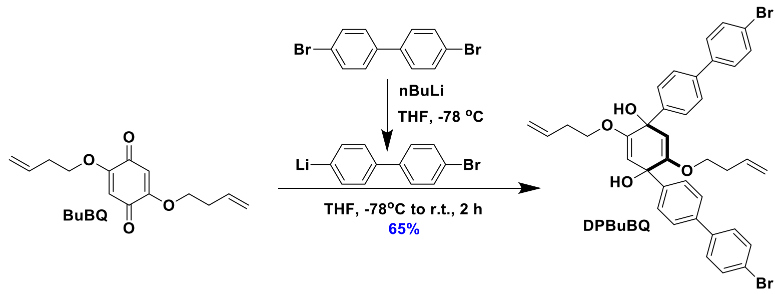

The synthesis of **DPBuBQ** follows the literature procedure.[3] To a 1 L three-neck flask was added 4,4'-dibromo-1,1'-biphenyl (31.1 g, 100 mmol, 4.0 equiv.) and the flask degassed by three cycles of vacuum-nitrogen purging before. THF (300 mL) was added to the flask. The solution was cooled down to -78 °C using a dry ice/acetone bath and *n*-butyllithium (1.6 M) (3.4 mL, 5.5 mmol, 1.1 equiv.) was added dropwise into the solution. The mixture was stirred at -77 °C for 1 hour and the solution was transferred via cannula to a -78 °C solution of **BuBQ** (6.2 g, 25 mmol, 1 equiv.) in THF (200 mL). The mixture was stirred at -78 °C for 1 h before warming to room temperature over 2 h. The reaction was quenched by saturated NH_4Cl solution (40 mL) and the mixture poured into icy water and then extracted with ethyl acetate (100 mL×3). The combined organic layers were dried with anhydrous sodium sulfate. The organic solvent was removed under reduced pressure and the crude product was purified by silica gel column chromatography (Ethyl actate/Hexane=1/9) to afford **DPBuBQ** as a white solid (48.8 g).

Yield: 65%. **R_f**: 0.44 (20% ethyl acetate/hexanes). **Mp**: 127-129 °C. **^{1}H NMR ($CDCl_3$, 400 MHz) δ (ppm)**: 7.56 (d, J = 8.4 Hz, 4H), 7.50 (d, J = 8.4 Hz, 8H), 7.46 (d, J = 8.4 Hz, 4H), 5.67 (ddt, J = 17.2, 10.4, 6.8 Hz, 2H), 5.10 (s, 2H), 5.00 (d, J = 18.8 Hz, 2H), 4.97 (d, J = 10.4 Hz, 2H), 3.83-3.92 (dt, J = 9.0, 7.0 Hz, 2H), 3.73-3.82 (dt, J = 9.0, 7.1 Hz, 2H), 3.03 (s, 2H), 2.35 (q, J = 6.8 Hz, 4H). **^{13}C NMR ($CDCl_3$, 100 MHz) δ (ppm)**: 154.2, 144.6, 139.6, 138.9, 137.9, 134.1, 131.9, 128.9, 128.6, 126.8, 126.2, 121.6, 117.1, 103.2, 74.0, 66.8, 33.0. **HRMS** (MALDI-TOF) m/z: **$[M+Na]^+$** Calcd for $C_{38}H_{34}Br_2O_4Na$ 735.0716; Found 735.0724. The

characterization matches that previously reported.[3]

Synthesis of TesBuBQ ((((1''R,4''R)-4,4''''-dibromo-2'',5''-bis(but-3-en-1-yloxy)-[1,1':4',1'':4'',1''':4''',1''''-quinquephenyl]-1'',4''-diyl)bis(oxy))bis(triethylsilane))

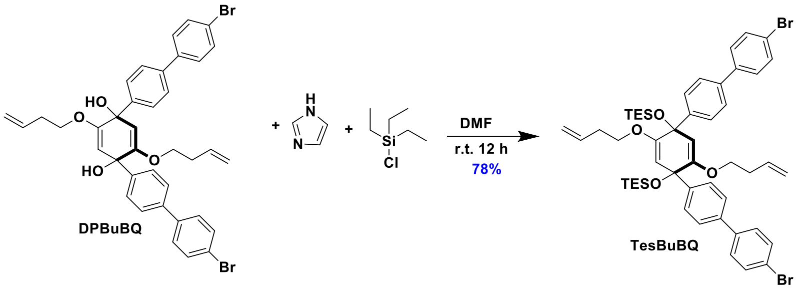

The synthesis of **TesBuBQ** follows the literature procedure.[3] To a 250 mL three-neck flask were added **DPBuBQ** (7.2 g, 10 mmol, 1 equiv.) and imidazole (3.4g, 50 mmol, 5 equiv.) and the flask degassed by three cycles of vacuum-nitrogen purging. DMF (50 mL) was then added followed by dropwise addition of triethylchlorosilane (5 mL, 30 mmol, 3 equiv.). The solution was stirred at room temperature for 12 h. The resulting mixture was poured into icy water and extracted by DCM (50 mL×3). The combined organic layers were dried with anhydrous sodium sulfate. The organic solvent was removed under reduced pressure and the crude product was purified by silica gel column chromatography (Ethyl acetate/Hexane=0.2/9.8) to afford **TesBuBQ** as a white solid (7.1 g).

Yield: 78%. **R$_f$**: 0.54 (10% ethyl acetate/hexanes). **Mp**: 135-136 °C. **^{1}H NMR ($CDCl_3$, 400 MHz) δ (ppm)**: 7.54 (d, J = 8.4 Hz, 4H), 7.46 (d, J = 8.8 Hz, 4H), 7.45 (s, 8H), 5.66-5.77 (m, 2H), 5.01 (d, J = 17.2 Hz, 2H), 4.97 (d, J = 10.1 Hz, 2H), 4.86 (s, 2H), 3.73-3.80 (dt, J = 9.2, 6.4 Hz, 2H), 3.61-3.69 (dt, J = 9.0, 7.1 Hz, 2H), 2.38 (q, J = 6.8 Hz, 4H), 0.97 (t, J = 8.0 Hz, 18H), 0.55-0.74 (m, 12H). **^{13}C NMR ($CDCl_3$, 100 MHz) δ (ppm)**: 153.9, 146.1, 139.9, 137.8, 134.5, 131.8, 128.6, 127.0, 126.0, 121.3, 116.2, 104.0, 66.5, 33.3, 7.2, 6.4. **HRMS** (MALDI-

TOF) m/z: **[M]**$^+$ Calcd for $C_{50}H_{62}Br_2O_4Si_2$ 942.2540; Found 942.2554. The characterization matches that previously reported.[3]

Synthesis of BpinBQ ((((1''R,4''R)-2'',5''-bis(but-3-en-1-yloxy)-4,4''''-bis(4,4,5,5-tetramethyl-1,3,2-dioxaborolan-2-yl)-[1,1':4',1'':4'',1''':4''',1''''-quinquephenyl]-1'',4''-diyl)bis(oxy))bis(triethylsilane))

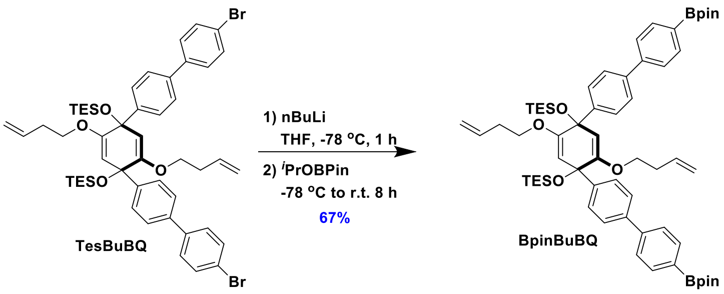

The synthesis of **BpinBuBQ** follows the literature procedure.[3] To a 250 mL three-neck flask was added **TesBuBQ** (4.7 g, 5 mmol, 1 equiv.) and the flask was degassed by three cycles of vacuum-nitrogen purging. Dry THF (30 mL) was then added. The solution was cooled to -78 °C using a dry ice/acetone bath and *n*-butyllithium (1.6 M) (8 mL, 12.5 mmol, 2.5 equiv.) was added dropwise into the solution. The mixture was stirred at -78 °C for 1 hour and tri*iso*propyl borate (3.1 mL, 15 mmol, 3 equiv.) was added dropwise. The mixture was stirred at -78 °C for 30 min before warming to room temperature and stirred overnight. The reaction was quenched with methanol (1 mL) and the reaction was poured into icy water followed by extraction with DCM (50 mL×3). The combined organic layers were dried with anhydrous sodium sulfate. The organic solvent was removed under reduced pressure and the crude product was washed with methanol and recrystallized from DCM/methanol = 1/20 to afford **BpinBuBQ** as white solid (3.2 g).

Yield: 67%. **R$_f$**: 0.44 (10% ethyl acetate/hexanes). **Mp**: 131-133 °C. **^{1}H NMR ($CDCl_3$, 400**

MHz) δ (ppm): 7.86 (d, J = 8.4 Hz, 4H), 7.62 (d, J = 8.4 Hz, 4H), 7.52 (d, J = 8.4 Hz, 4H), 7.46 (d, J = 8.4 Hz, 4H), 5.66-5.78 (ddt, J = 17.2, 10.4, 6.8 Hz, 2H), 5.18 (dq, J = 17.2, 1.7 Hz, 2H), 4.99 (dq, J = 10.4, 1.6 Hz, 2H), 4.87 (s, 2H), 3.73-3.82 (dt, J = 9.0, 6.6 Hz, 2H), 3.59-3.70 (dt, J = 9.0, 7.1 Hz, 2H), 2.38 (q, J = 6.8 Hz, 4H), 1.36 (s, 24 H), 0.97 (t, J = 8.0 Hz, 18 H), 0.57-0.79 (m, 12H). **^{13}C NMR ($CDCl_3$, 100 MHz) δ (ppm)**: 153.9, 146.0, 143.7, 139.3, 135.2, 134.5, 127.0, 126.8, 126.4, 126.3, 116.7, 104.0, 83.8, 75.3, 66.4, 33.3, 24.9, 7.2, 6.4. **HRMS** (MALDI-TOF) m/z: $[M]^+$ Calcd for $C_{62}H_{86}B_2O_8Si_2$ 1036.6047; Found 1036.6066. The characterization matches that previously reported.[3]

Synthesis of TesBu-8Ph2Ch ((5^1*R*, 5^4*R*, 10^1*S*, 10^4*S*)-5^2, 5^5, 10^2, 10^5-tetra(but-3-en-1-yloxy)-5^1, 5^4, 10^1, 10^4-tetra(oxytriethylsilane)-1, 2, 3, 4, 6, 7, 8, 9 (1, 4)-octabenzena-5, 10 (1, 4)-dicyclo hexane cyclodecaphane-5^2, 5^5, 10^2, 10^5-tetraene)

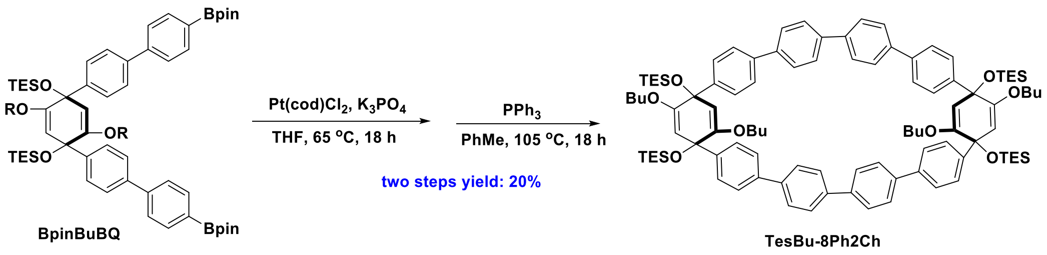

The synthesis of **TesBu-8Ph2Ch** follows the literature procedure.[3] To a 250 mL three-neck flask were added **BpinBuBQ** (2.1 g, 2 mmol, 1 equiv.), Pt(cod)Cl_2 (374 mg, 2 mmol, 1 equiv.), K_3PO_4 (2.2 g, 10 mmol, 5 equiv.) and the flask degassed by three cycles of vacuum-nitrogen purging. Dry THF (100 mL) was then added. The solution was stirred at 65 °C overnight in an oil bath, cooled to room temperature and then poured into water. The mixture was extracted with DCM (50 mL×3) and the combined organic layers were dried with anhydrous sodium sulfate. The organic solvent was removed under reduced pressure to afford the crude Pt-

complex product as white solid. The Pt-complex and triphenylphosphine (2.6 g, 10 mmol, 5 equiv.) were added to a 250 mL three-neck flask, which was then degassed by three cycles of vacuum-nitrogen purging. Toluene (100 mL) was added, and the solution was stirred at 105 °C overnight in an oil bath. The resulting mixture was washed with brine (20 mL) and extracted with DCM (50 mL×3). The organic phase was dried with by anhydrous sodium sulfate and removed under reduced pressure to afford the crude product as beige solid. The crude product was purified by silica gel column chromatography (DCM/hexane=1/5) and 2.5H polystyrene gel permeation chromatography (chloroform as eluent) to afford **TesBu-8Ph2Ch** as a white solid (400 mg).

Two steps yield: 20%. **R$_f$**: 0.54 (15% DCM/hexanes). **Mp**: 163 °C. **^{1}H NMR (CDCl$_3$, 400 MHz) δ (ppm)**: 7.47 (d, *J* = 8.4 Hz, 1H), 7.33 (d, *J* = 8.3 Hz, 1H), 7.08 (d, *J* = 8.5 Hz, 1H), 6.97 (dd, *J* = 11.2, 8.5 Hz, 1H), 6.03 – 5.83 (m, 0H), 5.34 – 5.03 (m, 2H), 3.89 (p, *J* = 8.7, 7.8 Hz, 1H), 2.61 (p, *J* = 6.8, 6.4 Hz, 1H), 1.00 (t, *J* = 7.9 Hz, 5H), 0.83 – 0.58 (m, 2H). **^{13}C NMR (CDCl$_3$, 100 MHz) δ (ppm)**: 156.0, 155.8, 140.0, 138.8, 134.5, 134.5, 127.6, 126.8, 126.8, 126.7, 126.1, 117.1, 104.2, 103.7, 75.1, 75.0, 66.8, 33.5, 7.2, 6.4. **HRMS** (MALDI-TOF) m/z: **[M]$^+$** Calcd for $C_{100}H_{124}O_8Si_2$ 1565.8401; Found 1565.8338. The characterization matches that previously reported.[3]

Synthesis of OHBu-8Ph2Ch ((5^1*R*, 5^4*R*, 10^1*S*, 10^4*S*)-5^2, 5^5, 10^2, 10^5-tetra(but-3-en-1-yloxy)-1, 2, 3, 4, 6, 7, 8, 9 (1, 4)-octabenzena-5, 10(1, 4)-dicyclohexanacyclodecaphane-5^2, 5^5, 10^2, 10^5-tetraene-5^1, 5^4, 10^1, 10^4-tetraol)

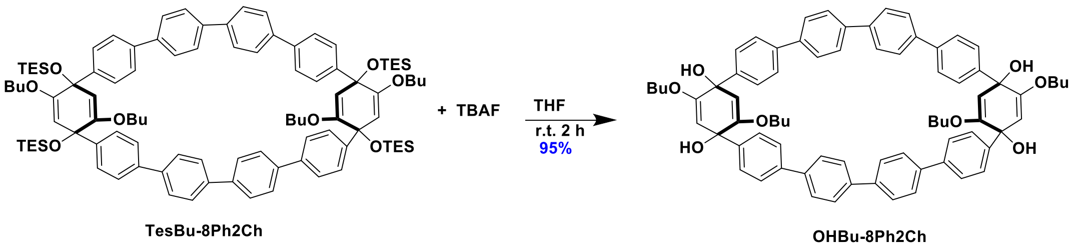

The synthesis of **OHBu-8Ph2Ch** follows the literature procedure.[3] To a 100 mL flask were added **TesBu-8Ph2Ch** (1.6 g, 1 mmol, 1 equiv.) and TBAF (1.00 M) (5 mL, 5 mmol, 5 equiv.) in THF (25 mL). The solution was stirred at room temperature for 2 hours and then poured into icy water. The precipitate was filtrated and washed with hexane to afford **OHBu-8Ph2Ch** as a white solid (1.2 g).

Yield: 67%. **R$_f$**: 0.30 (40% ethyl acetate/hexanes). **Mp**: 211-213 °C. **^{1}H NMR ($CDCl_3$, 400 MHz) δ (ppm)**: 7.76 (d, J = 8.5 Hz, 2H), 7.60 – 7.52 (m, 8H), 7.51 – 7.49 (m, 12H), 7.46 (d, J = 8.7 Hz, 8H), 7.34 (d, J = 8.5 Hz, 2H), 5.83 – 5.52 (m, 8H), 5.10 (s, 4H), 5.03 (d, J = 1.7 Hz, 2H), 5.00 – 4.92 (m, 2H), 3.88 (dt, J = 9.5, 6.6 Hz, 4H), 3.77 (dt, J = 9.6, 6.6 Hz, 4H), 3.03 (s, 4H), 2.36 (ddt, J = 8.1, 6.7, 3.3 Hz, 8H). **^{13}C NMR ($CDCl_3$, 100 MHz) δ (ppm)**: 155.6, 155.1, 142.3, 142.2, 139.5, 138.8, 134.11, 134.07, 127.6, 126.9, 126.6, 117.4, 117.4, 103.7, 102.9, 73.7, 73.6, 67.2, 67.2, 33.3, 33.2. **HRMS** (MALDI-TOF) m/z: **[M]$^+$** Calcd for $C_{76}H_{68}O_8$ 1108.4914; Found 1108.4908. The characterization matches that previously reported.[3]

Synthesis of BuO-[10]CPP ((10^4*R*, *E*)-2^2, 2^5, 7^2, 7^5-tetra(but-3-en-1-yloxy)-1, 2, 3, 4, 6, 7, 8(1, 4)-heptabenzena-5, 9, 10(1, 4)-tricyclohexanacyclodecaphane-5^2, 5^4, 9^1, 9^5, 10^1, 10^5-hexaene)

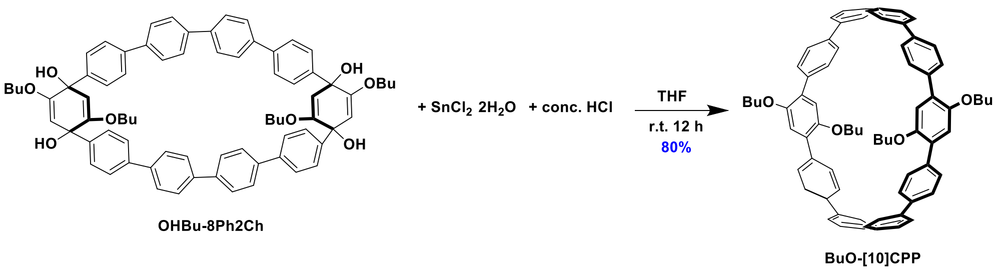

The synthesis of **BuO-[10]CPP** follows the literature procedure.[3] To a 250 mL flask containing a solution of $SnCl_2·H_2O$ (1.1 g, 5 mmol, 5 equiv.) in THF (50 mL) was added concentrated HCl (12 N) (0.8 mL, 10 mmol, 10 equiv.) and the reaction mixture stirred at room temperature for 1 hour. A solution of **OHBu-8Ph2Ch** (1.1 g, 1 mmol, 1 equiv.) in THF (20 mL) was added and the mixture was stirred at room temperature overnight. The reaction was quenched with 10% aqueous NaOH solution and extracted with ethyl acetate (50 mL×3). The combined organic layers were dried with anhydrous sodium sulfate. The organic solvent was removed under reduced pressure and the crude product was purified by silica gel column chromatography (DCM) to afford **BuO-[10]CPP** as a white solid (780 mg).

Yield: 80%. **R_f**: 0.64 (50% DCM/hexanes). **Mp**: 255-256 °C. **^{1}H NMR ($CDCl_3$, 400 MHz) δ (ppm)**: 7.61 (ddd, J = 10.3, 8.5, 1.8 Hz, 16H), 7.56 – 7.52 (m, 8H), 7.50 – 7.45 (m, 8H), 6.87 (s, 4H), 6.04 – 5.60 (m, 4H), 5.25 – 4.84 (m, 8H), 3.89 (td, J = 6.6, 1.8 Hz, 8H), 2.57 – 2.24 (m, 8H). **^{13}C NMR ($CDCl_3$, 100 MHz) δ (ppm)**: 151.3, 138.8, 138.6, 137.9, 136.6, 134.8, 129.6, 129.5, 127.6, 127.1, 126.6, 116.9, 116.6, 69.3, 34.0. **HRMS** (MALDI-TOF) m/z: **$[M]^+$** Calcd for $C_{76}H_{68}O_8$ 1040.4799; Found 1040.4793. The characterization matches that previously reported.[3]

Synthesis of DOBt-[10]CPP ((10^4*R*, *E*)-2^2, 2^5, 7^2, 7^5-tetra(3,4-dihydroxybutoxy)-1, 2, 3, 4, 6, 7, 8(1, 4)-heptabenzena-5, 9, 10(1, 4)-tricyclohexanacyclodecaphane-5^2, 5^4, 9^1, 9^5, 10^1,

10⁵-hexaene)

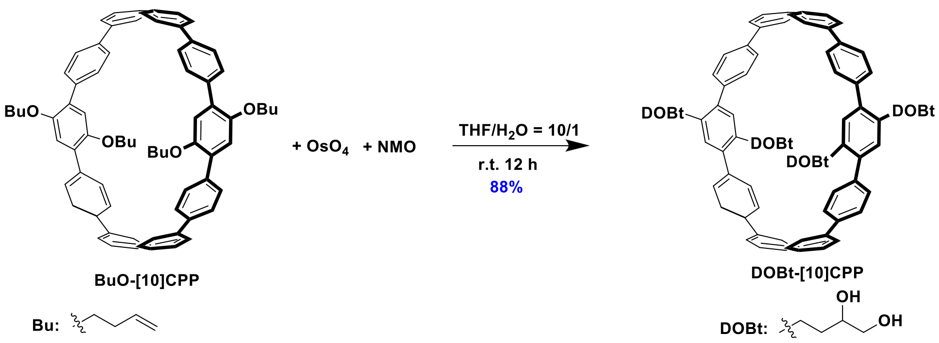

The synthesis of **DOBt-[10]CPP** follows the literature procedure.[4] To a 250 mL flask were added **BuO-[10]CPP** (1.0 g, 1 mmol, 1 equiv.), OsO_4 (0.1 mmol, 26 mg, 0.1 equiv.), and *N*-methylmorpholine *N*-oxide (NMP) (515 mg, 4.4 mmol, 4.4 equiv.). THF (100 mL) and H_2O (10 mL) were added and the solution was stirred at room temperature for 12 hours. The resulting solution was washed with 10% aqueous Na_2SO_3 solution (20 mL) and brine (30 mL) before extracted with ethyl acetate (50 mL × 3). The combined organic layers were dried with anhydrous sodium sulfate. The organic solvent was removed under reduced pressure to afford the crude product as a brown solid, which was purified by recrystallization from DCM/hexane = 1/5 to afford the product as a yellow solid (900 mg).

Yield: 80%. **R_f**: 0.40 (50% DCM/hexanes). **Mp**: 274-275 °C. **^{1}H NMR (DMSO-d$_6$, 400 MHz) δ (ppm)**: 7.73 (d, J = 8.4 Hz, 8H), 7.70 (d, J = 8.4 Hz, 8H), 7.66 (d, J = 8.4 Hz, 8H), 7.59 (d, J = 8.4 Hz, 8H), 6.99 (s, 4H), 4.53 (t, J = 6.4 Hz, 8H), 4.02 (t, J = 6.4 Hz, 8H), 3.53-3.65 (m, 4H), 3.30 (m, 8H), 1.85-1.97 (m, 4H), 1.54-1.65 (m, 4H). **^{13}C NMR (DMSO-d$_6$, 100 MHz) δ (ppm)**: 150.0, 137.2, 136.9, 136.4, 135.6, 128.8, 127. 4, 126.7, 126.3, 125.8, 114.5, 67.5, 65.4, 32.77. **HRMS** (MALDI-TOF) m/z: **$[M]^+$** Calcd for $C_{76}H_{72}O_{12}$ 1176.5018; Found 1176.5041. The characterization matches that previously reported.[4]

Synthesis of PnO-[10]CPP ((10^4*R*, *E*)-2^2, 2^5, 7^2, 7^5-tetra(oxypropanal)-1, 2, 3, 4, 6, 7, 8(1, 4)-heptabenzena-5, 9, 10(1, 4)-tricyclohexanacyclodecaphane-5^2, 5^4, 9^1, 9^5, 10^1, 10^5-hexaene)

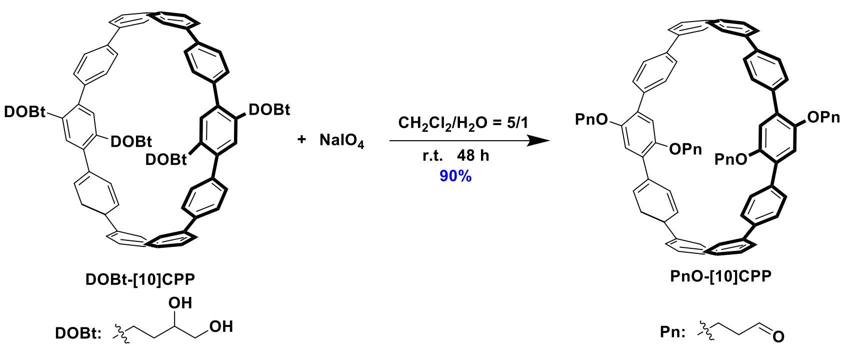

The synthesis of **PnO-[10]CPP** follows the literature procedure.[4] To a 500 mL flask were added **DOBt-[10]CPP** (588 mg, 0.5 mmol, 1 equiv.), $NaIO_4$ (2.1 g, 10 mmol, 20 equiv.) in CH_2Cl_2 (200 mL), and H_2O (40 mL). The mixture was stirred at room temperature vigorously for 48 hours. The organic layer was washed with 10% aqueous Na_2SO_3 solution (10 mL) and brine (15 mL). The organic layers were dried with anhydrous sodium sulfate and the solvent removed under reduced pressure to afford the product **PnO-[10]CPP** as a yellow solid (540 mg).

Yield: 90%. **R$_f$**: 0.66 (50% DCM/hexanes). **Mp**: 280-281 °C. **^{1}H NMR (DMSO-*d*$_6$, 400 MHz) δ (ppm)**: 9.66 (t, J = 5.6, 1.6 Hz, 4H), 7.75 (d, J = 8.8 Hz, 8H), 7.65 (d, J = 8.8 Hz, 8H), 7.63 (d, J = 8.8 Hz, 8H), 7.57 (d, J = 8.8 Hz, 8H), 7.03 (s, 4H), 4.25 (t, J = 5.6 Hz, 8H), 2.81 (td, J = 5.6, 1.6 Hz, 8H). **^{13}C NMR (DMSO-*d*$_6$, 100 MHz) δ (ppm)**: 201.8, 150.3, 137.6, 137.5, 136.8, 135.7, 129.3, 128.2, 127.2, 126.8, 126.3, 115.4, 63.1, 42.7. **HRMS** (MALDI-TOF) m/z: **[M]$^+$** Calcd: for $C_{72}H_{56}O_8$ 1048.3970; Found 1048.4024. The characterization matches that previously reported.[4]

Synthesis of OH-[10]CPP ((10^4*R*, *E*)-1, 2, 3, 4, 6, 7, 8(1, 4)-heptabenzena-5, 9, 10(1, 4)-tricyclohexanacyclodecaphane-5^2, 5^4, 9^1, 9^5, 10^1, 10^5-hexaene-2^2, 2^5, 7^2, 7^5-tetraol)

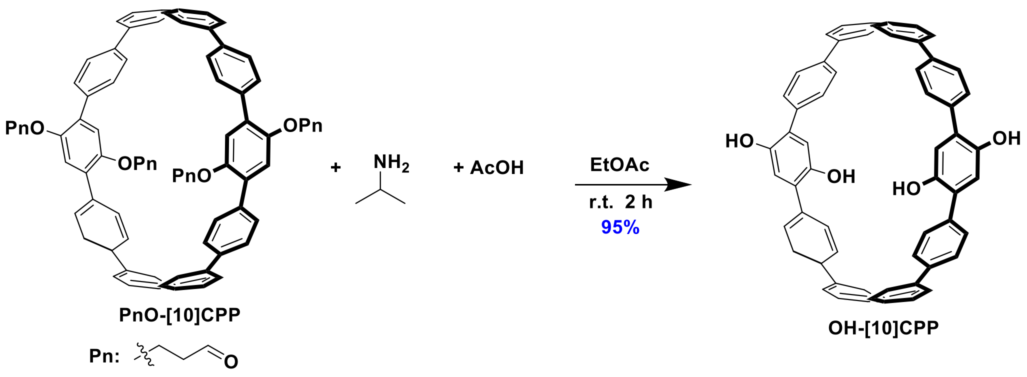

The synthesis of **OH-[10]CPP** follows the literature procedure.[4] To a 500 mL flask were added **PnO-[10]CPP** (524 mg, 0.5 mmol, 1 equiv.), isopropylamine (0.22 mL, 2.5 mmol, 5 equiv.), acetic acid (0.14 mL, 2.5 mmol, 5 equiv.), and ethyl acetate (200 mL). The mixture was bubbled with nitrogen for 30 min and stirred at room temperature for 2 hours. The reaction was quenched with 20 mL of water and extracted with ethyl acetate (50 mL×3). The combined organic layers were dried with anhydrous sodium sulfate. The organic solvent was removed under reduced pressure to afford the crude product as brown solid, which was purified by recrystallization from DCM/hexane =1/20 to afford **OH-[10]CPP** as a yellow solid (400 mg).

Yield: 95%. **R$_f$**: 0.68 (50% ethyl acetate/hexanes). **Mp**: 269-271 °C. **^{1}H NMR (CDCl$_3$, 400 MHz) δ (ppm)**: 7.62 (d, *J* = 8.6 Hz, 8H), 7.58 – 7.50 (m, 24H), 6.74 (s, 4H), 4.87 (s, 4H). **^{13}C NMR (CDCl$_3$, 100 MHz) δ (ppm)**: 147.1, 139.5, 138.4, 138.1, 135.6, 129.3, 129.1, 127.8, 127.7, 127.3, 118.1. **HRMS** (MALDI-TOF) m/z: **[M]$^+$** Calcd for $C_{60}H_{40}O_4$ 824.2921; Found 824.2923. The characterization matches that previously reported.[4]

Synthesis of OTf-[10]CPP ((10^4*R*, *E*)-1, 2, 3, 4, 6, 7, 8(1, 4)-heptabenzena-5, 9, 10(1, 4)-tricyclohexanacyclodecaphane-5^2, 5^4, 9^1, 9^5, 10^1, 10^5-hexaene-2^2, 2^5, 7^2, 7^5-tetra

(trifluoromethanesulfonate))

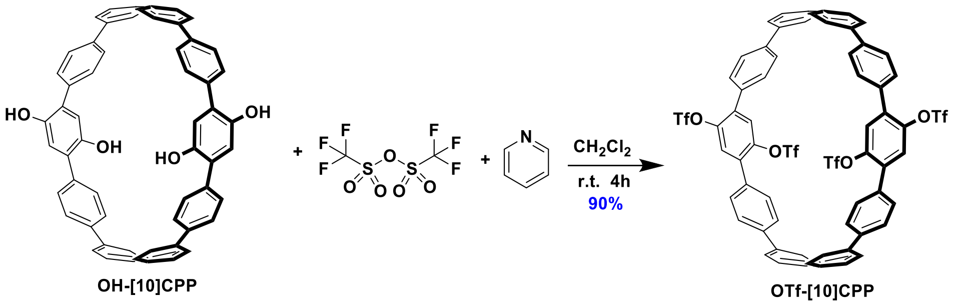

The synthesis of **OTf-[10]CPP** follows the literature procedure.[4] To a 200 mL flask were added **OH-[10]CPP** (412 mg, 0.5 mmol, 1 equiv.), triflic anhydride (1.6 mL, 10 mmol, 20 equiv.), pyridine (5 mL, 30 mmol, 60 equiv.) and the solution degassed by three cycles of vacuum-nitrogen purging. Dry DCM (50 mL) was then added. The solution was stirred at room temperature for 4 hours and then quenched with 1 mL water. The mixture was extracted with ethyl acetate (50 mL×3) and the combined organic layers were dried with anhydrous sodium sulfate. The organic solvent was removed under reduced pressure to afford the crude product, which was purified by washing with 10 mL hexane to give **OTf-[10]CPP** as a white solid (400 mg).

Yield: 90%. **R_f**: 0.60 (10% ethyl acetate/hexanes). **Mp**: 288-289 °C. **^{1}H NMR ($CDCl_3$, 400 MHz) δ (ppm)**: 7.65 (d, *J* = 8.6 Hz, 8H), 7.55 (t, *J* = 9.1 Hz, 16H), 7.47 (d, *J* = 8.5 Hz, 8H), 7.34 (s, 4H). **^{13}C NMR ($CDCl_3$, 100 MHz) δ (ppm)**: 147.1, 140.3, 138.4, 138.1, 135.6, 129.3, 129.1, 127.8, 127.7, 127.3, 118.1, 118.0. **HRMS** (MALDI-TOF) m/z: **$[M]^+$** Calcd for $C_{64}H_{36}F_{12}O_{12}S_4$ 1352.0898; Found 1352.0893. The characterization matches that previously reported.[4]

Synthesis of 4PXZPh-[10]CPP ((10^4*R*, *E*)-1, 2, 3, 4, 6, 7, 8(1, 4)-heptabenzena-5, 9, 10(1,

4)-tricyclohexanacyclodecaphane-5^2, 5^4, 9^1, 9^5, 10^1, 10^5-hexaene-2^2, 2^5, 7^2, 7^5-tetra(4-phenyl-10*H*-phenoxazine))

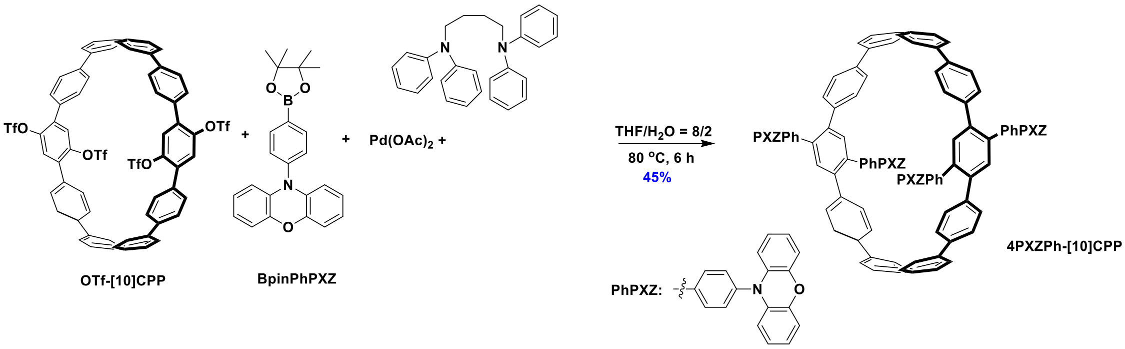

To a 50 mL Schlenk tube were added **OTf-[10]CPP** (68 mg, 0.05 mmol, 1 equiv.), BpinPhPXZ (192 mg, 0.5 mmol, 10 equiv.), palladium(II) acetate (3 mg, 0.01 mmol, 0.2 equiv.), butane-1,4-diamine (2 mg, 0.02 mmol, 0.4 equiv.), potassium carbonate (200 mg, 1.5 mol, 30 equiv.), THF (8 mL), and water (2 mL). The tube as degassed by three cycles of freeze-pump-thaw. The suspension was heated to 80 °C and stirred for 6 hours in an oil bath. After cooling, saturated ammonium chloride solution (100 mL) was added followed by extraction with ethyl acetate (20 mL × 3). The combined organic layers were dried with anhydrous magnesium sulfate. The organic solvent was removed under reduced pressure. The crude product was purified by silica gel column chromatography (ethyl acetate/hexane=1/9) and 2.5H polystyrene gel permeation chromatography (chloroform as eluent) to afford **4PXZPh-[10]CPP** as a yellow solid (25 mg). **Yield**: 45%. **R$_f$**: 0.44 (10% ethyl acetate/hexanes). **Mp**: 288-289 °C. **^{1}H NMR ($CDCl_3$, 400 MHz) δ (ppm)**: 7.52 (d, *J* = 8.6 Hz, 8H), 7.47 – 7.40 (m, 12H), 7.36 (t, *J* = 8.6 Hz, 16H), 7.19 (dd, *J* = 8.4, 6.3 Hz, 14H), 6.79 – 6.60 (m, 24H), 6.04 – 5.92 (dd, *J* = 7.8, 1.5 Hz, 8H). **^{13}C NMR ($CDCl_3$, 100 MHz) δ (ppm)**: 154.2, 144.6, 140.9, 140.2, 139.6, 138.9, 138.0, 137.9, 134.4, 134.1, 133.9, 132.8, 131.9, 128.9, 128.6, 126.8, 126.2, 123.3, 122.4, 121.6, 117.1, 115.6, 113.2, 103.2. **HRMS** (MALDI-TOF) m/z: **[M]$^+$** Calcd for $C_{132}H_{84}N_4O_4$ 1789.6526; Found 1789.6573.

Elemental analysis: Calced for $C_{132}H_{84}N_4O_4$: C, 88.57; H, 4.73; N, 3.13. **Found** C, 88.66; H, 4.76; N, 3.41. **HPLC** (C18, MeOH/MeCN = 50/50, flow rate = 1.0 mL min^{-1}, I = 254 nm) t_R = 31.3 min (major), 1.6 min (minor).

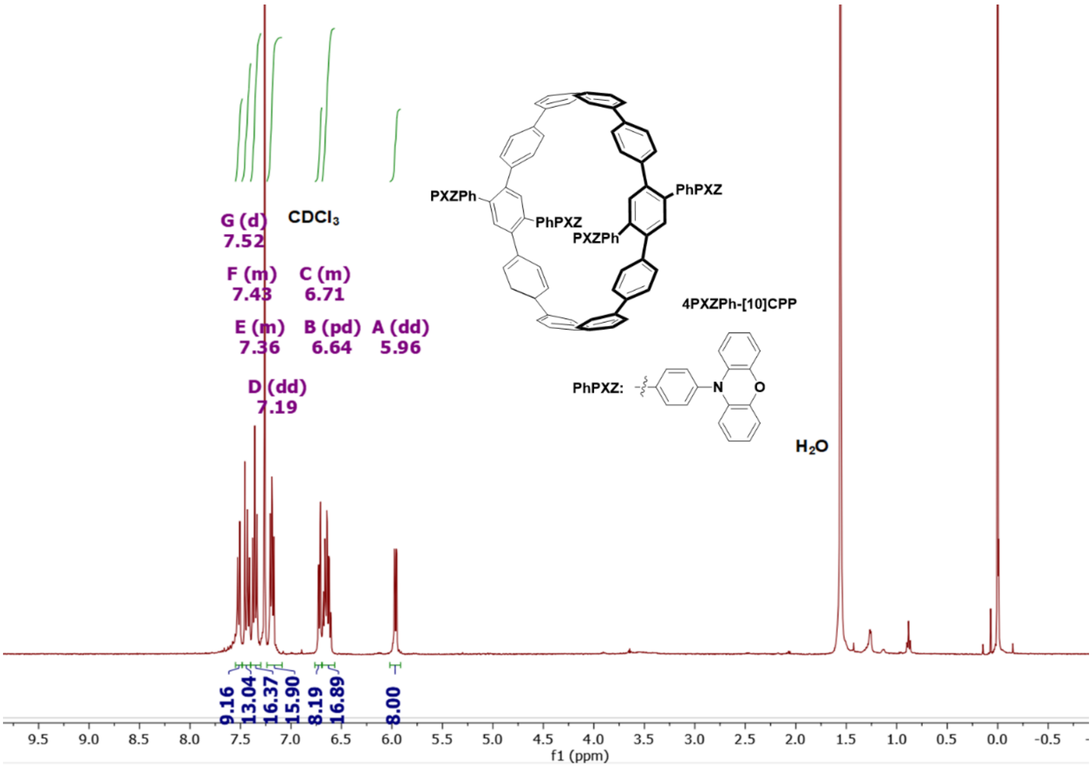

Figure **S1**. ^{1}H NMR (400 MHz) of **4PXZPh-[10]CPP** in $CDCl_3$.

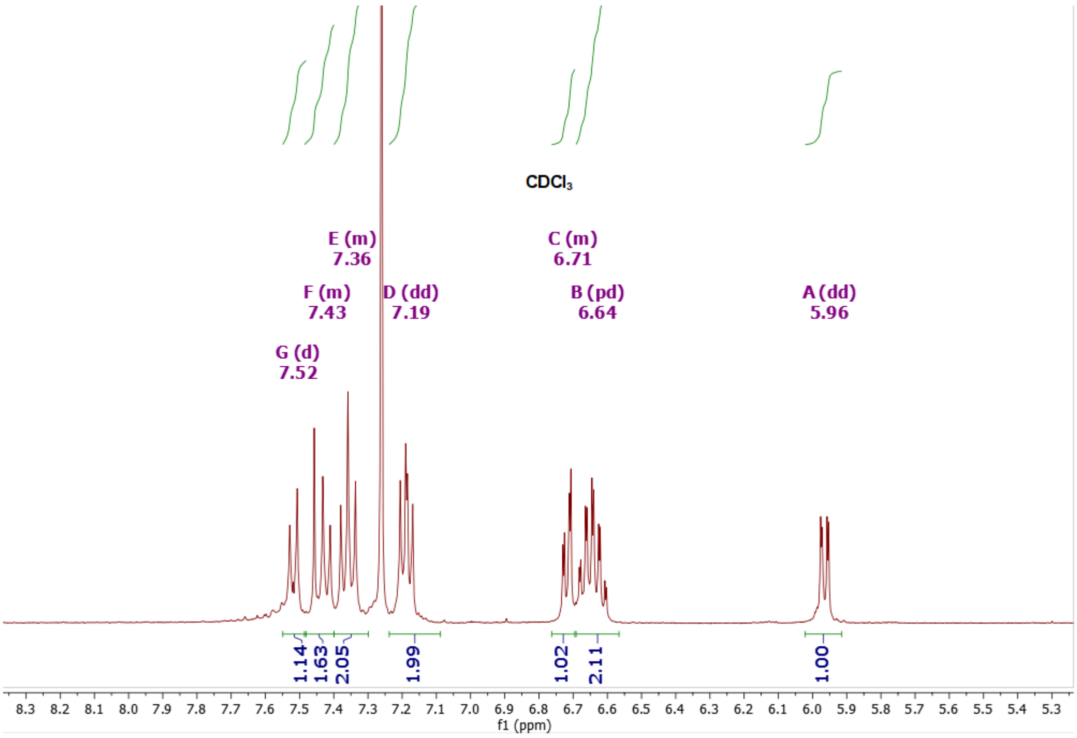

Figure **S2**. Zoomed ^{1}H NMR (400 MHz) of **4PXZPh-[10]CPP** in $CDCl_3$.

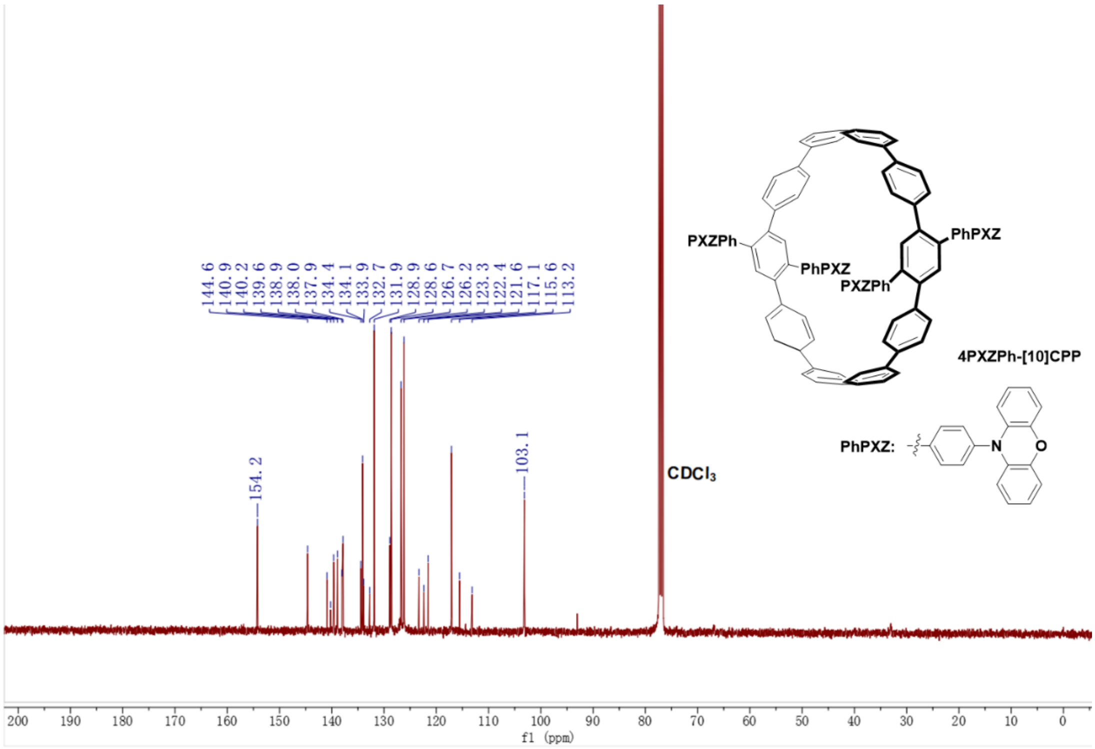

Figure **S3**. ^{13}C NMR (100 MHz) of **4PXZPh-[10]CPP** in $CDCl_3$.

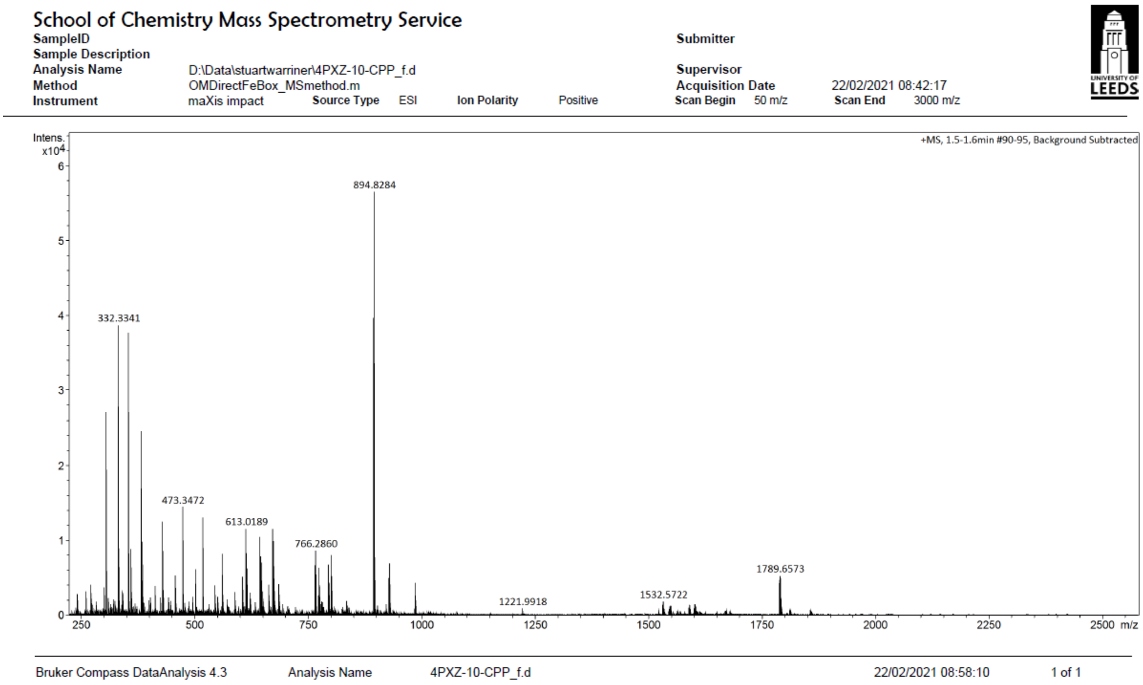

Figure **S4**. HRMS of **4PXZPh-[10]CPP**.

Elemental Analysis Service Request Form

Researcher name Dongyang Chen

Researcher email dc217@st-andrews.ac.uk

NOTE: Please submit ca. 10 mg of sample

**Sample reference number**	dc-III 09
**Name of Compound**	4PXZ-[10]CPP
**Molecular formula**	C132H84N4O4
**Stability**	stable
**Hazards**	low hazard
**Other Remarks**	

Analysis type:

Single ☐ **Duplicate**√☐ **Triplicate**☐

Analysis Result:

Element	Expected %	Found (1)	Found (2)	Found (3)
Carbon	88.57	88.27	89.01	
Hydrogen	4.73	4.66	4.89	
Nitrogen	3.13	3.58	3.27	
Oxygen				

Authorising Signature:

**Date completed**	21.02.22
**Signature**	J-P L
**comments**	

Figure **S5**. Elemental analysis of **4PXZPh-[10]CPP**.

SHIMADZU LabSolutions

Analysis Report

<Sample Information>

Sample Name	: dc-4PXZ10CPP		
Sample ID	: 4PXZ10CPP		
Data Filename	: 20042018_dc-C7CzMeOH50_dc-C7Cz_001.lcd		
Method Filename	: AcN (50) MeOH (50).lcm		
Batch Filename	: 4PXZ10CPP		
Vial #	: 1-31	Sample Type	: Unknown
Injection Volume	: 100 uL		
Date Acquired	: 19/04/2021 19:07:06	Acquired by	: ezc-7
Date Processed	: 19/04/2021 20:07:09	Processed by	: ezc-7

<Chromatogram>

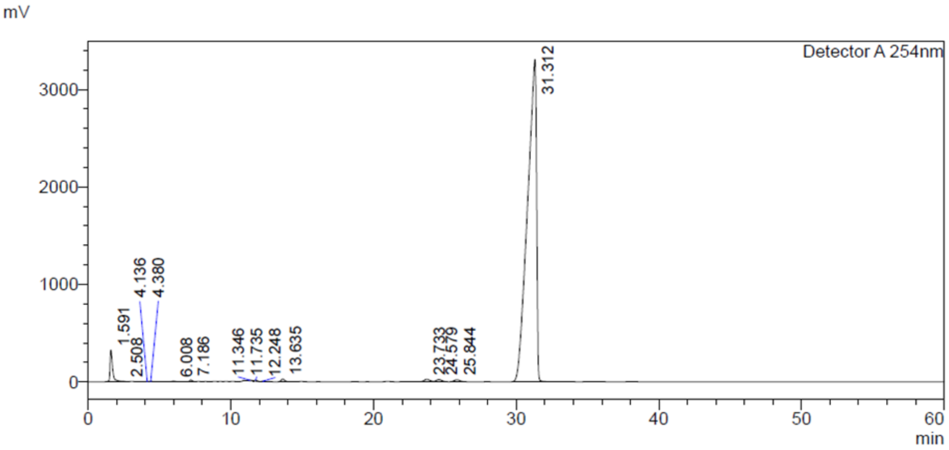

<Peak Table>

Detector A 254nm

Peak#	Ret. Time	Area	Height	Conc.	Unit	Mark	Name
1	1.591	3994745	326001	2.644		S	
2	2.508	5700	819	0.004		T	
3	4.136	25224	3011	0.017			
4	4.380	36000	4560	0.024		V	
5	6.008	69788	4763	0.046			
6	7.186	190533	17619	0.126			
7	11.346	111424	6706	0.074			
8	11.735	141195	10160	0.093		V	
9	12.248	62837	4701	0.042			
10	13.635	424742	27072	0.281			
11	23.733	627967	24711	0.416			
12	24.579	573290	22472	0.380		V	
13	25.844	526043	20035	0.348			
14	31.312	144274052	3307451	96.506			
Total		151063539	3780080				

Figure **S6**. HPLC report of **4PXZPh-[10]CPP**.

Theoretical Calculations

Ground state optimizations were carried out using Density Functional Theory (DFT) employing the PBE0[5] functional with the Pople[6] 6-31G (d,p) basis set in vacuum, followed by frequency calculations to ensure that an energy minimum were reached. Excited-state calculations were

performed employing the Tamm-Dancoff approximation (TDA)[7,8] to Time-Dependent DFT (TD-DFT) using the same functional and basis set for ground state geometry optimization. Gaussian09[9] software was employed for the calculations.[10]

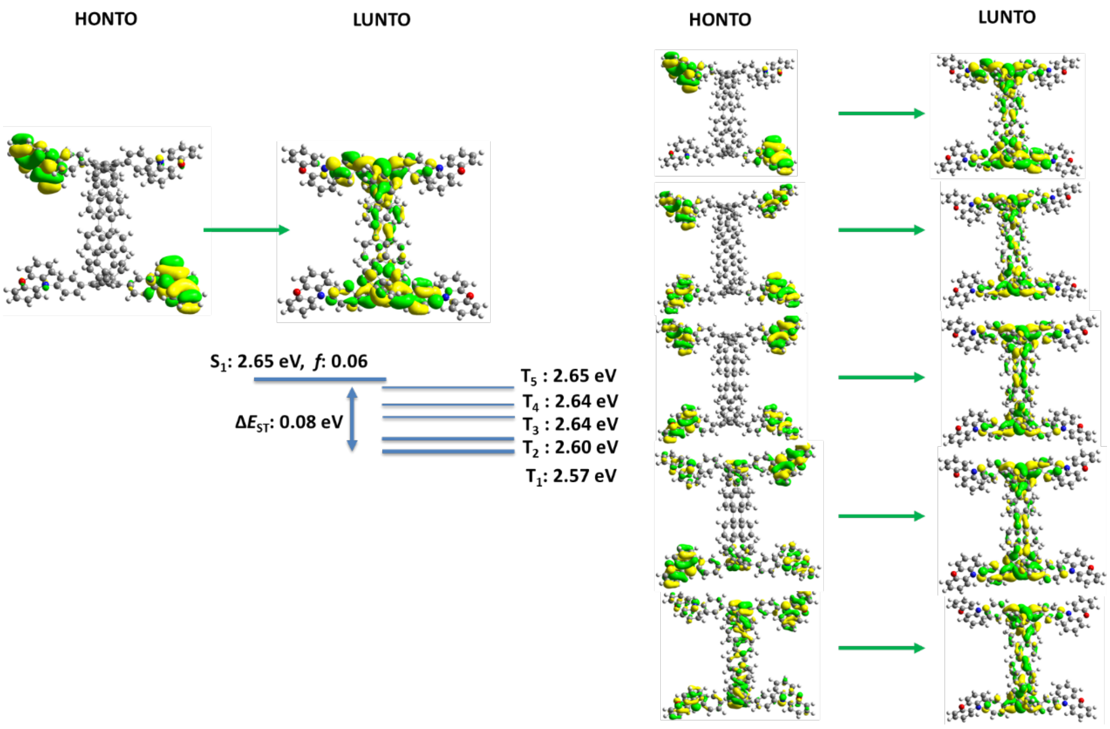

Figure **S7**. Natural transition orbitals analysis of **4PXZPh-[10]CPP** (isovalue = 0.02).

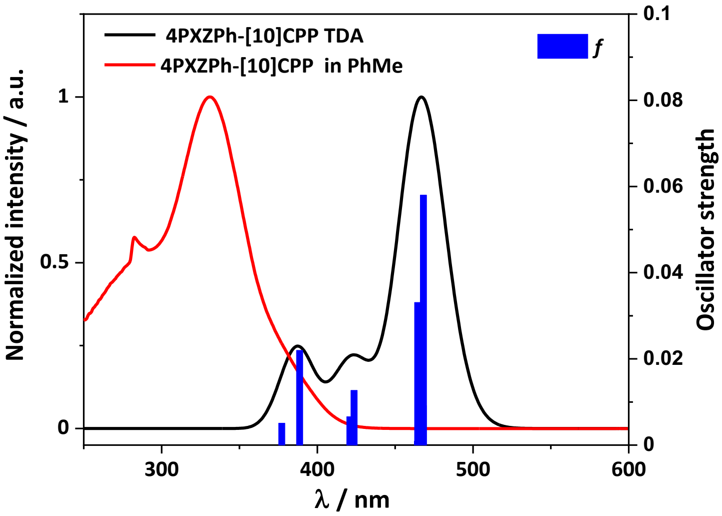

Figure **S8**. Calculated and experimental (in toluene) UV-vis spectra and oscillator strength (*f*) for **4PXZPh-[10]CPP**.

Electrochemistry measurements

Cyclic Voltammetry (CV) analysis was performed on an Electrochemical Analyzer potentiostat model 620E from CH Instruments at a sweep rate of 100 mV/s, starting potential of 0 V and cathodic direction. Differential pulse voltammetry (DPV) was conducted with an increment potential of 0.004 V and a pulse amplitude, width, and period of 50 mV, 0.05, and 0.5 s, respectively. Samples were prepared as dichloromethane (DCM) solutions, which were degassed by sparging with DCM-saturated nitrogen gas for 20 minutes prior to measurements. All measurements were performed at room temperature (~21 °C) and using 0.1 M DCM solution of tetra-*n*-butylammonium hexafluorophosphate ([$^{n}Bu_4N]PF_6$]) and 0.02 M DCM solution of **4PXZPh-[10]CPP**. A Ag/Ag^+ electrode was used as the reference electrode while a glassy carbon electrode (disc shaped with area = 0.071 cm^2) was used as and working electrode, and a platinum wire was used as the counter electrode, respectively. The redox potentials are reported relative to a saturated calomel electrode (SCE) with a ferrocenium/ferrocene (Fc/Fc^+) redox couple as the internal standard (0.46 V vs SCE).[11]

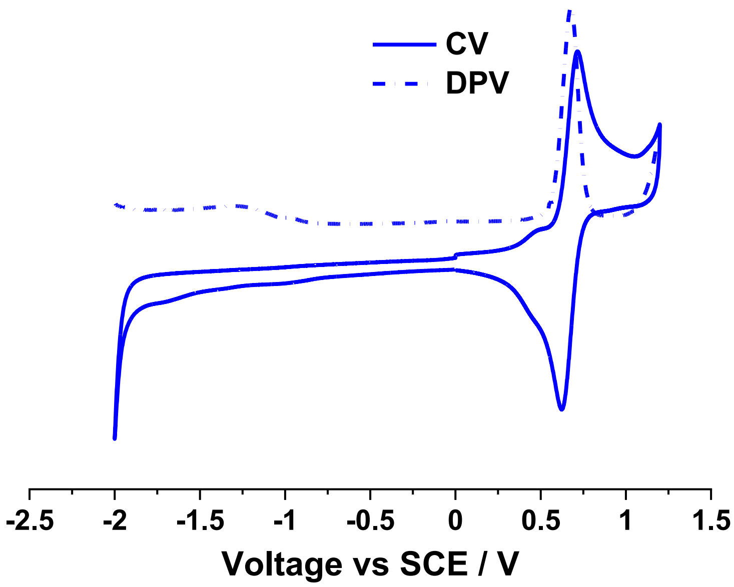

Figure **S9**. CV and DPV curves of **4PXZPh-[10]CPP** in DCM with 0.1 M *n*-Bu_4NPF_6 as the supporting electrolyte. Working electrode: glassy carbon electrode, countering electrode: platinum electrode, and referencing electrode: Ag/Ag^+. CV plotting convention: IUPAC, starting point for CV: 0 V, and scan direction: cathodic. Measured condition: scan rate 0.1 V/s, calibrated using an external standard (Fc/Fc^+=0.46 eV versus SCE).[11]

Photophysical experimental Details

Optically dilute solutions of concentrations on the order of 10^{-5} or 10^{-6} M were prepared in HPLC grade *n*-hexane, toluene, tetrahydrofuran (THF), dichloromethane (DCM), and *N,N*-dimethylformamide (DMF). Spin-coated films were produced from chloroform solutions of blends (10 wt% guest to host) at 2000 rpm for 60 seconds. Absorption spectra of solutions were recorded at room temperature on a Shimadzu UV-1800 double beam spectrophotometer using a 1 cm quartz cuvette. Molar absorptivity values were determined from at least five independent solutions at varying concentrations with absorbance ranging from 1.10×10^{-5} to 7.69×10^{-6} M.

For emission studies in solution, aerated solutions were bubbled with compressed air for 5 min prior to spectra acquisition. Degassed solutions were prepared via three freeze-pump-thaw cycles and spectra were taken using home-made Schlenk quartz cuvette. Steady-state emission spectra and time-resolved decay curves were measured using an Edinburgh Instruments F980 spectrofluorimeter. The steady-state spectra were measured using excitation at 340 nm (Xenon lamp) while the time-resolved decay curves were recorded at room temperatures with excitation at 378 nm (PDL 800-D pulsed diode laser). Photoluminescence quantum yields (Φ_{PL}) for solutions were determined using the optically dilute method[12] in which four sample solutions with absorbances of ca. 0.10, 0.075, 0.050 and 0.025 at 360 nm were used. The Beer-Lambert law was found to remain linear at the concentrations of the solutions. For each sample, linearity between absorption and emission intensity was verified through linear regression analysis with the Pearson regression factor (R^2) for the linear fit of the data set surpassing 0.9. Individual relative quantum yield values were calculated for each solution and the values reported represent the slope obtained from the linear fit of these results. The quantum yield of the sample, Φ_{PL}, can be determined by the equation $\Phi_{PL} = (\Phi_r * \frac{A_r}{A_s} * \frac{I_s}{I_r} * \frac{n_s^2}{n_r^2})$,[12] where A stands for the absorbance at the excitation wavelength (λ_{exc}: 360 nm), I is the integrated area under the corrected emission curve and n is the refractive index of the solvent with the subscripts "s" and "r" representing sample and reference respectively. Φ_r is the absolute quantum yield of the external reference quinine sulfate (Φ_r = 54.6% in 1 N H_2SO_4).[13] The experimental uncertainty in the emission quantum yields is conservatively estimated to be 10%, though we have found that statistically we can reproduce Φ_{PL}s to 3% relative error.

For emission studies in films, excitation and photoluminescence (PL) spectra were measured

by a spectrofluorometer (FluoroMax Plus, HORIBA, Japan). Φ_{PL} (or PLQY) was measured with an absolute PLQY spectrometer (C9920-02, Hamamatsu Photonics, Japan) The PLQYs for films were measured under a N_2 flow. The temperature-dependent transient PL decay measurements were conducted with a fluorescence lifetime measurement system (Quantaurus-Tau C11367-01, Hamamatsu Photonics, Japan) equipped with a cryostat (Oxford Instruments, Optistat DN2, UK).

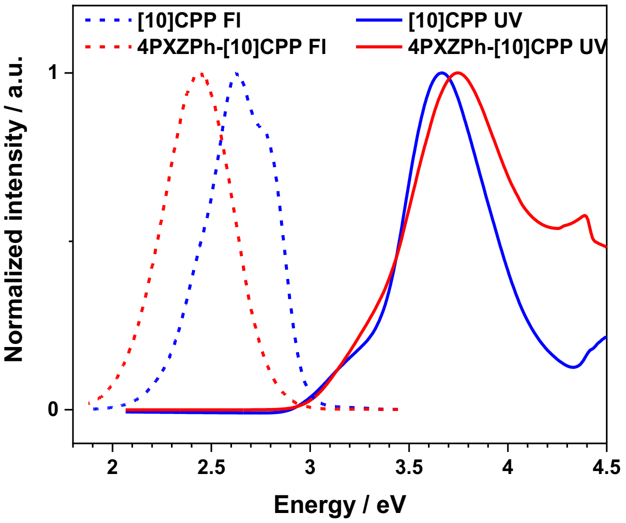

Figure **S10**. Normalized UV-vis and fluorescence spectra of **4PXZPh-[10]CPP** and **[10]CPP** in toluene (λ_{exc} = 340 nm).

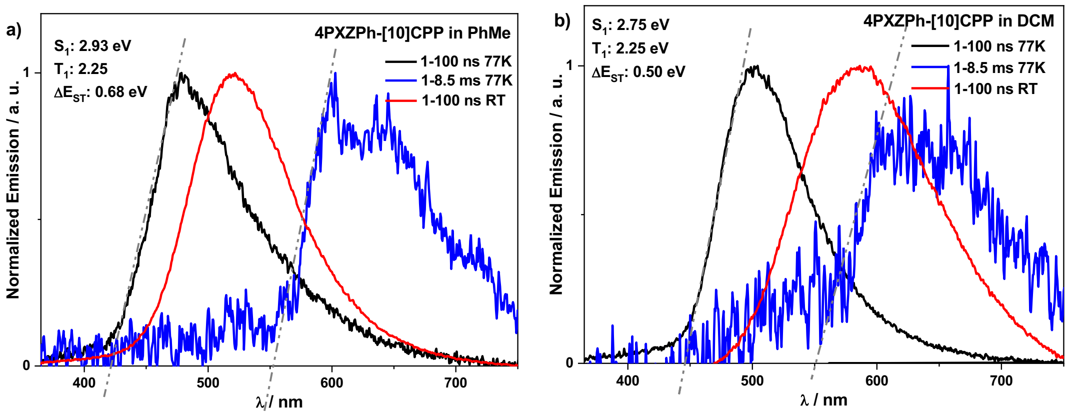

Figure **S11**. Steady-state PL at room temperature, prompt PL at 77 K (delay: 1 ns, gate: 100 ns), and phosphorescence spectra at 77 K (delay: 1 ms, gate: 8.5 ms) in a) toluene and b) DCM, λ_{exc}

= 343 nm.

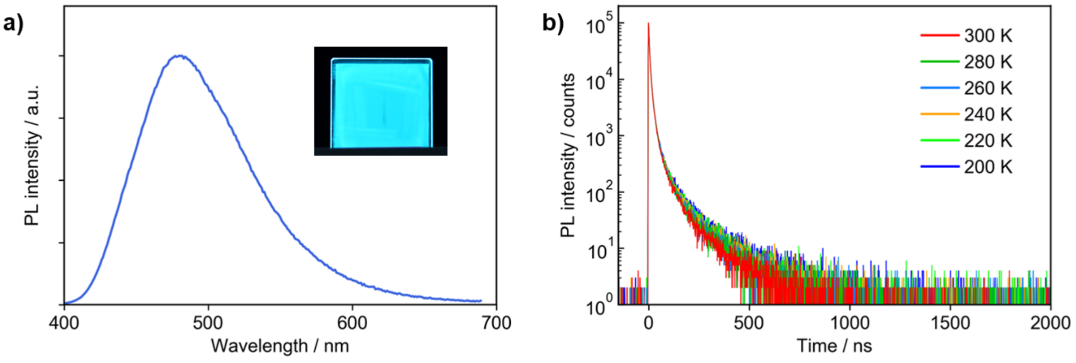

Figure **S12**. a) Emission spectra and a photo (inset); b) Temperature-dependent transient PL decay spectra of **4PXZPh-[10]CPP**/PyD2 film (λ_{exc} = 365 nm).

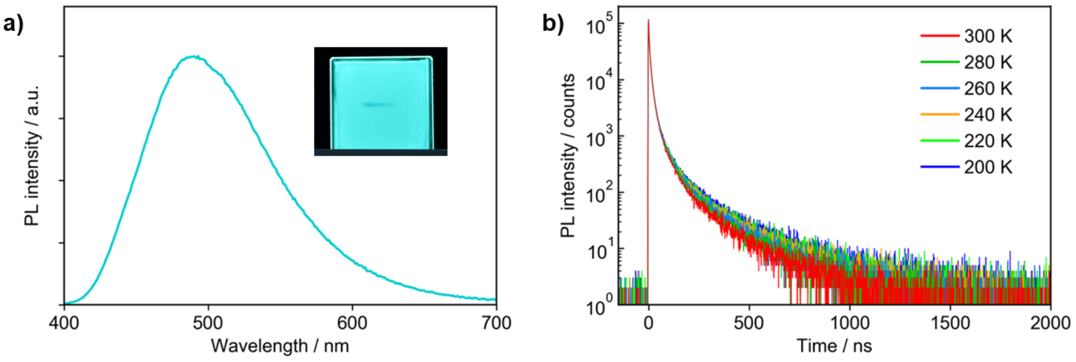

Figure **S13**. a) Emission spectra and a photo (inset); b) the temperature-dependent transient PL decay spectra of **4PXZPh-[10]CPP**/PPF film (λ_{exc} = 365 nm).

Device Fabrication and Characterization

For the 10 wt% **4PXZPh-[10]CPP**/CzSi-based OLEDs, 50 vol% PEDOT:PSS (Heraeus, CH 8000) diluted in pure water was deposited on the pre-cleaned ITO substrates by spin-coating at 500 rpm for 1 s then at 4000 rpm for 12 s, and at 500 rpm for 1 s. Subsequently, 10 mg mL^{-1}

PVK dissolved in 1,2-dichlorobenzene was spin-coated onto the film at 2000 rpm for 30 s. The film was dried at 150 °C for 10 min under air condition followed at room temperature using a N_2 flow for 5 min, following by drying at 120 ℃ for 10 min under air. The 10 wt% **4PXZPh-[10]CPP**/CzSi emissive layer was dissolved in toluene and then the solution was spin-coated at 2200 rpm for 30 s, then dried at 100 ℃ under air for 10 min, and cooled at room temperature under N_2 flow for 5 min. The PPF (5 nm), TPBi (45 nm), Liq (1 nm), and Al (80 nm) layers were vacuum deposited at ~10^{-4} Pa using a deposition apparatus (SE-4260, ALS Technology, Japan), resulting in active areas of 4 mm^2 for each pixel.

For the **4PXZPh-[10]CPP**/PyD2 based OLEDs: PEDOT:PSS (Heraeus, AI 4083) was spin-coated onto pre-cleaned ITO substrate at 500 rpm for 1 s then at 4000 rpm for 12 s, and at 500 rpm for 1 s. The film was dried at 120 °C under air for 30 min followed by under a N_2 flow for 10 min. The 10 mg mL^{-1} emitter in chloroform solution was stirred at 1000 rpm at 30 °C for 1 h before spin-coated onto the PEDOT:PSS layer at 1000 rpm for 30 s. The device was dried at 30 °C under vacuum for 30 min and cooled down under N_2 flow for 5 min. The BmPyPhB (45 nm), Liq (1 nm), and Al (80 nm) layers were vacuum deposited at ~10^{-4} Pa.

Performance of OLEDs were characterized using an integrating sphere integrated with an absolute EQE measurement system (C9920-12, Hamamatsu Photonics, Japan) equipped with a source meter (2400, Keithley, Japan). The device performances were measured in the forward direction in 200-meV steps.

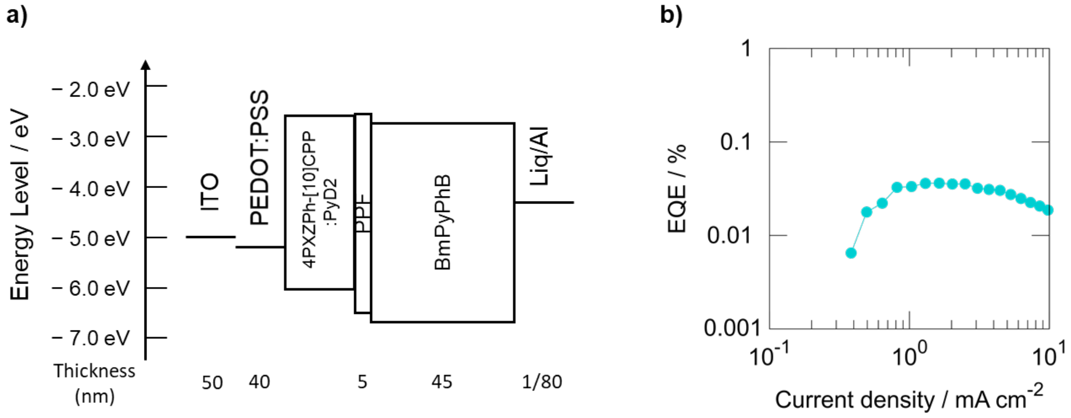

Figure **S14**. a) Device stack of **4PXZPh-[10]CPP** based OLEDs; b) EQE-current density characteristics.

NMR Spectra of Intermediates

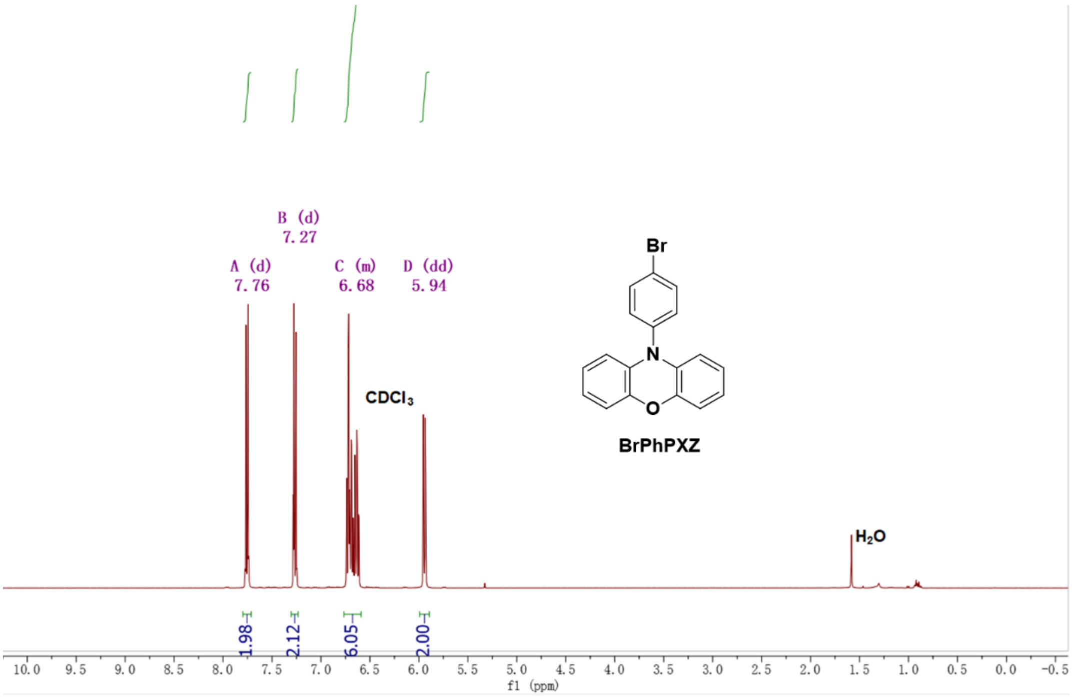

Figure **S15**. ^{1}H NMR (400 MHz) of **BrPhPXZ** in $CDCl_3$.

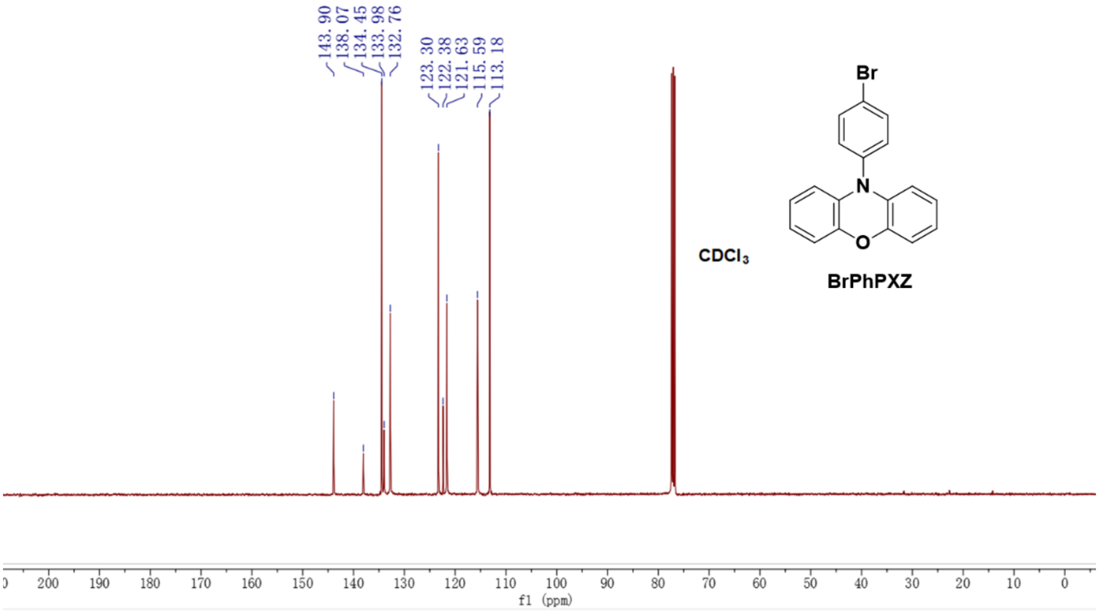

Figure **S16**. ^{13}C NMR (100 MHz) of **BrPhPXZ** in $CDCl_3$.

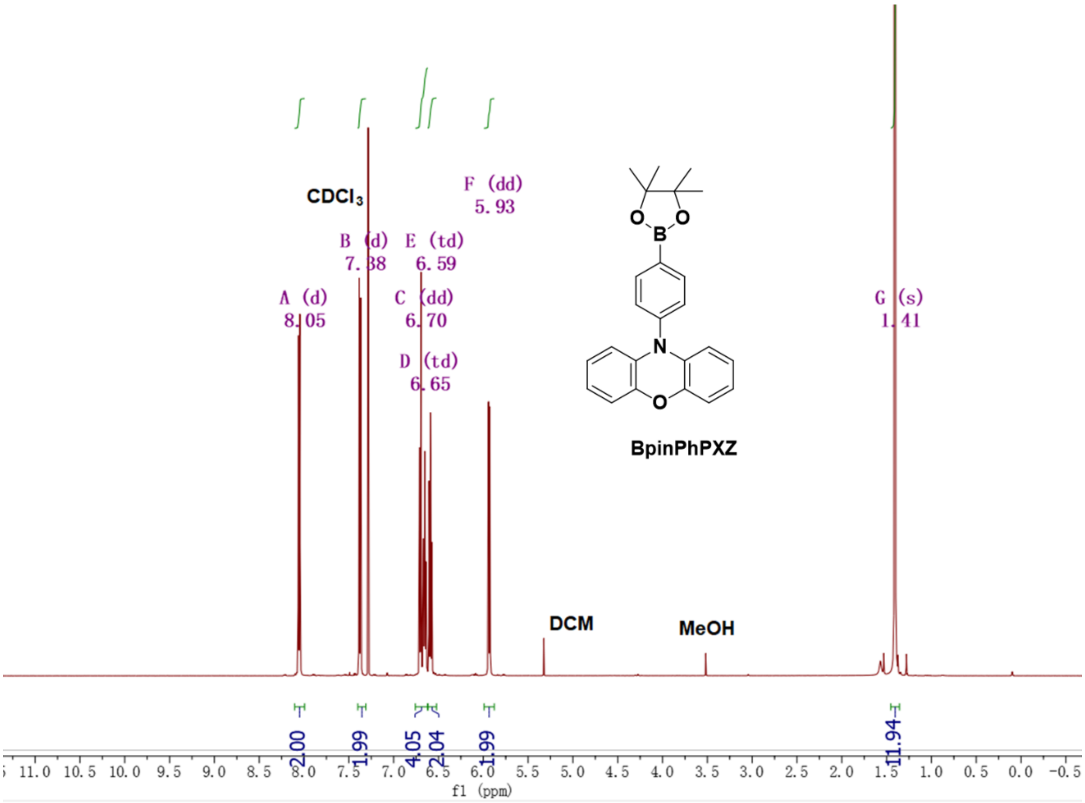

Figure **S17**. ^{1}H NMR (500 MHz) of **BpinPhPXZ** in $CDCl_3$.

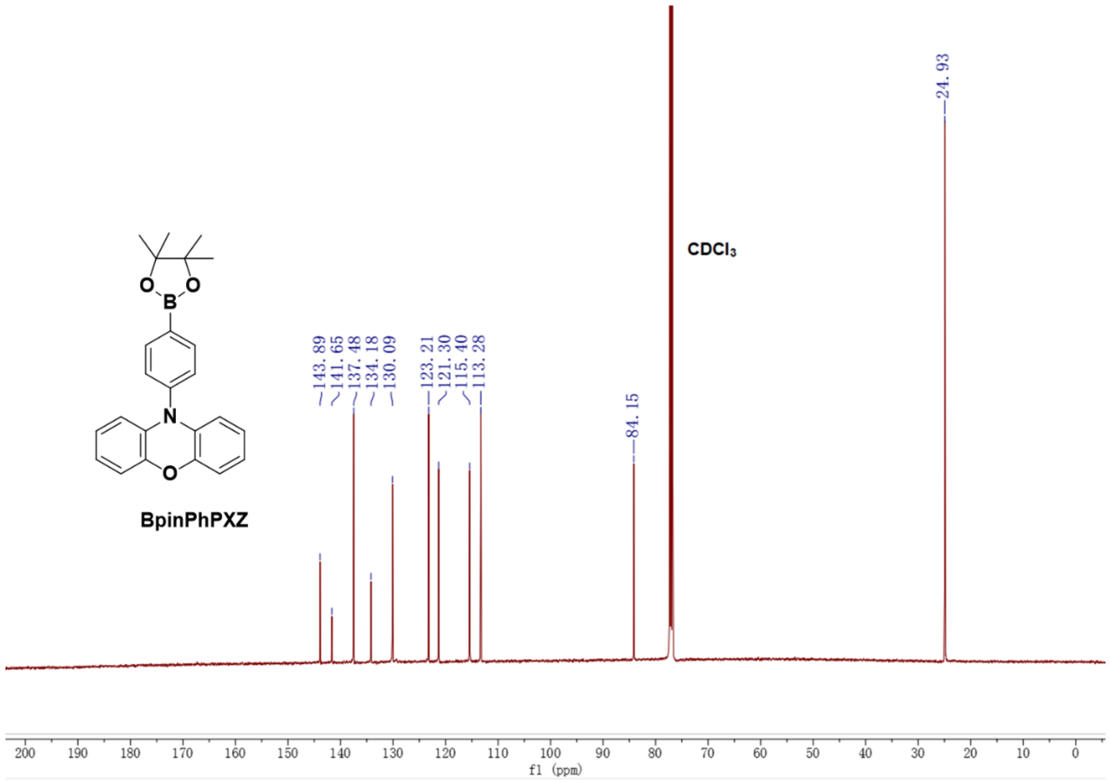

Figure **S18**. ^{13}C NMR (125 MHz) of **BpinPhPXZ** in $CDCl_3$.

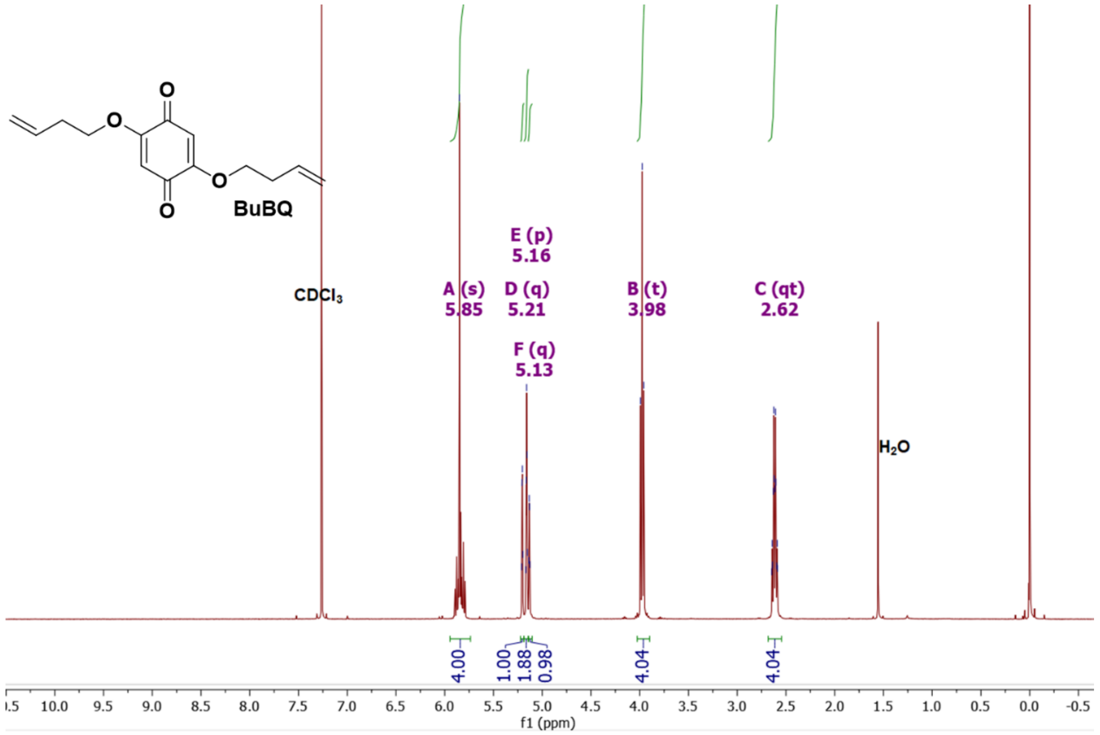

Figure **S19**. ^{1}H NMR (400 MHz) of **BuBQ** in $CDCl_3$.

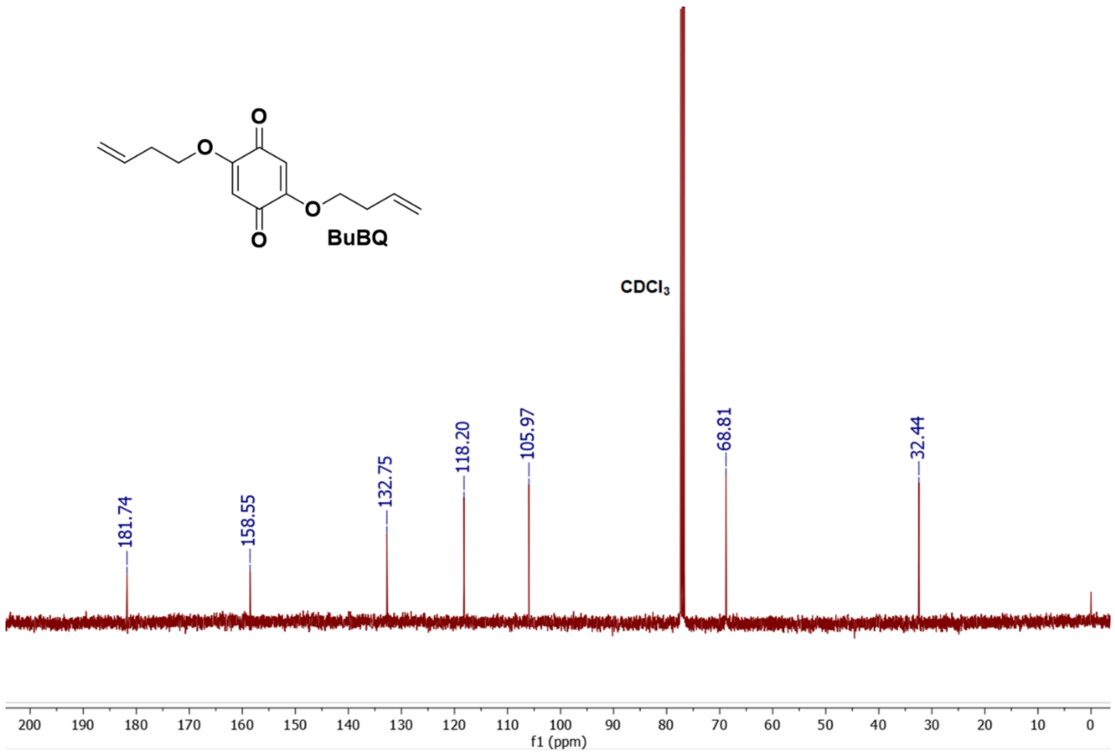

Figure **S20**. ^{13}C NMR (100 MHz) of **BuBQ** in $CDCl_3$.

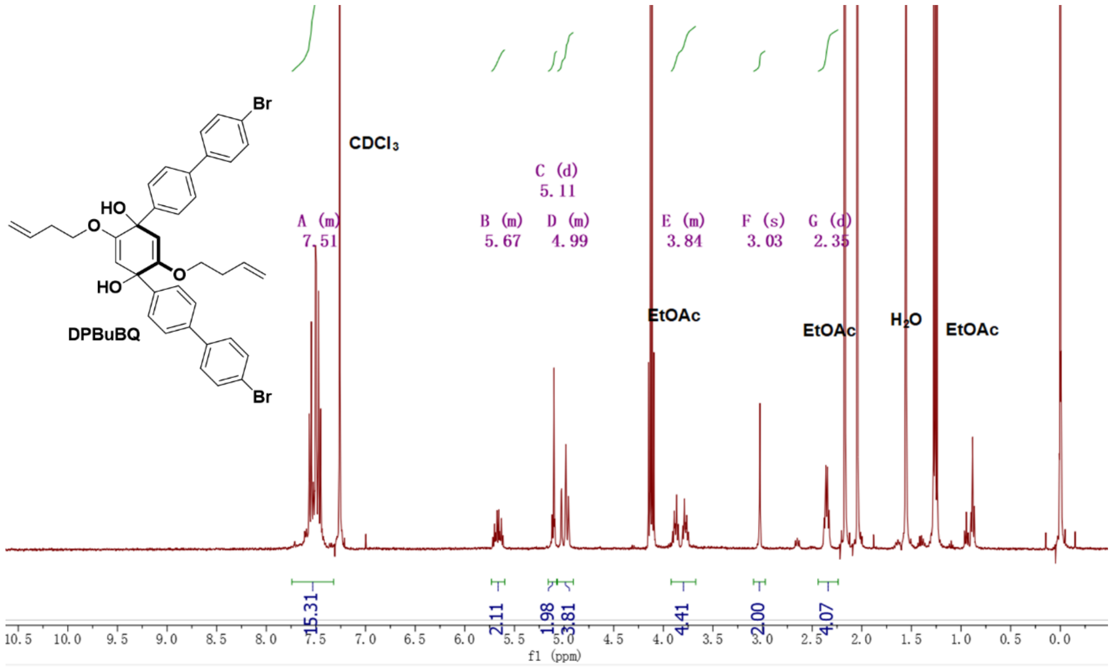

Figure **S21**. ^{1}H NMR (400 MHz) of **DPBuBQ** in $CDCl_3$.

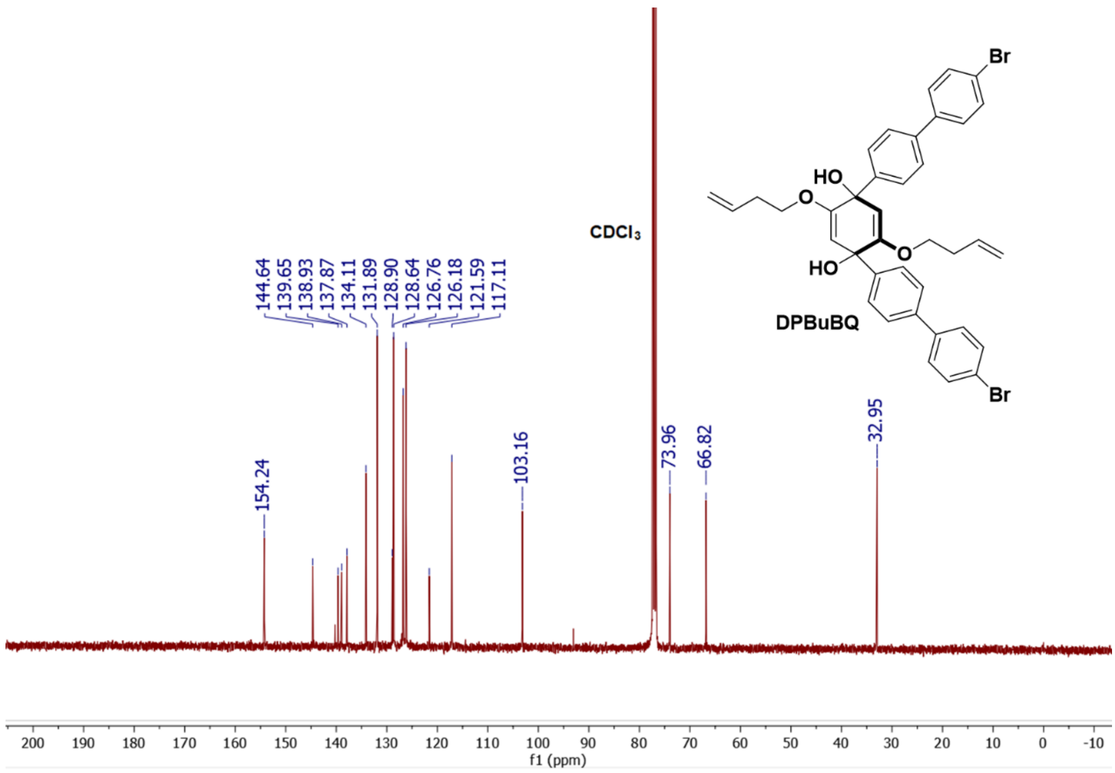

Figure **S22**. ^{13}C NMR (100 MHz) of **DPBuBQ** in $CDCl_3$.

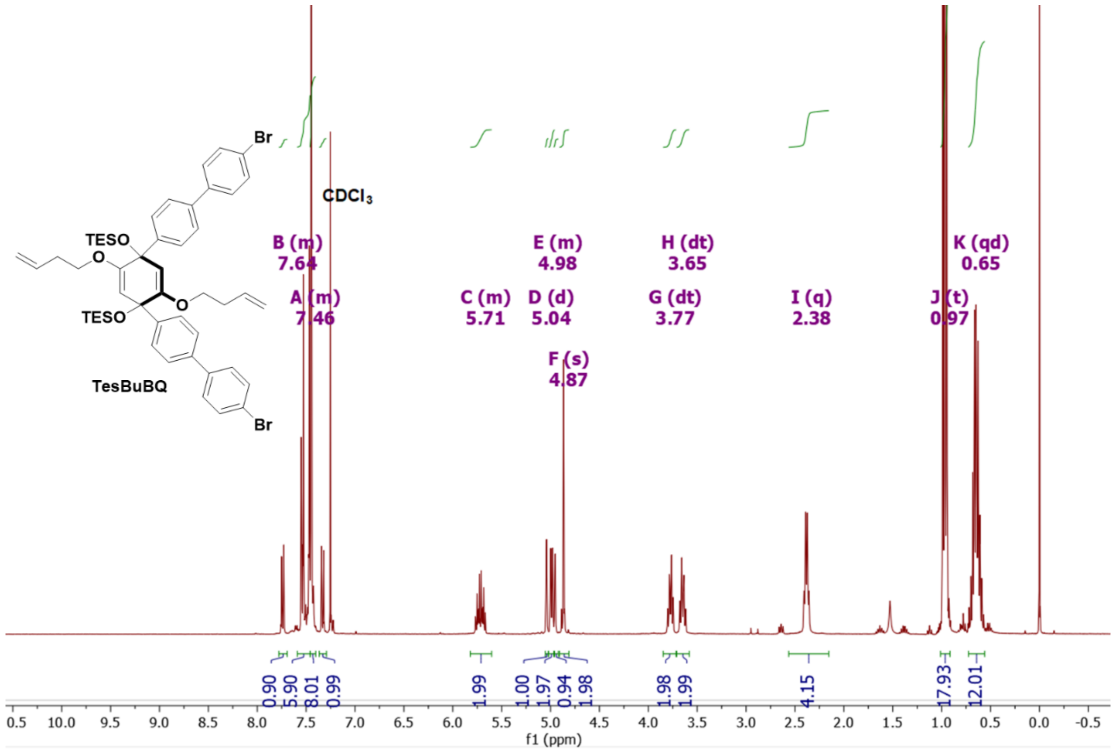

Figure **S23**. ^{1}H NMR (400 MHz) of **TesBuBQ** in $CDCl_3$.

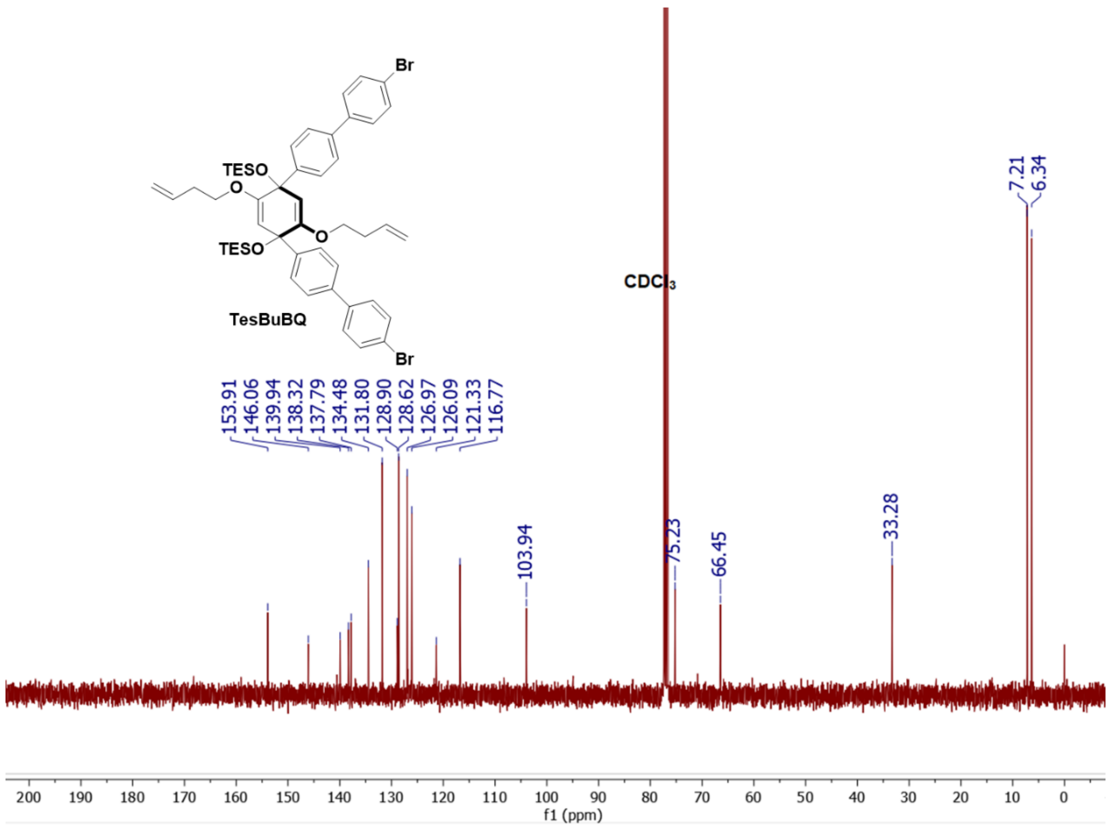

Figure **S24**. ^{13}C NMR (100 MHz) of **TesBuBQ** in $CDCl_3$.

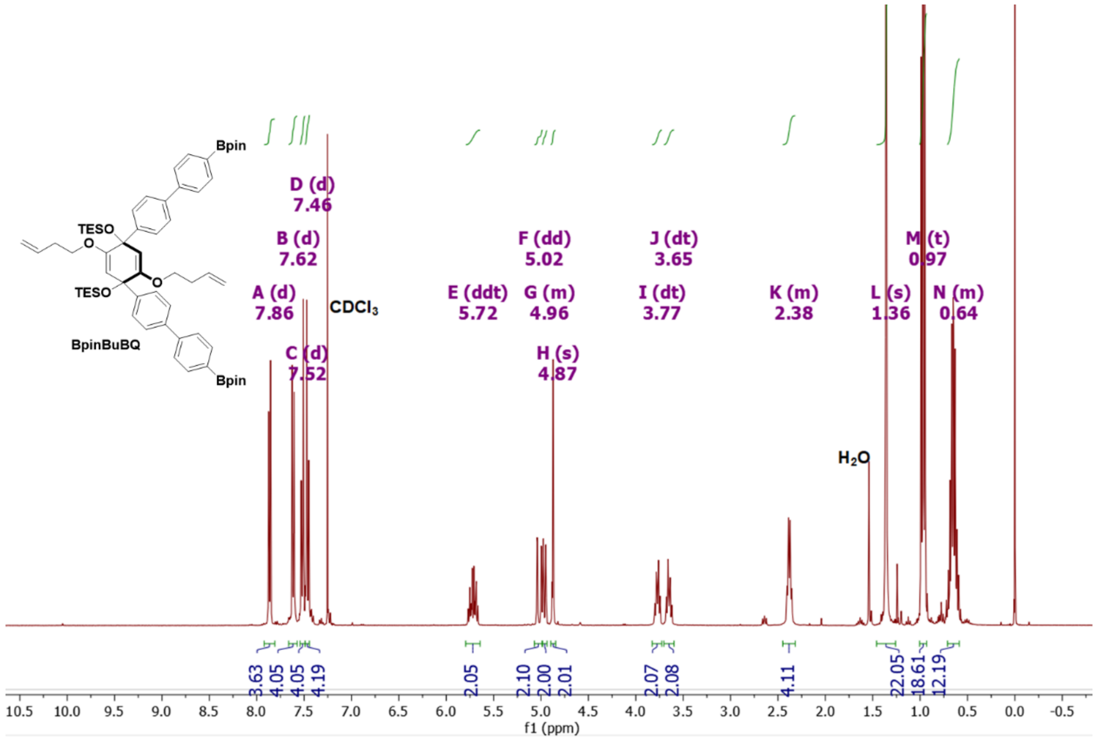

Figure **S25**. ^{1}H NMR (400 MHz) of **BpinBuBQ** in $CDCl_3$.

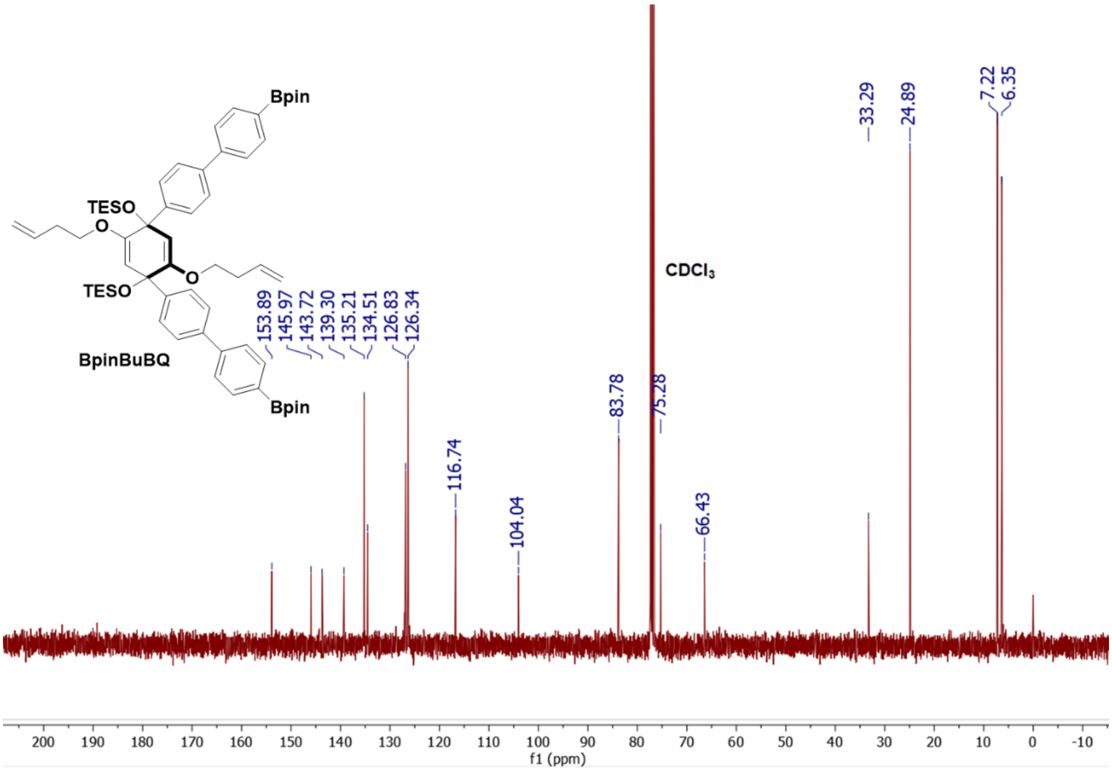

Figure **S26**. ^{13}C NMR (100 MHz) of **BpinBuBQ** in $CDCl_3$.

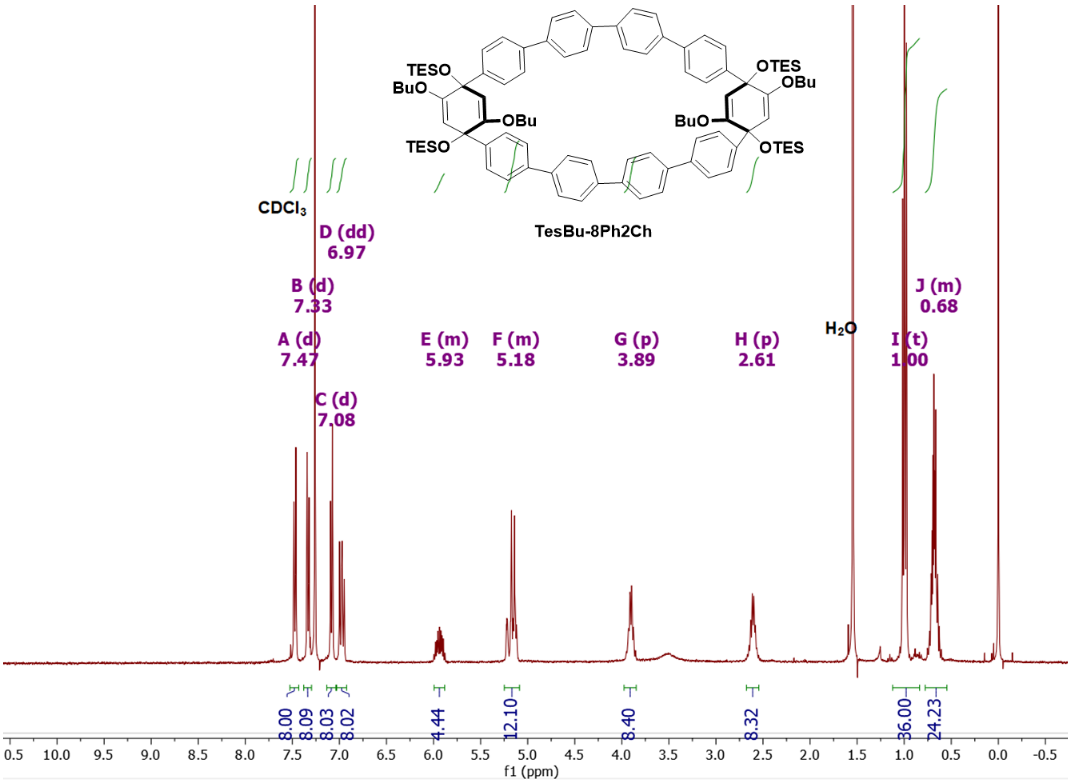

Figure **S27**. ^{1}H NMR (400 MHz) of **TesBu-8Ph2Ch** in $CDCl_3$.

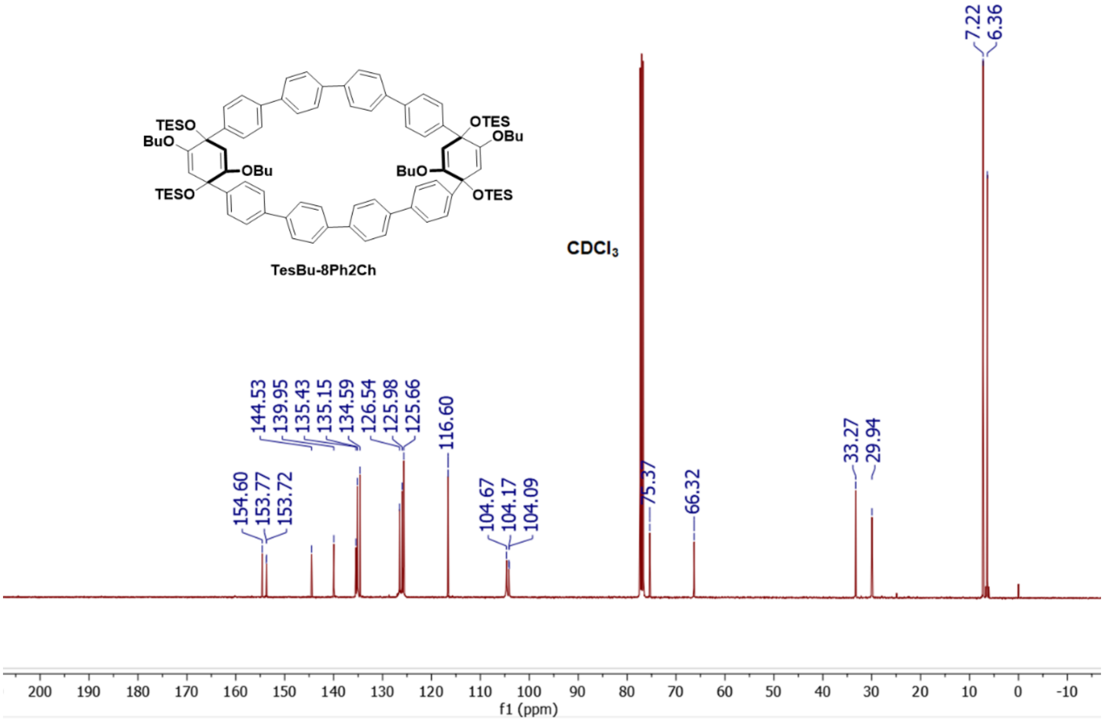

Figure **S28**. ^{13}C NMR (100 MHz) of **TesBu-8Ph2Ch** in $CDCl_3$.

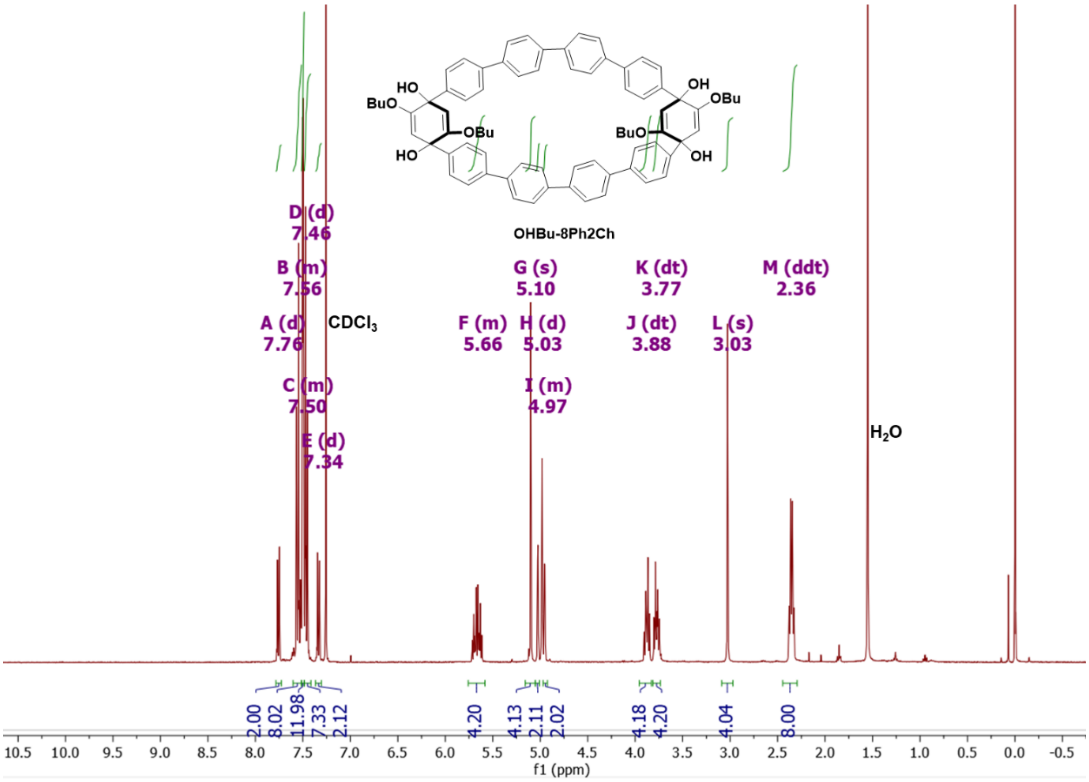

Figure **S29**. ^{1}H NMR (400 MHz) of **OHBu-8Ph2Ch** in $CDCl_3$.

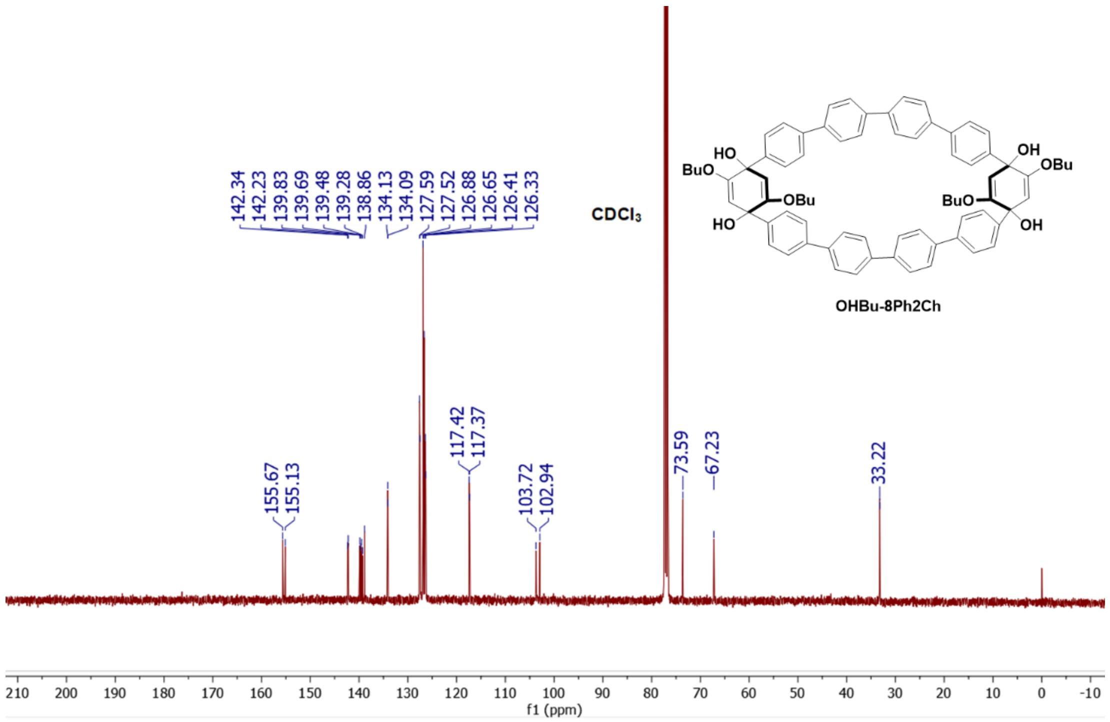

Figure **S30**. ^{13}C NMR (100 MHz) of **OHBu-8Ph2Ch** in $CDCl_3$.

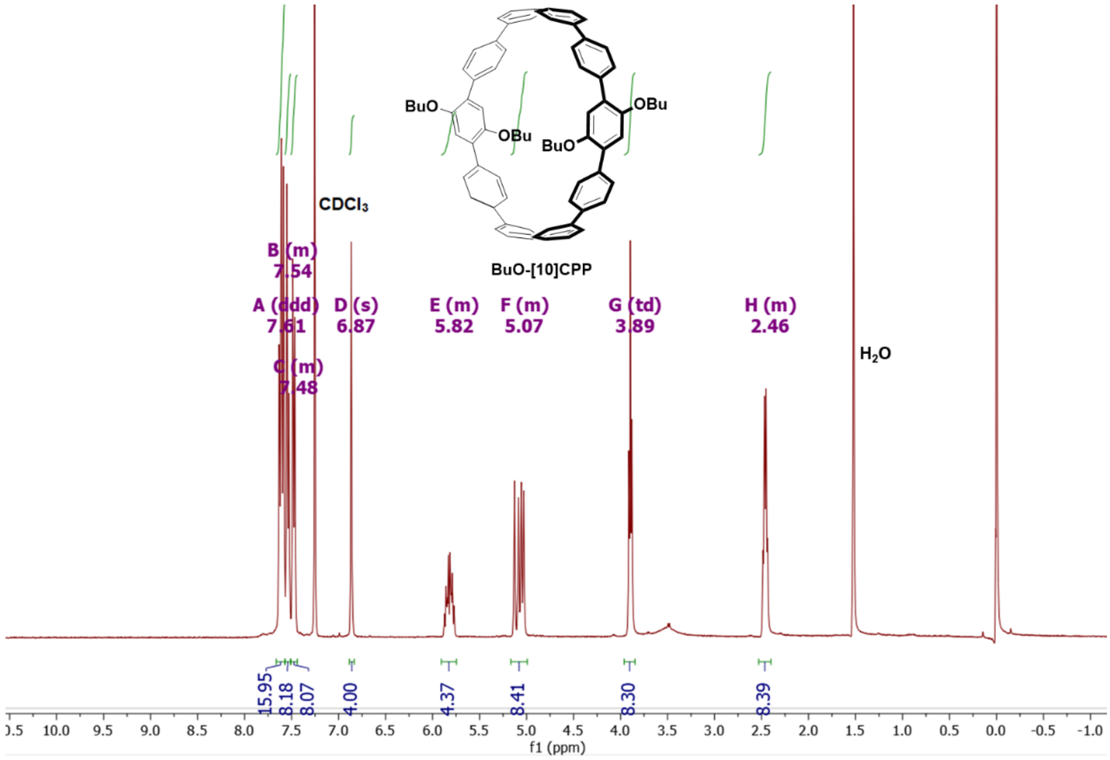

Figure **S31**. ^{1}H NMR (400 MHz) of **BuO-[10]CPP** in $CDCl_3$.

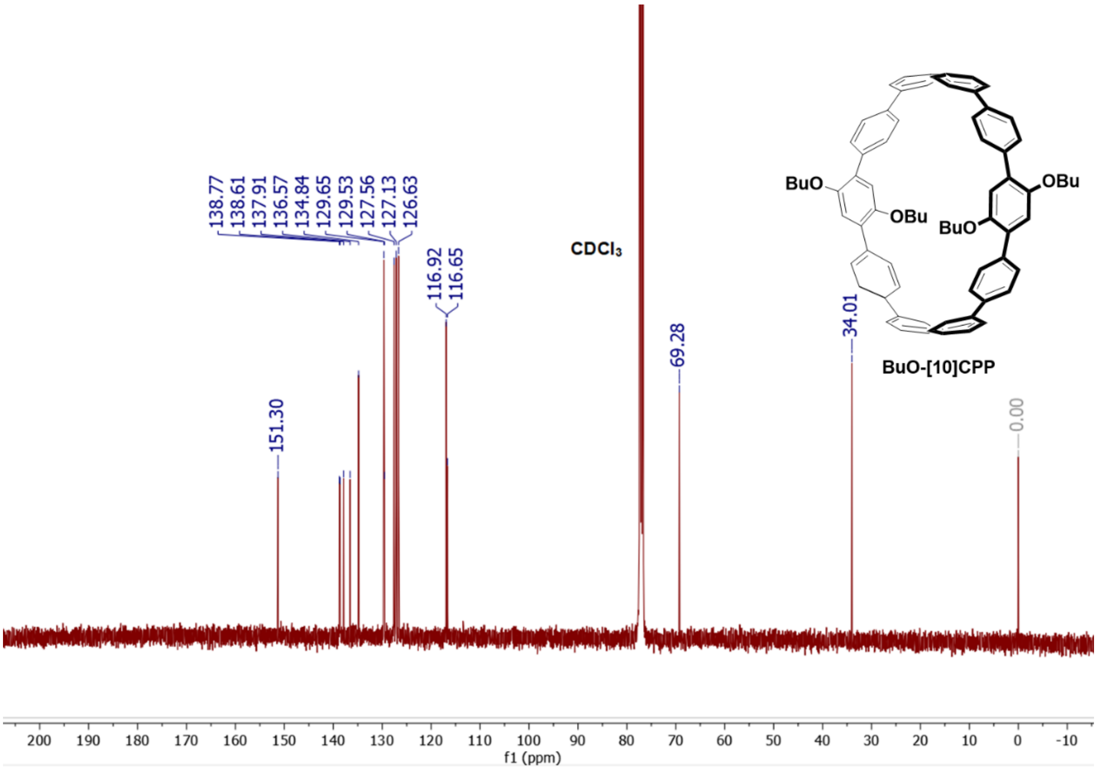

Figure **S32**. ^{13}C NMR (100 MHz) of **BuO-[10]CPP** in $CDCl_3$.

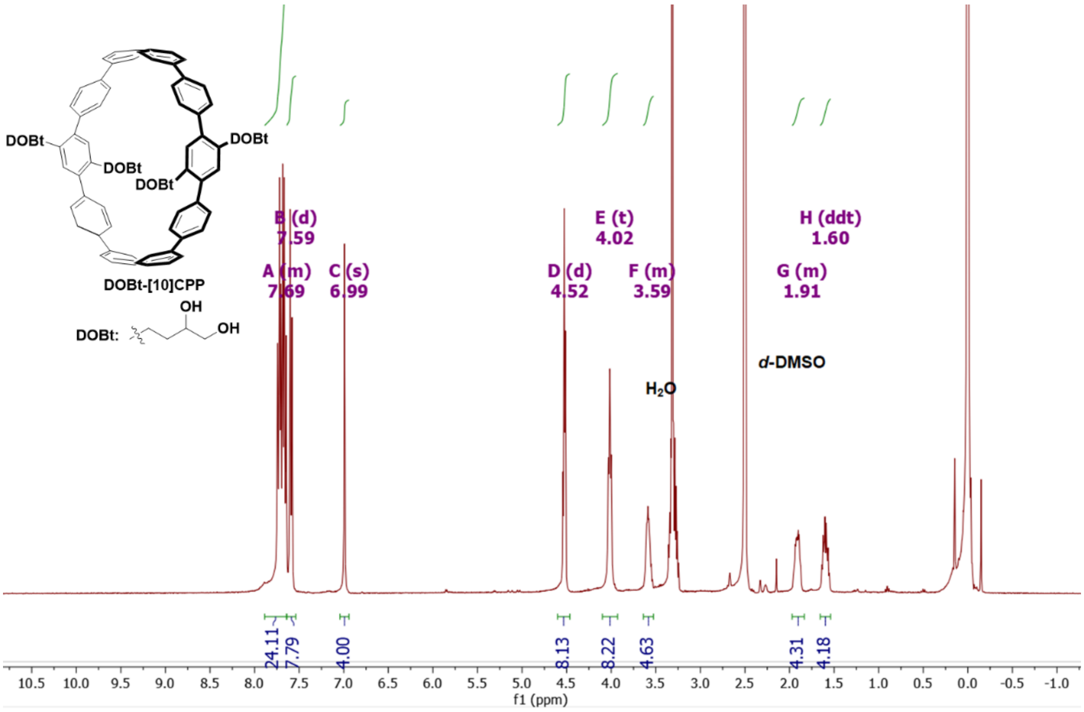

Figure **S33**. ^{1}H NMR (400 MHz) of **DOBt-[10]CPP** in *d*-DMSO.

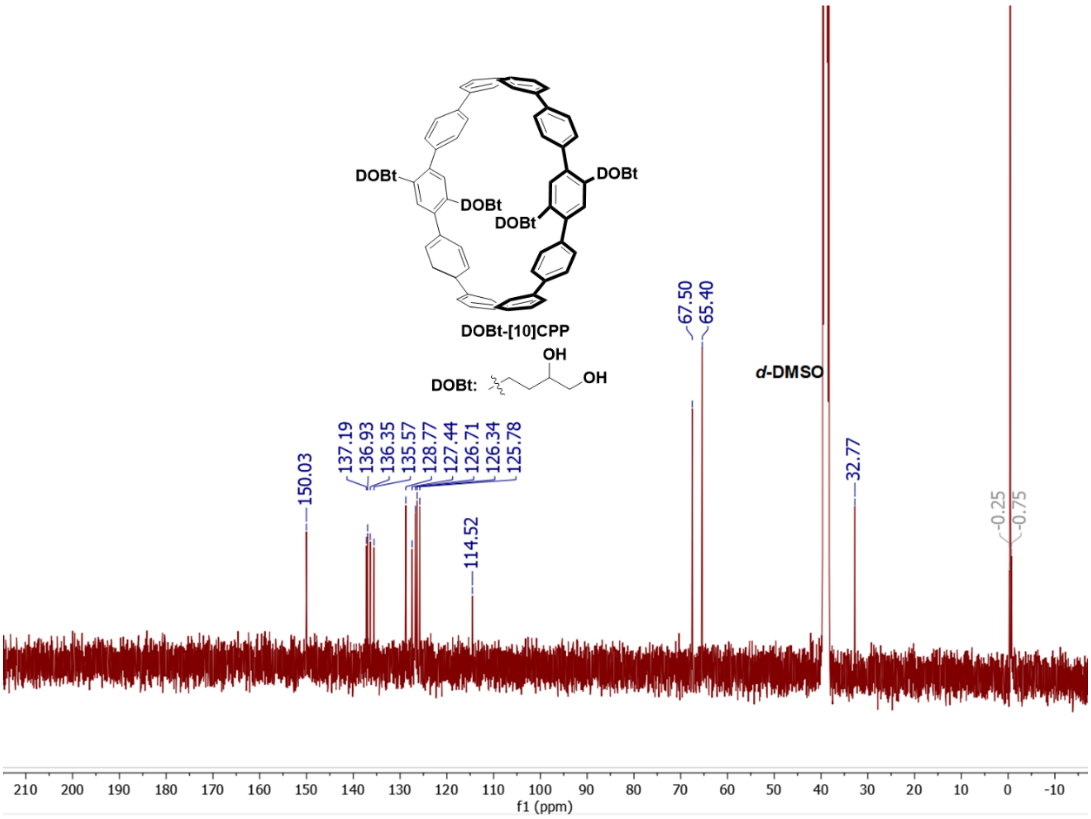

Figure **S34**. ^{13}C NMR (100 MHz) of **DOBt-[10]CPP** in *d*-DMSO.

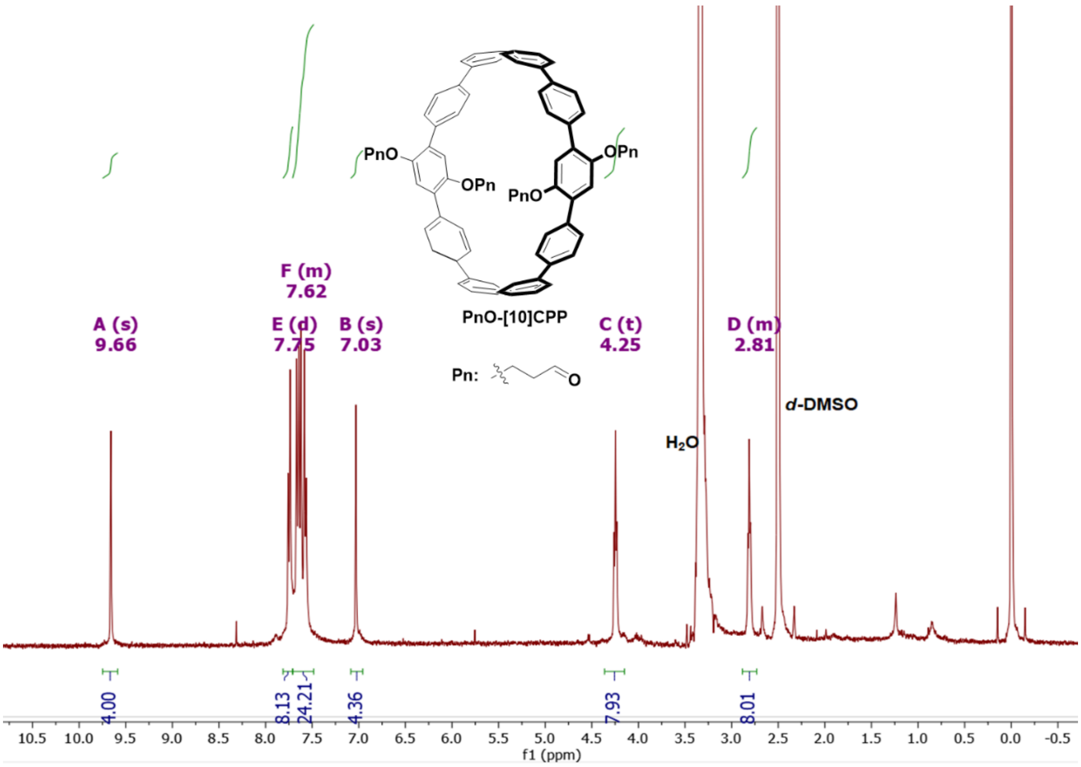

Figure **S35**. ^{1}H NMR (400 MHz) of **PnO-[10]CPP** in *d*-DMSO.

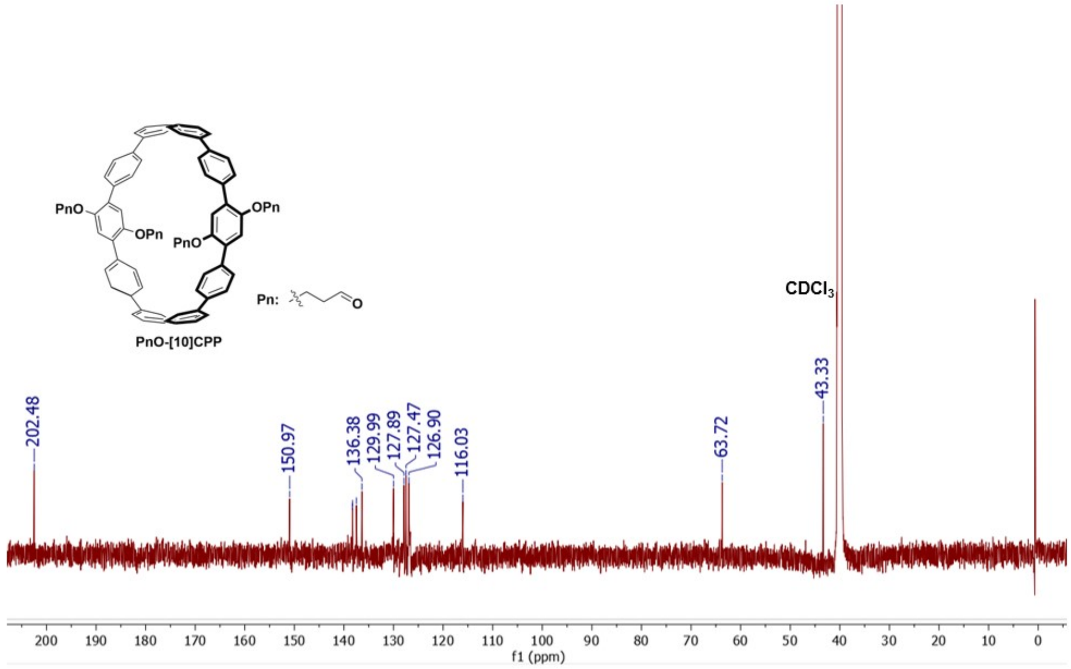

Figure **S36**. ^{13}C NMR (100 MHz) of **PnO-[10]CPP** in *d*-DMSO.

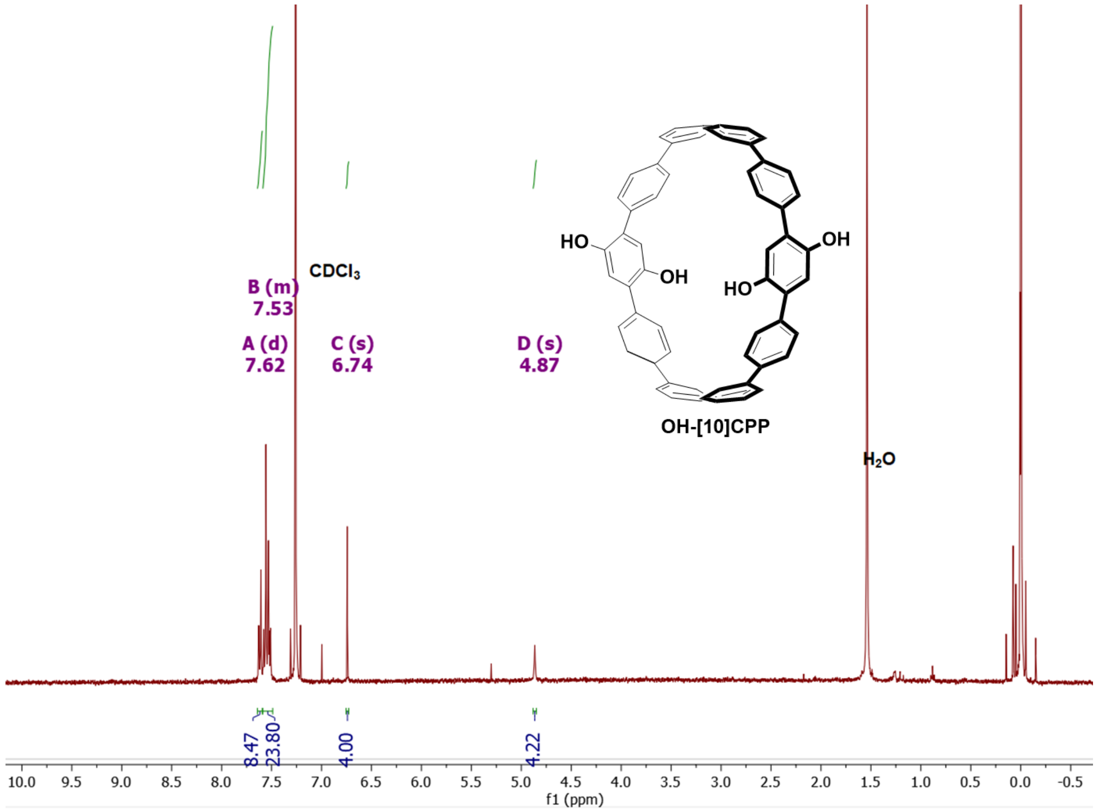

Figure **S37**. ^{1}H NMR (400 MHz) of **OH-[10]CPP** in $CDCl_3$.

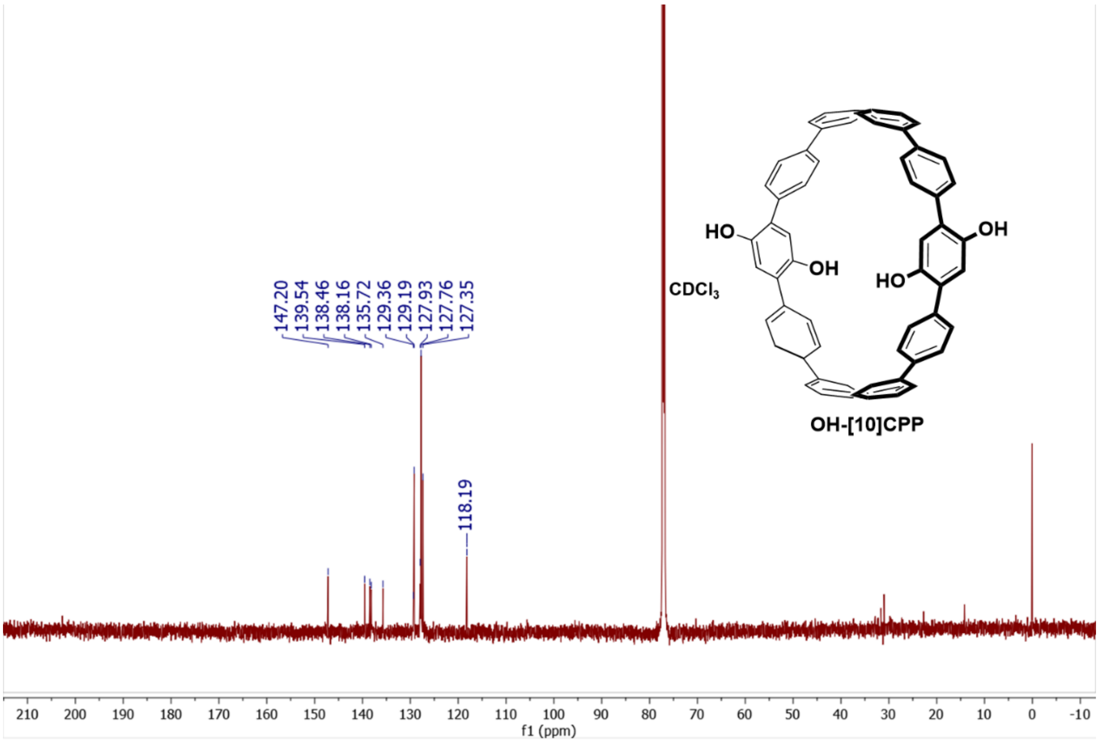

Figure **S38**. ^{13}C NMR (100 MHz) of **OH-[10]CPP** in $CDCl_3$.

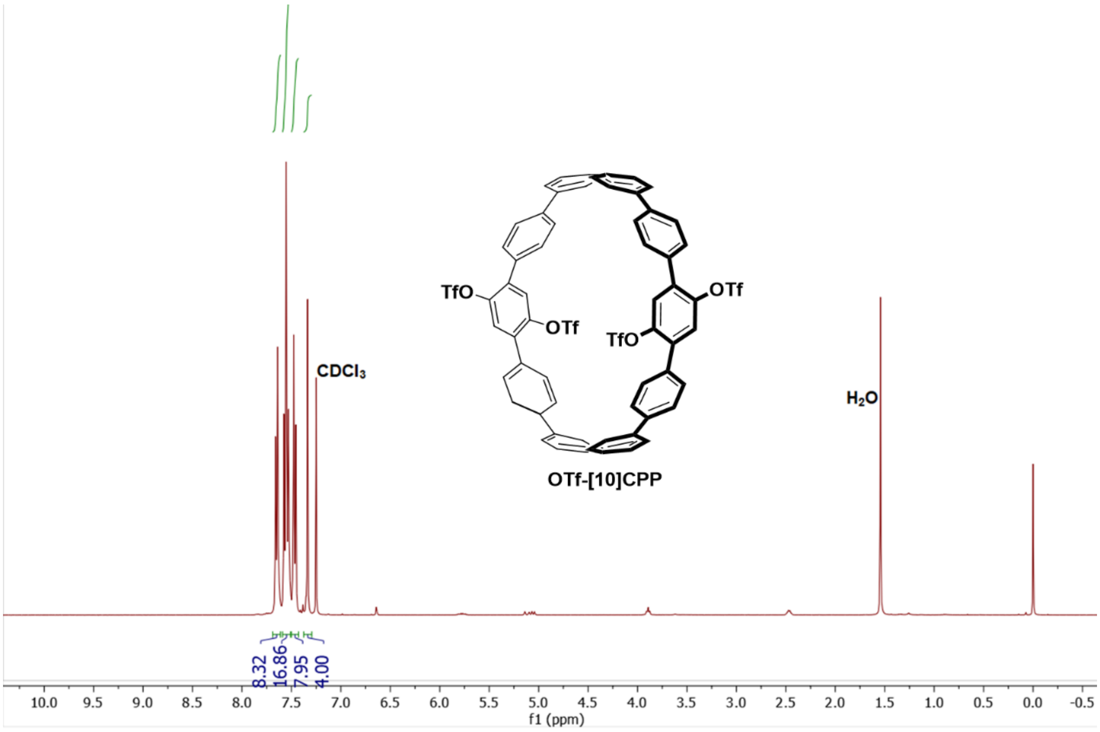

Figure **S39**. ^{1}H NMR (400 MHz) of **OTf-[10]CPP** in $CDCl_3$.

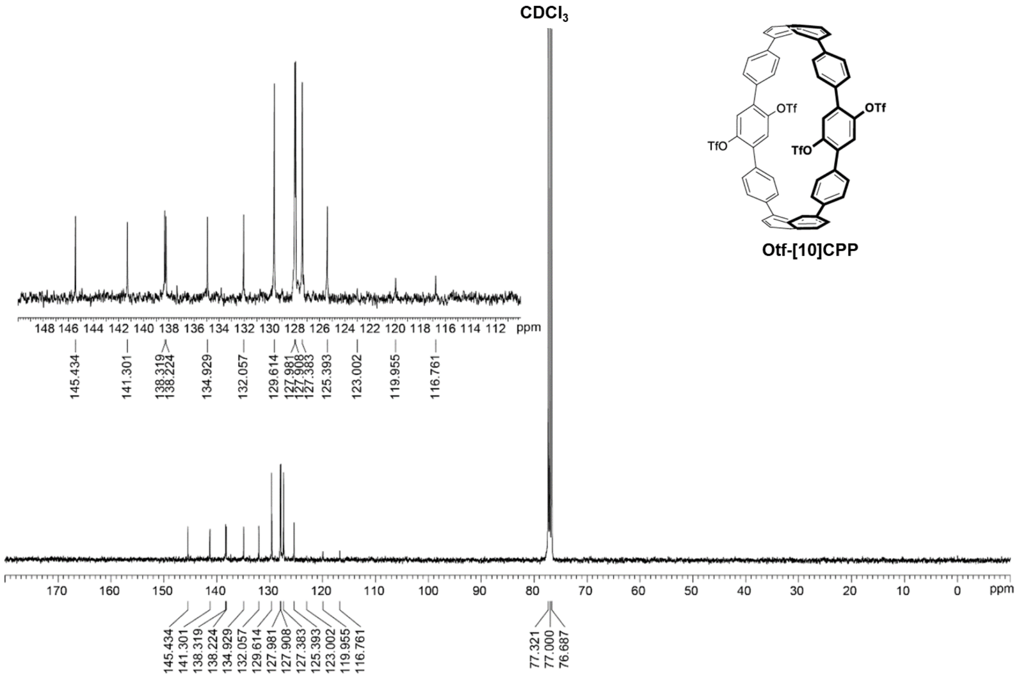

Figure **S40**. ^{13}C NMR (100 MHz) of **OTf-[10]CPP** in $CDCl_3$.

Ground State Optimization Summary

[10]CPP

Total energy = -2308.02249526 a.u.

RMS Gradient Norm = 0.000007 a.u.

Number of Imaginary Frequency = 0

Dipole Moment = 0.009774 Debye

4PXZPh-[10]CPP

Total energy = -5594.56204605 a.u.

RMS Gradient Norm = 0.000002 a.u.

Number of Imaginary Frequency = 0

Dipole Moment = 0.154189 Debye

Cartesian Coordinates

Ground state geometry

Center Number	Atomic Number	Atomic Type	Coordinates (Angstroms) X	Y	Z
1	6	0	-2.624931	-1.574342	-6.578245
2	6	0	-5.076631	-1.887061	-2.968594
3	6	0	-6.701134	-1.610332	0.917543
4	6	0	-5.513170	-0.980164	4.945562
5	6	0	-1.362055	-1.222228	6.532441
6	6	0	2.856274	-1.103481	6.621678
7	6	0	5.340191	-1.006682	3.024715
8	6	0	6.883205	-0.452289	-0.867216
9	6	0	5.594194	-0.029417	-4.899055
10	6	0	1.549665	-0.974220	-6.498133
11	6	0	-1.337967	-1.348519	-7.044579
12	6	0	-4.290812	-1.816120	-4.103957
13	6	0	-6.720179	-1.902629	-0.445006
14	6	0	-6.324633	-1.026877	3.816179
15	6	0	-2.676938	-1.225055	6.091458
16	6	0	1.547648	-1.099702	7.082569

17	6	0	4.551494	-1.066425	4.158883
18	6	0	6.956519	-0.736413	0.495513
19	6	0	6.401358	0.060070	-3.768906
20	6	0	2.844279	-0.753736	-6.052621
21	6	0	-4.485427	-0.810790	-5.059858
22	6	0	-6.663299	-0.833658	-1.369320
23	6	0	-6.203828	-0.074538	2.794052
24	6	0	-3.416822	-0.038503	6.034458
25	6	0	0.738431	0.039163	6.951020
26	6	0	4.565611	-0.036634	5.108620
27	6	0	6.713697	0.306118	1.419628
28	6	0	6.117585	0.972815	-2.742875
29	6	0	3.369988	0.541705	-5.990543
30	6	0	-0.734280	-0.088073	-6.916950
31	6	0	-3.369928	-0.542967	-5.990512
32	6	0	-6.117647	-0.973142	-2.742838
33	6	0	-6.713683	-0.305672	1.419556
34	6	0	-4.565648	0.037271	5.108593
35	6	0	-0.738472	-0.038652	6.951041
36	6	0	3.416801	0.039097	6.034510
37	6	0	6.203837	0.075088	2.794125
38	6	0	6.663266	0.833650	-1.369331

39	6	0	4.485427	0.809803	-5.059886
40	6	0	0.734394	0.086489	-6.916950
41	6	0	-2.844158	0.752436	-6.052871
42	6	0	-6.401231	-0.060496	-3.769010
43	6	0	-6.956314	0.736753	0.495267
44	6	0	-4.551497	1.067013	4.158812
45	6	0	-1.547681	1.100211	7.082635
46	6	0	2.676900	1.225634	6.091556
47	6	0	6.324605	1.027482	3.816195
48	6	0	6.719855	1.902771	-0.445181
49	6	0	4.290632	1.815258	-4.104154
50	6	0	1.338054	1.346917	-7.044912
51	6	0	-1.549539	0.972771	-6.498451
52	6	0	-5.594061	0.028634	-4.899181
53	6	0	-6.882960	0.452407	-0.867414
54	6	0	-5.340162	1.007223	3.024622
55	6	0	-2.856280	1.104042	6.621683
56	6	0	1.362017	1.222776	6.532524
57	6	0	5.513099	0.980824	4.945556
58	6	0	6.700845	1.610684	0.917412
59	6	0	5.076411	1.886511	-2.968787
60	6	0	2.624970	1.572915	-6.578535

61	1	0	-3.038791	-2.578850	-6.611732
62	1	0	-4.811809	-2.604308	-2.201621
63	1	0	-6.591805	-2.438352	1.612097
64	1	0	-5.586684	-1.768496	5.689896
65	1	0	-0.773566	-2.127022	6.418549
66	1	0	3.434850	-2.022823	6.658519
67	1	0	5.208689	-1.763588	2.261034
68	1	0	6.922802	-1.286238	-1.561995
69	1	0	5.804030	-0.789509	-5.646809
70	1	0	1.124086	-1.966419	-6.387108
71	1	0	-0.769193	-2.180396	-7.449710
72	1	0	-3.429055	-2.471349	-4.191752
73	1	0	-7.027322	-1.848401	3.705626
74	1	0	-3.087720	-2.128345	5.649622
75	1	0	1.126980	-2.015810	7.487098
76	1	0	3.816849	-1.861069	4.250127
77	1	0	7.236460	-0.627083	-3.662078
78	1	0	3.401976	-1.574577	-5.610962
79	1	0	-3.401848	1.573422	-5.611469
80	1	0	-7.236195	0.626844	-3.662292
81	1	0	-3.816815	1.861629	4.250003
82	1	0	-1.127007	2.016301	7.487198

83	1	0	3.087694	2.128958	5.649802
84	1	0	7.027363	1.848951	3.705660
85	1	0	3.428777	2.470338	-4.192097
86	1	0	0.769254	2.178675	-7.450256
87	1	0	-1.123939	1.964992	-6.387693
88	1	0	-5.803747	0.788643	-5.647063
89	1	0	-5.208547	1.764056	2.260886
90	1	0	-3.434846	2.023390	6.658584
91	1	0	0.773530	2.127580	6.418707
92	1	0	5.586579	1.769180	5.689867
93	1	0	6.591226	2.438780	1.611828
94	1	0	4.811466	2.603844	-2.201937
95	1	0	3.038786	2.577433	-6.612226
96	6	0	7.207975	-2.122724	0.940799
97	6	0	6.548147	-3.208979	0.353561
98	6	0	8.090736	-2.365625	2.000205
99	6	0	6.714891	-4.494186	0.853562
100	1	0	5.847533	-3.033926	-0.457865
101	6	0	8.276348	-3.650047	2.488224
102	1	0	8.619226	-1.531449	2.450488
103	6	0	7.566960	-4.715324	1.934830
104	1	0	6.167813	-5.331271	0.430938

105	1	0	8.949129	-3.840588	3.318339
106	6	0	6.960983	-6.295554	3.682981
107	6	0	8.334358	-7.040235	1.838980
108	6	0	6.184060	-5.346464	4.347428
109	6	0	7.017762	-7.594864	4.209674
110	6	0	8.351056	-8.322092	2.409414
111	6	0	9.002495	-6.845371	0.630951
112	6	0	5.497851	-5.679643	5.514257
113	1	0	6.122017	-4.338765	3.951967
114	6	0	6.332353	-7.925732	5.364772
115	6	0	8.995670	-9.372231	1.781162
116	1	0	9.012966	-5.855586	0.188307
117	6	0	9.655706	-7.902976	-0.000115
118	6	0	5.569252	-6.965822	6.028744
119	1	0	4.907080	-4.918191	6.014207
120	1	0	6.410926	-8.944878	5.728633
121	6	0	9.653001	-9.168344	0.568243
122	1	0	8.975539	-10.343787	2.263743
123	1	0	10.168894	-7.723093	-0.939387
124	1	0	5.038906	-7.231735	6.937057
125	1	0	10.158480	-9.996284	0.082481
126	7	0	7.674692	-6.006321	2.511979

127	8	0	7.750912	-8.589914	3.613984
128	1	0	-6.922367	1.286252	-1.562330
129	6	0	6.709089	3.310391	-0.894244
130	6	0	7.534242	3.712075	-1.951307
131	6	0	5.839459	4.249155	-0.326277
132	6	0	7.453590	4.997891	-2.464254
133	1	0	8.223806	2.995625	-2.386195
134	6	0	5.739129	5.530775	-0.852021
135	1	0	5.182632	3.947361	0.484594
136	6	0	6.531154	5.901131	-1.938373
137	1	0	8.075059	5.306783	-3.298755
138	1	0	5.024584	6.242512	-0.450091
139	6	0	6.718310	8.345078	-1.907667
140	6	0	5.560373	7.239662	-3.716952
141	6	0	7.401901	8.352612	-0.692682
142	6	0	6.413934	9.576509	-2.507395
143	6	0	5.290630	8.499194	-4.272916
144	6	0	5.018651	6.113819	-4.337773
145	6	0	7.757856	9.554923	-0.083476
146	1	0	7.661048	7.407707	-0.228158
147	6	0	6.761847	10.769503	-1.900149
148	6	0	4.516856	8.624561	-5.412802

149	1	0	5.210915	5.131146	-3.921876
150	6	0	4.244830	6.240727	-5.490243
151	6	0	7.437756	10.765598	-0.680436
152	1	0	8.291271	9.531736	0.861441
153	1	0	6.498842	11.693336	-2.404695
154	6	0	3.991535	7.491748	-6.033360
155	1	0	4.339041	9.622450	-5.799869
156	1	0	3.843759	5.345934	-5.956616
157	1	0	7.710598	11.705069	-0.211523
158	1	0	3.391141	7.597374	-6.930810
159	6	0	-7.207527	2.123170	0.940362
160	6	0	-8.090256	2.366348	1.999733
161	6	0	-6.547413	3.209227	0.353080
162	6	0	-8.275500	3.650830	2.487726
163	1	0	-8.618975	1.532328	2.450039
164	6	0	-6.713788	4.494491	0.853059
165	1	0	-5.846824	3.033953	-0.458320
166	6	0	-7.565753	4.715886	1.934353
167	1	0	-8.948223	3.841571	3.317844
168	1	0	-6.166443	5.331407	0.430447
169	6	0	-6.709696	-3.310312	-0.893880
170	6	0	-7.534827	-3.711930	-1.950986

171	6	0	-5.840340	-4.249202	-0.325704
172	6	0	-7.454374	-4.997808	-2.463813
173	1	0	-8.224186	-2.995372	-2.386019
174	6	0	-5.740223	-5.530896	-0.851312
175	1	0	-5.183546	-3.947470	0.485216
176	6	0	-6.532185	-5.901177	-1.937731
177	1	0	-8.075793	-5.306650	-3.298369
178	1	0	-5.025878	-6.242749	-0.449233
179	6	0	-8.332865	7.040907	1.838872
180	6	0	-6.959758	6.295731	3.682895
181	6	0	-9.001286	6.846209	0.630976
182	6	0	-8.349402	8.322690	2.409477
183	6	0	-7.016376	7.594978	4.209771
184	6	0	-6.183372	5.346318	4.347512
185	6	0	-9.654612	7.903910	0.000190
186	1	0	-9.011860	5.856482	0.188204
187	6	0	-8.994166	9.372914	1.781523
188	6	0	-6.331379	7.925449	5.365228
189	1	0	-6.121428	4.338670	3.951911
190	6	0	-5.497544	5.679113	5.514676
191	6	0	-9.651766	9.169201	0.568720
192	1	0	-10.167999	7.724162	-0.939000

193	1	0	-8.973916	10.344405	2.264230
194	6	0	-5.568810	6.965221	6.029354
195	1	0	-6.409820	8.944556	5.729225
196	1	0	-4.907181	4.917414	6.014732
197	1	0	-10.157331	9.997217	0.083179
198	1	0	-5.038765	7.230841	6.937928
199	6	0	-6.719648	-8.345086	-1.906783
200	6	0	-5.561381	-7.240003	-3.716057
201	6	0	-7.403369	-8.352391	-0.691871
202	6	0	-6.415289	-9.576623	-2.506303
203	6	0	-5.291637	-8.499630	-4.271803
204	6	0	-5.019531	-6.114274	-4.336978
205	6	0	-7.759500	-9.554591	-0.082546
206	1	0	-7.662472	-7.407392	-0.227512
207	6	0	-6.763379	-10.769503	-1.898940
208	6	0	-4.517701	-8.625209	-5.411556
209	1	0	-5.211829	-5.131531	-3.921263
210	6	0	-4.245559	-6.241396	-5.489321
211	6	0	-7.439436	-10.765373	-0.679309
212	1	0	-8.293022	-9.531236	0.862307
213	1	0	-6.500382	-11.693430	-2.403319
214	6	0	-3.992238	-7.492513	-6.032207

215	1	0	-4.339892	-9.623161	-5.798463
216	1	0	-3.844384	-5.346697	-5.955785
217	1	0	-7.712424	-11.704758	-0.210306
218	1	0	-3.391708	-7.598300	-6.929548
219	7	0	6.353302	7.163061	-2.562025
220	7	0	-7.673001	6.006907	2.511518
221	7	0	-6.354469	-7.163191	-2.561260
222	8	0	5.783063	9.655889	-3.723375
223	8	0	-7.748860	8.590394	3.613873
224	8	0	-5.784266	-9.656211	-3.722193

S_1 state geometry

Center Number	Atomic Number	Atomic Type	Coordinates (Angstroms) X	Y	Z
1	6	0	-2.524931	-1.534342	-6.578245
2	6	0	-5.076631	-1.887061	-2.968594
3	6	0	-6.701134	-1.630332	0.917543
4	6	0	-5.513170	-0.980164	4.945562
5	6	0	-1.362055	-1.222228	6.532441
6	6	0	2.856274	-1.103481	6.621678

7	6	0	5.340191	-1.006682	3.024715
8	6	0	6.883205	-0.452289	-0.867216
9	6	0	5.594194	-0.029417	-4.899055
10	6	0	1.549665	-0.974220	-6.498133
11	6	0	-1.337967	-1.348519	-7.044579
12	6	0	-4.290812	-1.816120	-4.103957
13	6	0	-6.720179	-1.902629	-0.445006
14	6	0	-6.324633	-1.026877	3.816179
15	6	0	-2.676938	-1.225055	6.091458
16	6	0	1.547648	-1.099702	7.082569
17	6	0	4.551494	-1.066425	4.158883
18	6	0	6.956519	-0.736413	0.495513
19	6	0	6.401358	0.060070	-3.768906
20	6	0	2.844279	-0.753736	-6.052621
21	6	0	-4.485427	-0.810790	-5.059858
22	6	0	-6.663299	-0.833658	-1.369320
23	6	0	-6.203828	-0.074538	2.794052
24	6	0	-3.416822	-0.038503	6.034458
25	6	0	0.738431	0.039163	6.951020
26	6	0	4.565611	-0.036634	5.108620
27	6	0	6.713697	0.306118	1.419628
28	6	0	6.117585	0.972815	-2.742875

29	6	0	3.369988	0.541705	-5.990543
30	6	0	-0.734280	-0.088073	-6.916950
31	6	0	-3.369928	-0.542967	-5.990512
32	6	0	-6.117647	-0.973142	-2.742838
33	6	0	-6.713683	-0.305672	1.419556
34	6	0	-4.565648	0.037271	5.108593
35	6	0	-0.738472	-0.038652	6.951041
36	6	0	3.416801	0.039097	6.034510
37	6	0	6.203837	0.075088	2.794125
38	6	0	6.663266	0.833650	-1.369331
39	6	0	4.485427	0.809803	-5.059886
40	6	0	0.734394	0.086489	-6.916950
41	6	0	-2.844158	0.752436	-6.052871
42	6	0	-6.401231	-0.060496	-3.769010
43	6	0	-6.956314	0.736753	0.495267
44	6	0	-4.551497	1.067013	4.158812
45	6	0	-1.547681	1.100211	7.082635
46	6	0	2.676900	1.225634	6.091556
47	6	0	6.324605	1.027482	3.816195
48	6	0	6.719855	1.902771	-0.445181
49	6	0	4.290632	1.815258	-4.104154
50	6	0	1.338054	1.346917	-7.044912

51	6	0	-1.549539	0.972771	-6.498451
52	6	0	-5.594061	0.028634	-4.899181
53	6	0	-6.882960	0.452407	-0.867414
54	6	0	-5.340162	1.007223	3.024622
55	6	0	-2.856280	1.104042	6.621683
56	6	0	1.362017	1.222776	6.532524
57	6	0	5.513099	0.980824	4.945556
58	6	0	6.700845	1.610684	0.917412
59	6	0	5.076411	1.886511	-2.968787
60	6	0	2.624970	1.572915	-6.578535
61	1	0	-3.038791	-2.578850	-6.611732
62	1	0	-4.811809	-2.604308	-2.201621
63	1	0	-6.591805	-2.438352	1.612097
64	1	0	-5.586684	-1.768496	5.689896
65	1	0	-0.773566	-2.127022	6.418549
66	1	0	3.434850	-2.022823	6.658519
67	1	0	5.208689	-1.763588	2.261034
68	1	0	6.922802	-1.286238	-1.561995
69	1	0	5.804030	-0.789509	-5.646809
70	1	0	1.124086	-1.966419	-6.387108
71	1	0	-0.769193	-2.180396	-7.449710
72	1	0	-3.429055	-2.471349	-4.191752

73	1	0	-7.027322	-1.848401	3.705626
74	1	0	-3.087720	-2.128345	5.649622
75	1	0	1.126980	-2.015810	7.487098
76	1	0	3.816849	-1.861069	4.250127
77	1	0	7.236460	-0.627083	-3.662078
78	1	0	3.401976	-1.574577	-5.610962
79	1	0	-3.401848	1.573422	-5.611469
80	1	0	-7.236195	0.626844	-3.662292
81	1	0	-3.816815	1.861629	4.250003
82	1	0	-1.127007	2.016301	7.487198
83	1	0	3.087694	2.128958	5.649802
84	1	0	7.027363	1.848951	3.705660
85	1	0	3.428777	2.470338	-4.192097
86	1	0	0.769254	2.178675	-7.450256
87	1	0	-1.123939	1.964992	-6.387693
88	1	0	-5.803747	0.788643	-5.647063
89	1	0	-5.208547	1.764056	2.260886
90	1	0	-3.434846	2.023390	6.658584
91	1	0	0.773530	2.127580	6.418707
92	1	0	5.586579	1.769180	5.689867
93	1	0	6.591226	2.438780	1.611828
94	1	0	4.811466	2.603844	-2.201937

95	1	0	3.038786	2.577433	-6.612226
96	6	0	7.207975	-2.122724	0.940799
97	6	0	6.548147	-3.208979	0.353561
98	6	0	8.090736	-2.365625	2.000205
99	6	0	6.714891	-4.494186	0.853562
100	1	0	5.847533	-3.033926	-0.457865
101	6	0	8.276348	-3.650047	2.488224
102	1	0	8.619226	-1.531449	2.450488
103	6	0	7.566960	-4.715324	1.934830
104	1	0	6.167813	-5.331271	0.430938
105	1	0	8.949129	-3.840588	3.318339
106	6	0	6.960983	-6.295554	3.682981
107	6	0	8.334358	-7.040235	1.838980
108	6	0	6.184060	-5.346464	4.347428
109	6	0	7.017762	-7.594864	4.209674
110	6	0	8.351056	-8.322092	2.409414
111	6	0	9.002495	-6.845371	0.630951
112	6	0	5.497851	-5.679643	5.514257
113	1	0	6.122017	-4.338765	3.951967
114	6	0	6.332353	-7.925732	5.364772
115	6	0	8.995670	-9.372231	1.781162
116	1	0	9.012966	-5.855586	0.188307

117	6	0	9.655706	-7.902976	-0.000115
118	6	0	5.569252	-6.965822	6.028744
119	1	0	4.907080	-4.918191	6.014207
120	1	0	6.410926	-8.944878	5.728633
121	6	0	9.653001	-9.168344	0.568243
122	1	0	8.975539	-10.343787	2.263743
123	1	0	10.168894	-7.723093	-0.939387
124	1	0	5.038906	-7.231735	6.937057
125	1	0	10.158480	-9.996284	0.082481
126	7	0	7.674692	-6.006321	2.511979
127	8	0	7.750912	-8.589914	3.613984
128	1	0	-6.922367	1.286252	-1.562330
129	6	0	6.709089	3.310391	-0.894244
130	6	0	7.534242	3.712075	-1.951307
131	6	0	5.839459	4.249155	-0.326277
132	6	0	7.453590	4.997891	-2.464254
133	1	0	8.223806	2.995625	-2.386195
134	6	0	5.739129	5.530775	-0.852021
135	1	0	5.182632	3.947361	0.484594
136	6	0	6.531154	5.901131	-1.938373
137	1	0	8.075059	5.306783	-3.298755
138	1	0	5.024584	6.242512	-0.450091

139	6	0	6.718310	8.345078	-1.907667
140	6	0	5.560373	7.239662	-3.716952
141	6	0	7.401901	8.352612	-0.692682
142	6	0	6.413934	9.576509	-2.507395
143	6	0	5.290630	8.499194	-4.272916
144	6	0	5.018651	6.113819	-4.337773
145	6	0	7.757856	9.554923	-0.083476
146	1	0	7.661048	7.407707	-0.228158
147	6	0	6.761847	10.769503	-1.900149
148	6	0	4.516856	8.624561	-5.412802
149	1	0	5.210915	5.131146	-3.921876
150	6	0	4.244830	6.240727	-5.490243
151	6	0	7.437756	10.765598	-0.680436
152	1	0	8.291271	9.531736	0.861441
153	1	0	6.498842	11.693336	-2.404695
154	6	0	3.991535	7.491748	-6.033360
155	1	0	4.339041	9.622450	-5.799869
156	1	0	3.843759	5.345934	-5.956616
157	1	0	7.710598	11.705069	-0.211523
158	1	0	3.391141	7.597374	-6.930810
159	6	0	-7.207527	2.123170	0.940362
160	6	0	-8.090256	2.366348	1.999733

161	6	0	-6.547413	3.209227	0.353080
162	6	0	-8.275500	3.650830	2.487726
163	1	0	-8.618975	1.532328	2.450039
164	6	0	-6.713788	4.494491	0.853059
165	1	0	-5.846824	3.033953	-0.458320
166	6	0	-7.565753	4.715886	1.934353
167	1	0	-8.948223	3.841571	3.317844
168	1	0	-6.166443	5.331407	0.430447
169	6	0	-6.709696	-3.310312	-0.893880
170	6	0	-7.534827	-3.711930	-1.950986
171	6	0	-5.840340	-4.249202	-0.325704
172	6	0	-7.454374	-4.997808	-2.463813
173	1	0	-8.224186	-2.995372	-2.386019
174	6	0	-5.740223	-5.530896	-0.851312
175	1	0	-5.183546	-3.947470	0.485216
176	6	0	-6.532185	-5.901177	-1.937731
177	1	0	-8.075793	-5.306650	-3.298369
178	1	0	-5.025878	-6.242749	-0.449233
179	6	0	-8.332865	7.040907	1.838872
180	6	0	-6.959758	6.295731	3.682895
181	6	0	-9.001286	6.846209	0.630976
182	6	0	-8.349402	8.322690	2.409477

183	6	0	-7.016376	7.594978	4.209771
184	6	0	-6.183372	5.346318	4.347512
185	6	0	-9.654612	7.903910	0.000190
186	1	0	-9.011860	5.856482	0.188204
187	6	0	-8.994166	9.372914	1.781523
188	6	0	-6.331379	7.925449	5.365228
189	1	0	-6.121428	4.338670	3.951911
190	6	0	-5.497544	5.679113	5.514676
191	6	0	-9.651766	9.169201	0.568720
192	1	0	-10.167999	7.724162	-0.939000
193	1	0	-8.973916	10.344405	2.264230
194	6	0	-5.568810	6.965221	6.029354
195	1	0	-6.409820	8.944556	5.729225
196	1	0	-4.907181	4.917414	6.014732
197	1	0	-10.157331	9.997217	0.083179
198	1	0	-5.038765	7.230841	6.937928
199	6	0	-6.719648	-8.345086	-1.906783
200	6	0	-5.561381	-7.240003	-3.716057
201	6	0	-7.403369	-8.352391	-0.691871
202	6	0	-6.415289	-9.576623	-2.506303
203	6	0	-5.291637	-8.499630	-4.271803
204	6	0	-5.019531	-6.114274	-4.336978

205	6	0	-7.759500	-9.554591	-0.082546
206	1	0	-7.662472	-7.407392	-0.227512
207	6	0	-6.763379	-10.769503	-1.898940
208	6	0	-4.517701	-8.625209	-5.411556
209	1	0	-5.211829	-5.131531	-3.921263
210	6	0	-4.245559	-6.241396	-5.489321
211	6	0	-7.439436	-10.765373	-0.679309
212	1	0	-8.293022	-9.531236	0.862307
213	1	0	-6.500382	-11.693430	-2.403319
214	6	0	-3.992238	-7.492513	-6.032207
215	1	0	-4.339892	-9.623161	-5.798463
216	1	0	-3.844384	-5.346697	-5.955785
217	1	0	-7.512694	-11.704758	-0.210306
218	1	0	-3.391708	-7.598300	-6.929548
219	7	0	6.353302	7.163061	-2.562025
220	7	0	-7.673001	6.006907	2.511518
221	7	0	-6.354469	-7.163191	-2.563276
222	8	0	5.783063	9.655889	-3.723372
223	8	0	-7.748860	8.590394	3.613866
224	8	0	-5.784266	-9.656211	-3.722191

T_1 state geometry

Center Number	Atomic Number	Atomic Type	Coordinates (Angstroms) X	Y	Z
1	6	0	-2.624931	-1.574342	-6.578245
2	6	0	-5.076631	-1.887061	-2.968594
3	6	0	-6.701134	-1.610332	0.917543
4	6	0	-5.513170	-0.980164	4.945562
5	6	0	-1.362055	-1.222228	6.532441
6	6	0	2.856274	-1.103481	6.621678
7	6	0	5.340191	-1.006682	3.024715
8	6	0	6.883205	-0.452289	-0.867216
9	6	0	5.594194	-0.029417	-4.899055
10	6	0	1.549665	-0.974220	-6.498133
11	6	0	-1.337967	-1.348519	-7.044579
12	6	0	-4.290812	-1.816120	-4.103957
13	6	0	-6.720179	-1.902629	-0.445006
14	6	0	-6.324633	-1.026877	3.816179
15	6	0	-2.676938	-1.225055	6.091458
16	6	0	1.547648	-1.099702	7.082569
17	6	0	4.551494	-1.066425	4.158883
18	6	0	6.956519	-0.736413	0.495513

19	6	0	6.401358	0.060070	-3.768906
20	6	0	2.844279	-0.753736	-6.052621
21	6	0	-4.485427	-0.810790	-5.059858
22	6	0	-6.663299	-0.833658	-1.369320
23	6	0	-6.203828	-0.074538	2.794052
24	6	0	-3.416822	-0.038503	6.034458
25	6	0	0.738431	0.039163	6.951020
26	6	0	4.565611	-0.036634	5.108620
27	6	0	6.713697	0.306118	1.419628
28	6	0	6.117585	0.972815	-2.742875
29	6	0	3.369988	0.541705	-5.990543
30	6	0	-0.734280	-0.088073	-6.916950
31	6	0	-3.369928	-0.542967	-5.990512
32	6	0	-6.117647	-0.973142	-2.742838
33	6	0	-6.713683	-0.305672	1.419556
34	6	0	-4.565648	0.037271	5.108593
35	6	0	-0.738472	-0.038652	6.951041
36	6	0	3.416801	0.039097	6.034510
37	6	0	6.203837	0.075088	2.794125
38	6	0	6.663266	0.833650	-1.369331
39	6	0	4.485427	0.809803	-5.059886
40	6	0	0.734394	0.086489	-6.916950

41	6	0	-2.844158	0.752436	-6.052871
42	6	0	-6.401231	-0.060496	-3.769010
43	6	0	-6.956314	0.736753	0.495267
44	6	0	-4.551497	1.067013	4.158812
45	6	0	-1.547681	1.100211	7.082635
46	6	0	2.676900	1.225634	6.091556
47	6	0	6.324605	1.027482	3.816195
48	6	0	6.719855	1.902771	-0.445181
49	6	0	4.290632	1.815258	-4.104154
50	6	0	1.338054	1.346917	-7.044912
51	6	0	-1.549539	0.972771	-6.498451
52	6	0	-5.594061	0.028634	-4.899181
53	6	0	-6.882960	0.452407	-0.867414
54	6	0	-5.340162	1.007223	3.024622
55	6	0	-2.856280	1.104042	6.621683
56	6	0	1.362017	1.222776	6.532524
57	6	0	5.513099	0.980824	4.945556
58	6	0	6.700845	1.610684	0.917412
59	6	0	5.076411	1.886511	-2.968787
60	6	0	2.624970	1.572915	-6.578535
61	1	0	-3.038791	-2.578850	-6.611732
62	1	0	-4.811809	-2.604308	-2.201621

63	1	0	-6.591805	-2.438352	1.612097
64	1	0	-5.586684	-1.768496	5.689896
65	1	0	-0.773566	-2.127022	6.418549
66	1	0	3.434850	-2.022823	6.658519
67	1	0	5.208689	-1.763588	2.261034
68	1	0	6.922802	-1.286238	-1.561995
69	1	0	5.804030	-0.789509	-5.646809
70	1	0	1.124086	-1.966419	-6.387108
71	1	0	-0.769193	-2.180396	-7.449710
72	1	0	-3.429055	-2.471349	-4.191752
73	1	0	-7.027322	-1.848401	3.705626
74	1	0	-3.087720	-2.128345	5.649622
75	1	0	1.126980	-2.015810	7.487098
76	1	0	3.816849	-1.861069	4.250127
77	1	0	7.236460	-0.627083	-3.662078
78	1	0	3.401976	-1.574577	-5.610962
79	1	0	-3.401848	1.573422	-5.611469
80	1	0	-7.236195	0.626844	-3.662292
81	1	0	-3.816815	1.861629	4.250003
82	1	0	-1.127007	2.016301	7.487198
83	1	0	3.087694	2.128958	5.649802
84	1	0	7.027363	1.848951	3.705660

85	1	0	3.428777	2.470338	-4.192097
86	1	0	0.769254	2.178675	-7.450256
87	1	0	-1.123939	1.964992	-6.387693
88	1	0	-5.803747	0.788643	-5.647063
89	1	0	-5.208547	1.764056	2.260886
90	1	0	-3.434846	2.023390	6.658584
91	1	0	0.773530	2.127580	6.418707
92	1	0	5.586579	1.769180	5.689867
93	1	0	6.591226	2.438780	1.611828
94	1	0	4.811466	2.603844	-2.201937
95	1	0	3.038786	2.577433	-6.612226
96	6	0	7.207975	-2.122724	0.940799
97	6	0	6.548147	-3.208979	0.353561
98	6	0	8.090736	-2.365625	2.000205
99	6	0	6.714891	-4.494186	0.853562
100	1	0	5.847533	-3.033926	-0.457865
101	6	0	8.276348	-3.650047	2.488224
102	1	0	8.619226	-1.531449	2.450488
103	6	0	7.566960	-4.715324	1.934830
104	1	0	6.167813	-5.331271	0.430938
105	1	0	8.949129	-3.840588	3.318339
106	6	0	6.960983	-6.295554	3.682981

107	6	0	8.334358	-7.040235	1.838980
108	6	0	6.184060	-5.346464	4.347428
109	6	0	7.017762	-7.594864	4.209674
110	6	0	8.351056	-8.322092	2.409414
111	6	0	9.002495	-6.845371	0.630951
112	6	0	5.497851	-5.679643	5.514257
113	1	0	6.122017	-4.338765	3.951967
114	6	0	6.332353	-7.925732	5.364772
115	6	0	8.995670	-9.372231	1.781162
116	1	0	9.012966	-5.855586	0.188307
117	6	0	9.655706	-7.902976	-0.000115
118	6	0	5.569252	-6.965822	6.028744
119	1	0	4.907080	-4.918191	6.014207
120	1	0	6.410926	-8.944878	5.728633
121	6	0	9.653001	-9.168344	0.568243
122	1	0	8.975539	-10.343787	2.263743
123	1	0	10.168894	-7.723093	-0.939387
124	1	0	5.038906	-7.231735	6.937057
125	1	0	10.158480	-9.996284	0.082481
126	7	0	7.674692	-6.006321	2.511979
127	8	0	7.750912	-8.589914	3.613984
128	1	0	-6.922367	1.286252	-1.562330

129	6	0	6.709089	3.310391	-0.894244
130	6	0	7.534242	3.712075	-1.951307
131	6	0	5.839459	4.249155	-0.326277
132	6	0	7.453590	4.997891	-2.464254
133	1	0	8.223806	2.995625	-2.386195
134	6	0	5.739129	5.530775	-0.852021
135	1	0	5.182632	3.947361	0.484594
136	6	0	6.531154	5.901131	-1.938373
137	1	0	8.075059	5.306783	-3.298755
138	1	0	5.024584	6.242512	-0.450091
139	6	0	6.718310	8.345078	-1.907667
140	6	0	5.560373	7.239662	-3.716952
141	6	0	7.401901	8.352612	-0.692682
142	6	0	6.413934	9.576509	-2.507395
143	6	0	5.290630	8.499194	-4.272916
144	6	0	5.018651	6.113819	-4.337773
145	6	0	7.757856	9.554923	-0.083476
146	1	0	7.661048	7.407707	-0.228158
147	6	0	6.761847	10.769503	-1.900149
148	6	0	4.516856	8.624561	-5.412802
149	1	0	5.210915	5.131146	-3.921876
150	6	0	4.244830	6.240727	-5.490243

151	6	0	7.437756	10.765598	-0.680436
152	1	0	8.291271	9.531736	0.861441
153	1	0	6.498842	11.693336	-2.404695
154	6	0	3.991535	7.491748	-6.033360
155	1	0	4.339041	9.622450	-5.799869
156	1	0	3.843759	5.345934	-5.956616
157	1	0	7.568904	11.74359	-0.211523
158	1	0	3.391141	7.597374	-6.930810
159	6	0	-7.24326	2.123170	0.940362
160	6	0	-8.090256	2.366348	1.999733
161	6	0	-6.547413	3.209227	0.353080
162	6	0	-8.275500	3.650830	2.487726
163	1	0	-8.618975	1.532328	2.450039
164	6	0	-6.713788	4.494491	0.853059
165	1	0	-5.846824	3.033953	-0.458320
166	6	0	-7.565753	4.715886	1.934353
167	1	0	-8.948223	3.841571	3.317844
168	1	0	-6.166443	5.331407	0.430447
169	6	0	-6.709696	-3.310312	-0.893880
170	6	0	-7.534827	-3.711930	-1.950986
171	6	0	-5.840340	-4.249202	-0.325704
172	6	0	-7.454374	-4.997808	-2.463813

173	1	0	-8.224186	-2.995372	-2.386019
174	6	0	-5.740223	-5.530896	-0.851312
175	1	0	-5.183546	-3.947470	0.485216
176	6	0	-6.532185	-5.901177	-1.937731
177	1	0	-8.075793	-5.306650	-3.298369
178	1	0	-5.025878	-6.242749	-0.449233
179	6	0	-8.332865	7.040907	1.838872
180	6	0	-6.959758	6.295731	3.682895
181	6	0	-9.001286	6.846209	0.630976
182	6	0	-8.349402	8.322690	2.409477
183	6	0	-7.016376	7.594978	4.209771
184	6	0	-6.183372	5.346318	4.347512
185	6	0	-9.654612	7.903910	0.000190
186	1	0	-9.011860	5.856482	0.188204
187	6	0	-8.994166	9.372914	1.781523
188	6	0	-6.331379	7.925449	5.365228
189	1	0	-6.121428	4.338670	3.951911
190	6	0	-5.497544	5.679113	5.514676
191	6	0	-9.651766	9.169201	0.568720
192	1	0	-10.167999	7.724162	-0.939000
193	1	0	-8.973916	10.344405	2.264230
194	6	0	-5.568810	6.965221	6.029354

195	1	0	-6.409820	8.944556	5.729225
196	1	0	-4.907181	4.917414	6.014732
197	1	0	-10.157331	9.997217	0.083179
198	1	0	-5.038765	7.230841	6.937928
199	6	0	-6.719648	-8.345086	-1.906783
200	6	0	-5.561381	-7.240003	-3.716057
201	6	0	-7.403369	-8.352391	-0.691871
202	6	0	-6.415289	-9.576623	-2.506303
203	6	0	-5.291637	-8.499630	-4.271803
204	6	0	-5.019531	-6.114274	-4.336978
205	6	0	-7.759500	-9.554591	-0.082546
206	1	0	-7.662472	-7.407392	-0.227512
207	6	0	-6.763379	-10.769503	-1.898940
208	6	0	-4.517701	-8.625209	-5.411556
209	1	0	-5.211829	-5.131531	-3.921263
210	6	0	-4.245559	-6.241396	-5.489321
211	6	0	-7.439436	-10.765373	-0.679309
212	1	0	-8.293022	-9.531236	0.862307
213	1	0	-6.500382	-11.693430	-2.403319
214	6	0	-3.992238	-7.492513	-6.032207
215	1	0	-4.339892	-9.623161	-5.798463
216	1	0	-3.844384	-5.346697	-5.955785

217	1	0	-7.712424	-11.704758	-0.210306
218	1	0	-3.391708	-7.598300	-6.929548
219	7	0	6.353302	7.163061	-2.562025
220	7	0	-7.673001	6.006907	2.511518
221	7	0	-6.354469	-7.163191	-2.458979
222	8	0	5.783063	9.655889	-3.723375
223	8	0	-7.748860	8.590394	3.613873
224	8	0	-5.784266	-9.656211	-3.722193

References

(1) Kitamoto, Y.; Namikawa, T.; Ikemizu, D.; Miyata, Y.; Suzuki, T.; Kita, H.; Sato, T.; Oi, S. Light Blue and Green Thermally Activated Delayed Fluorescence from 10H-Phenoxaborin-Derivatives and Their Application to Organic Light-Emitting Diodes. *J. Mater. Chem. C* **2015**, *3* (35), 9122–9130. https://doi.org/10.1039/C5TC01380A.

(2) Park, I. S.; Lee, S. Y.; Adachi, C.; Yasuda, T. Full-Color Delayed Fluorescence Materials Based on Wedge-Shaped Phthalonitriles and Dicyanopyrazines: Systematic Design, Tunable Photophysical Properties, and OLED Performance. *Adv. Funct. Mater.* **2016**, *26* (11), 1813–1821. https://doi.org/10.1002/adfm.201505106.

(3) Kayahara, E.; Sun, L.; Onishi, H.; Suzuki, K.; Fukushima, T.; Sawada, A.; Kaji, H.; Yamago, S. Gram-Scale Syntheses and Conductivities of [10]Cycloparaphenylene and Its Tetraalkoxy Derivatives. *J. Am. Chem. Soc.* **2017**, *139* (51), 18480–18483. https://doi.org/10.1021/jacs.7b11526.

(4) Kayahara, E.; Nakano, M.; Sun, L.; Ishida, K.; Yamago, S. Syntheses of Tetrasubstituted [10]Cycloparaphenylenes by a Pd-Catalyzed Coupling Reaction. Remarkable Effect of Strain on the Oxidative Addition and Reductive Elimination. *Chem. - An Asian J.* **2020**, *15* (16), 2451–2455. https://doi.org/10.1002/asia.202000711.

(5) Adamo, C.; Barone, V. Toward Reliable Density Functional Methods without Adjustable Parameters: The PBE0 Model. *J. Chem. Phys.* **1999**, *110* (13), 6158–6170. https://doi.org/10.1063/1.478522.

(6) Pople, J. A.; Binkley, J. S.; Seeger, R. Theoretical Models Incorporating Electron Correlation. *Int. J. Quantum Chem.* **1976**, *10*, 1–19. https://doi.org/10.1002/qua.560100802.

(7) Hirata, S.; Head-Gordon, M. Time-Dependent Density Functional Theory within the Tamm–Dancoff Approximation. *Chem. Phys. Lett.* **1999**, *314*, 291–299.

(8) Grimme, S. Density Functional Calculations with Configuration Interaction for the Excited States of Molecules. *Chem. Phys. Lett.* **1996**, *259*, 128–137.

(9) Frisch, M. J.; Trucks, G. W.; Schlegel, H. B.; Scuseria, G. E.; Robb, M. A.; Cheeseman, J. R.; Scalmani, G.; Barone, V.; Mennucci, B.; Petersson, G. A.; Nakatsuji, H.; Caricato, M.; Li, X.; Hratchian, H. P.; Izmaylov, A. F.; Bloino, J.; Zheng, G.; Sonnenberg, J. L.; Had, M.; Fox, D. J. Gaussian 09, Revis. D.01. Wallingford, CT. 2009.

(10) Moral, M.; Muccioli, L.; Son, W.-J.; Olivier, Y.; Sancho-García, J. C. Theoretical Rationalization of the Singlet–Triplet Gap in OLEDs Materials: Impact of Charge-Transfer Character. *J. Chem. Theory Comput.* **2015**, *11* (1), 168–177.

(11) Gritzner, G.; Kuta, J. Recommendations on Reporting Electrode Potentials in

Nonaqueous Solvents (Recommendations 1983). *Pure Appl. Chem.* **1984**, *56* (4), 461–466. https://doi.org/10.1351/pac198456040461.

(12) Demas, J. N.; Crosby, G. A. The Measurement of Photolumineseence Quantum Yields. A Review. *J. Phys. Chem.* **1971**, *75* (8), 991–1024. https://doi.org/10.1021/j100678a001.

(13) Melhuish, W. H. Quantum Efficiencies of Fluorescence of Organic Substan Ces: Effect of Solvent and Concentration of the Fluorescent Solute. *J. Phys. Chem.* **1961**, *65* (2), 229–235. https://doi.org/10.1021/j100820a009.
